# Optotagging in primate cortex uncovers excitatory and inhibitory neuron specializations in form vision

**DOI:** 10.1038/s41467-026-76521-4

**Published:** 2026-08-26

**Authors:** Chuyi Su, Yasmine El-Shamayleh

**Affiliations:** 1 Zuckerman Mind Brain Behavior Institute, Columbia University, New York, NY USA; 2 Department of Neuroscience, Columbia University, New York, NY USA; 3 Kavli Institute for Brain Science, Columbia University, New York, NY USA

**Keywords:** Extrastriate cortex, Object vision, Optogenetics

## Abstract

The perception of complex visual shapes is a hallmark of primates and a foundation for many of their decisions and actions. The neural mechanisms of this visual function are best studied in macaques, but progress in this animal model has been hampered by the inability to dissect the contributions of distinct neuronal cell types. Here, we developed and applied an optogenetic approach for identifying the responses of individual excitatory and inhibitory neurons in the macaque visual cortex with light. Using this *optotagging* approach in male macaques, we uncovered clear functional differences between the two neuronal cell types. Excitatory and inhibitory neurons showed moderate differences in their extracellular spike waveforms and considerable differences in their visual response properties. Inhibitory neurons signaled simple visual forms almost exclusively, whereas excitatory neurons signaled both simple and complex forms and were more visually responsive than inhibitory neurons. Excitatory neurons had protracted response dynamics and developed their selectivity for complex forms gradually over time. These findings reveal a dominant role for excitatory neurons in visual shape processing, advancing a deeper mechanistic understanding of high-level vision. Beyond these insights, this work establishes a blueprint for elucidating the cell type-specific circuit logic underlying higher brain functions in primates.

## Abstract (web summary)

This study leverages cell type-specific optogenetics in the monkey cerebral cortex to elucidate the unique functional contributions of excitatory and inhibitory neurons in visual form processing. The findings contribute to a more detailed mechanistic understanding of high-level vision in primates.

## Introduction

Over decades, neuroscience research in macaque monkeys has made fundamental discoveries regarding how the primate cerebral cortex governs the perception of complex visual shapes^1–16^. This prior research has largely relied on experimental methods that are agnostic to the genetic identities of the cortical neurons under study. This traditional approach has produced invaluable insights into the visual shape processing algorithms performed by cortical neurons, but without clarifying precisely how these algorithms are implemented by various neuronal cell types. Here, we employ a fundamentally different approach, taking pivotal steps toward understanding the role of distinct cortical cell types in high-level vision.

Humans and other primates rely on visual shape information to identify and interact with objects in the world. Elucidating how primate cortical circuits process visual shape information is therefore essential for understanding how we perceive objects, and how we subsequently act on them. The neural mechanisms of this visual function are best studied in macaques because their perceptual abilities and cortical organization resemble those of humans. Neurophysiological studies in macaques have documented an abundance of neurons selective for visual shape information in higher areas of the cerebral cortex, starting in cortical area V4^1–3,6,9–11,13–17^. The shape-selective responses of V4 neurons are presumed to be a foundation for visual shape perception, yet we know little about their mechanistic implementation. Chiefly, we do not know how these shape-selective responses map on to the activity of two major cortical cell types—excitatory and inhibitory neurons classified on the basis of their neurotransmitter identity. Selectivity for visual shape information may be a shared property of excitatory and inhibitory neurons, or an exclusive property of one of these cell types. Achieving this finer grained knowledge is critical for advancing a comprehensive understanding of how V4 neurons acquire their visual shape preferences, and how their responses influence downstream circuits to mediate visual shape perception. Such mechanistic insights would also provide architectural constraints, currently lacking, for biologically inspired computational models of high-level vision^18–21^.

**Fig. 1:**
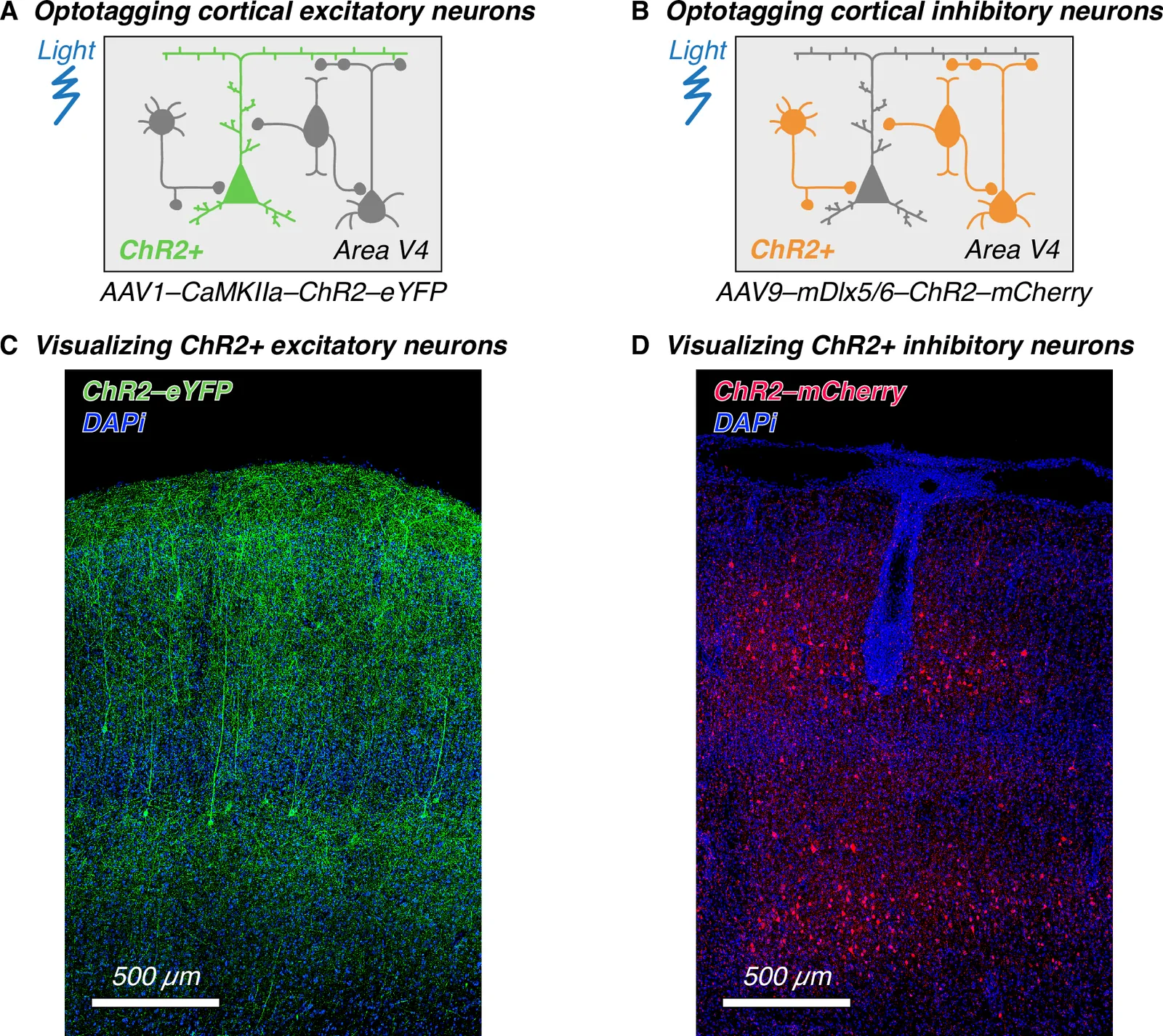
Experimental strategy. We used an optotagging approach to identify the spiking responses of excitatory and inhibitory neurons in macaque cortical area V4. We used AAV viral vectors with cell type-specific gene regulatory elements to express Channelrhodopsin-2 (ChR2) selectively in each neuronal cell type. **A** To target excitatory neurons (green), we expressed ChR2 under the control of the mouse CaMKIIa promoter. **B** To target inhibitory neurons (orange), we expressed ChR2 under the control of the mouse mDlx5/6 enhancer. In each data collection session, we identified the spiking activity of individual excitatory or inhibitory neurons based on their short-latency activation by blue light (450 nm). **C** Histological analysis of a V4 cortical site injected with AAV1–CaMKIIa–ChR2–eYFP (20X montage; scale bar corresponds to 500 µm). ChR2-positive neurons are in green; nuclei stained by DAPi are in blue. ChR2-positive neurons had pyramidal cell morphology, consistent with their classification as excitatory neurons. This histological section was taken several millimeters away from the injection site and processed with lower antibody concentration to reveal a few excitatory neuron cell bodies, without obstruction by the robust expression in apical and basal dendrites and long-range projection axons (see Supplementary Fig. S1; also see Methods for details). **D** Histological analysis of a V4 cortical site injected with AAV9–mDlx5/6–ChR2–mCherry (20X montage; scale bar corresponds to 500 µm). ChR2-positive neurons are in red; nuclei stained by DAPi are in blue. ChR2-positive neurons had non-pyramidal cell morphology, consistent with their classification as inhibitory neurons. Histological data (in **C**, **D**) were collected in one macaque (Monkey R; see Supplementary Table S1). At both injection sites, we observed many ChR2-positive neurons in the superficial and deep layers but not in the granular layer, consistent with previous studies. We also provide higher magnification images separately (see Supplementary Fig. S2).

To advance a deeper mechanistic understanding of visual shape perception in primates, we developed and applied an optogenetic approach for monitoring the responses of V4 excitatory and inhibitory neurons in macaques. We used adeno-associated viral vectors (AAVs) with cell type-specific gene regulatory elements to express the depolarizing opsin Channelrhodopsin-2 (ChR2) selectively in each neuronal cell type (Fig. 1; also see Supplementary Table S1). To target excitatory neurons, we expressed ChR2 under the control of the CaMKIIa promoter (Fig. 1A). To target inhibitory neurons, we expressed ChR2 under the control of the Dlx5/6 enhancer (Fig. 1B). Previous studies have validated the targeting specificity of AAVs containing the two gene regulatory elements in monkeys, including our own work: ~91–100% for the CaMKIIa promoter^22–25^ and ~86–93% for the Dlx5/6 enhancer^26–29^. We further confirmed the targeting specificity of AAV-mediated opsin expression histologically in one macaque. Consistent with previous studies, both AAVs transduced neurons in the superficial and deep layers but not in the granular layer; the CaMKII promoter restricted ChR2 expression to pyramidal cells, whereas the mDlx5/6 enhancer restricted ChR2 expression to non-pyramidal cells (Fig. 1C, D; also see Supplementary Figs. S1 and S2 for higher magnification images). We then used optical stimulation to identify the spiking responses of individual excitatory or inhibitory neurons in vivo, based on their short-latency activation by blue light—an approach herein referred to as *optotagging*.

Optotagging is routinely used in mice to probe the role of excitatory and inhibitory neurons in cortical function^30,31^, and this approach surpasses correlative measurements of extracellular waveforms currently used in monkeys to classify responses of the two cell types^32–34^. Optotagging studies conducted in mouse visual cortex have transformed our understanding of early visual processing, revealing that excitatory and inhibitory neurons are selective for basic visual properties (e.g., orientation, size, contrast), with some differences in their degree of selectivity^35–41^. However, more advanced aspects of primate vision, including the perception of complex shapes, are studied more comprehensively in macaques. Thus, developing a reliable optotagging approach in the macaque cerebral cortex would have broad utility for investigating the cellular and circuit mechanisms of high-level vision, as well as other primate-specific brain functions.

Leveraging the optotagging approach in the macaque cerebral cortex, we set out to probe the role of distinct neuronal cell types in visual shape processing. To this end, we asked how V4 excitatory and inhibitory neurons responded to two classes of visual form stimuli: simple and complex. The simple forms were bar stimuli commonly used to study neuronal selectivity for orientation in early visual cortical areas^42,43^. The complex forms were two-dimensional shape stimuli commonly used to study neuronal selectivity for contour shape in higher visual cortical areas^3,4,11,15,44^. We reasoned that by comparing the responses of V4 excitatory and inhibitory neurons to visual forms of different levels of complexity, we could discern potential functional specializations of the two neuronal cell types.

In this study, we characterized the responses of bona fide V4 excitatory and inhibitory neurons identified by optotagging, uncovering clear functional differences between them. The two neuronal cell types differed in their responses to optical and visual stimulation, and in their preferences for visual form. Inhibitory neurons were primarily selective for simple visual forms (i.e., bar orientation)—a property likely inherited from earlier cortical areas. Excitatory neurons were similarly selective for simple visual forms, yet a considerable proportion of them were also selective for complex visual forms (i.e., contour shape)—a property likely computed in V4 and downstream cortical areas. These findings provide critical insights into the relative contributions of the two neuronal cell types in visual shape processing, refining our understanding of the cells and circuits supporting the advanced visual abilities of primates. Additionally, this work serves as a blueprint for future macaque studies investigating the role of cortical excitatory and inhibitory neurons in higher sensory, motor and cognitive functions in primates.

## Results

**Fig. 2:**
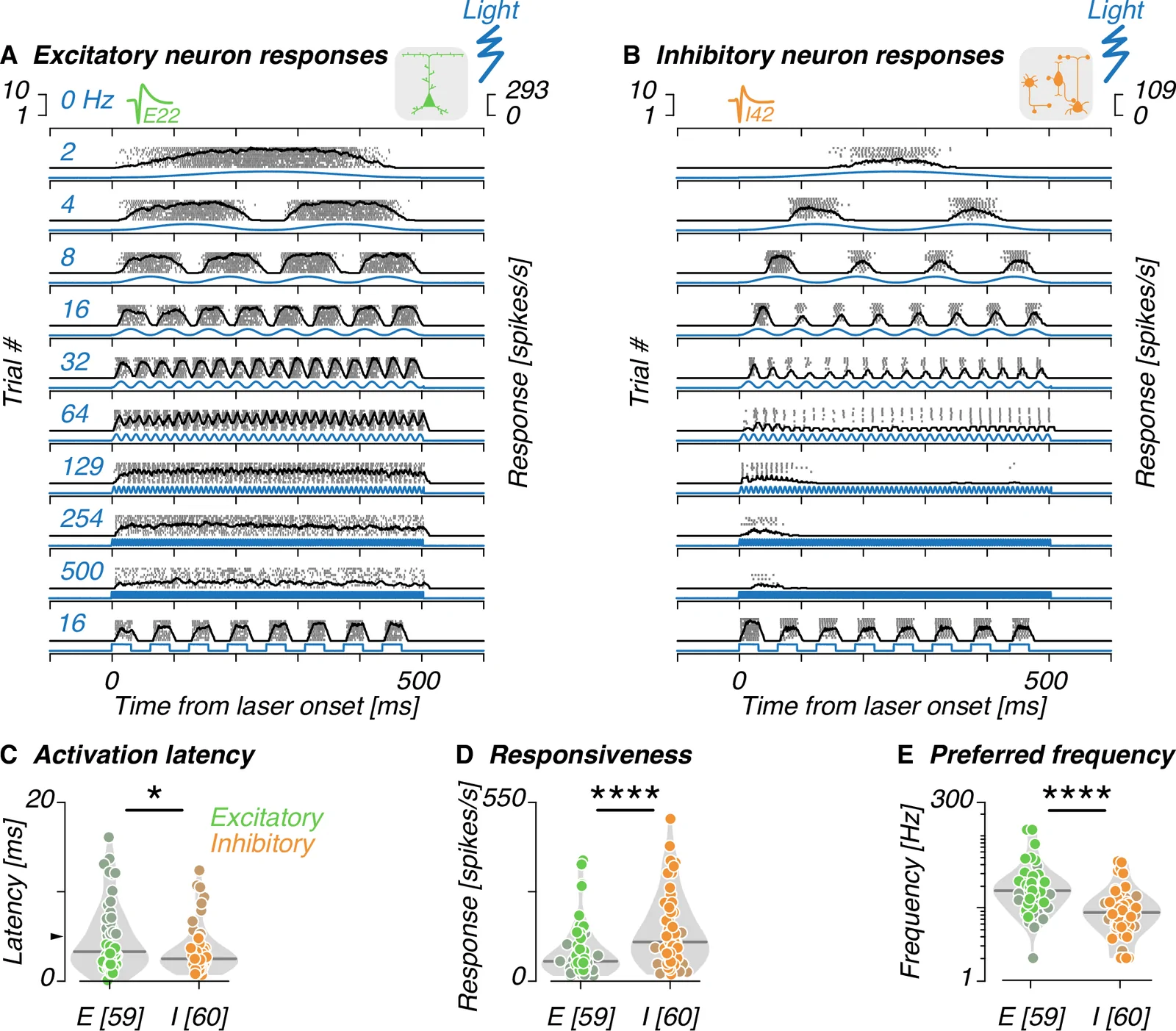
Responses of excitatory and inhibitory neurons to optical stimulation. We measured the responses of each optotagged neuron to various laser modulation patterns (sinusoidal 2–500 Hz; square wave 16 Hz). **A** Responses of an example excitatory neuron showing robust, short latency activation (activation latency was 2.9 ms; average spike waveform is shown in green). Laser modulation patterns (blue), raster plots (gray) and peri-stimulus time histograms (black) are shown. **B** Responses of an example inhibitory neuron showing robust, short latency activation (activation latency was 2.5 ms; average spike waveform is shown in orange; same format as in **A**). **C** Distribution of activation latencies for all optotagged neurons. Data from excitatory and inhibitory neurons are shown separately (green and orange). Data from neurons with short and long activation latencies are also shown separately (brighter and darker shades; arrowhead shows the 5 ms latency criterion). **D** Distribution of peak responses (*p* ≤ 0.0001). **E** Distribution of preferred laser modulation frequencies (*p* ≤ 0.0001). We also provide results for only the shortest latency optotagged neurons separately (<5 ms; see Supplementary Fig. S12). Violin plots in **C**–**E** show marginal distributions (gray), and median values (gray lines); sample sizes are indicated along the abscissa. Statistical comparisons were based on a Wilcoxon rank sum test.

**Fig. 3:**
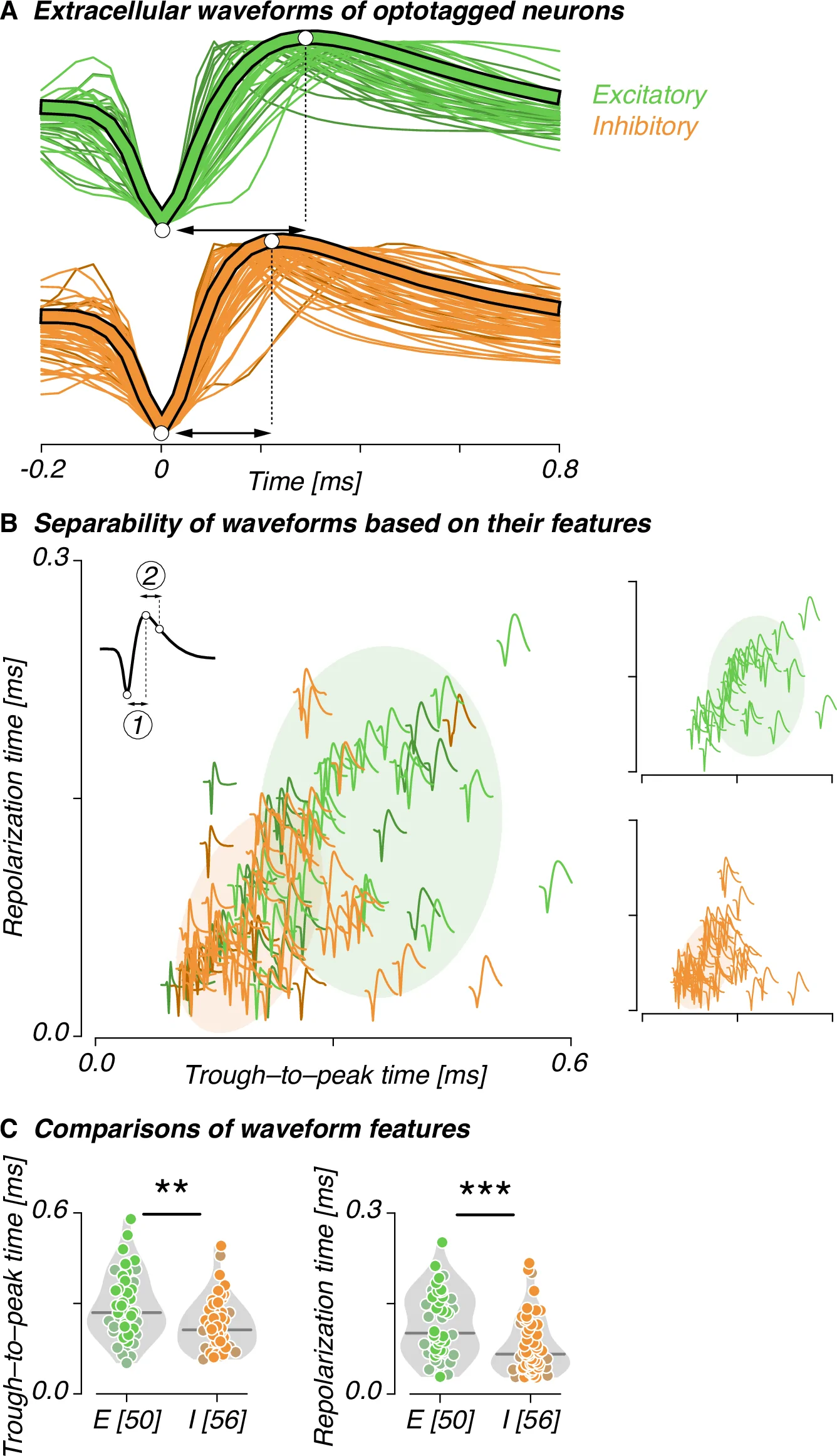
Extracellular waveforms of excitatory and inhibitory neurons. **A** Average waveforms of all optotagged excitatory and inhibitory neurons (green and orange; thin lines are average waveforms for each neuron; thick lines are global averages for each neuronal cell type; also see Supplementary Fig. S5 for the waveforms of individual neurons). **B** Separability of excitatory and inhibitory neuron waveforms. To compare the waveform properties of excitatory and inhibitory neurons, we computed two features: (i) trough–to–peak time and (ii) repolarization time (see inset). Left: average waveforms for all optotagged neurons, sorted by their waveform features (data are color-coded by neuronal cell type and activation latency). Ellipses represent the output of a k-means clustering procedure to partition waveforms into two clusters; ellipses show two times the 95% confidence estimates around cluster centroids. Right: average waveforms for only the short-latency neurons (<5 ms), shown separately for excitatory and inhibitory neurons. **C** Comparisons of waveform features for all optotagged neurons. Left: Distribution of trough–to–peak times (*p* ≤ 0.01). Sample sizes are indicated along the abscissa. Right: Distribution of repolarization times (*p* ≤ 0.001). Violin plots show marginal distributions (gray), and median values (gray lines); sample sizes are indicated along the abscissa. Only waveforms with a dominant trough followed by a dominant peak contributed to the analysis of waveform features. We also provide results for only the shortest latency optotagged neurons separately (<5 ms; see Supplementary Fig. S11). Statistical comparisons were based on a Wilcoxon rank sum test.

**Fig. 4:**
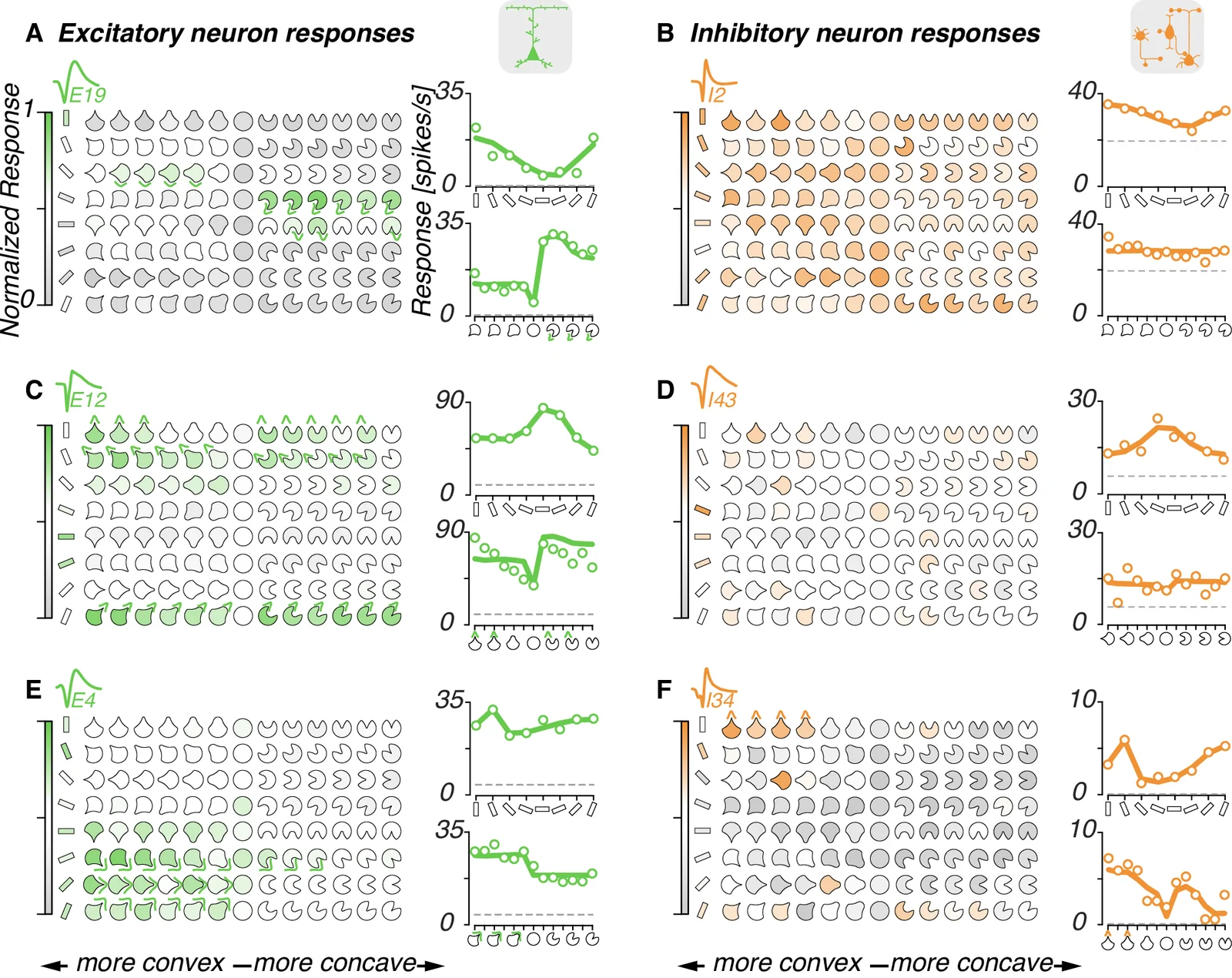
Visual form selectivity of excitatory and inhibitory neurons. Responses of example excitatory (green) and inhibitory (orange) neurons to bar and shape stimuli. Each stimulus is color coded based on the neuron’s normalized response to that stimulus (see color bar; more saturated colors indicate higher responses; white indicates the average response). **A** Left: Data from an example excitatory neuron showing robust, selective responses to only a subset of bar and shape stimuli (hand-draw features in green show the preferred contour segment identified by model fitting). Top right: Neuronal selectivity for bar orientation (line shows model fit; dashed gray line shows spontaneous activity). Bottom right: Neuronal selectivity for contour shape (line shows model fit; responses to shapes at the optimal stimulus rotation are shown). The model fits provided a good account of the observed responses, confirming that this excitatory neuron was selective for bar orientation and contour shape (hand-draw features in green show the preferred contour segment identified by model fitting; also see Supplementary Fig. S6). **B** Left: Data from an example inhibitory neuron showing strong and selective responses to bar stimuli, but no systematic responses to shape stimuli. Top right: Neuronal selectivity for bar orientation (same format as in **A**). Bottom right: Neuronal selectivity for contour shape. The model fits provided a good account of the observed responses, confirming that this inhibitory neuron was selective for bar orientation but unselective for contour shape (also see Supplementary Fig. S7). **C**–**F** Responses of additional example neurons, both excitatory and inhibitory (same format as in **A**, **B**). Only one inhibitory neuron in our dataset (responses shown in **F**) was selective for contour shape.

**Fig. 5:**
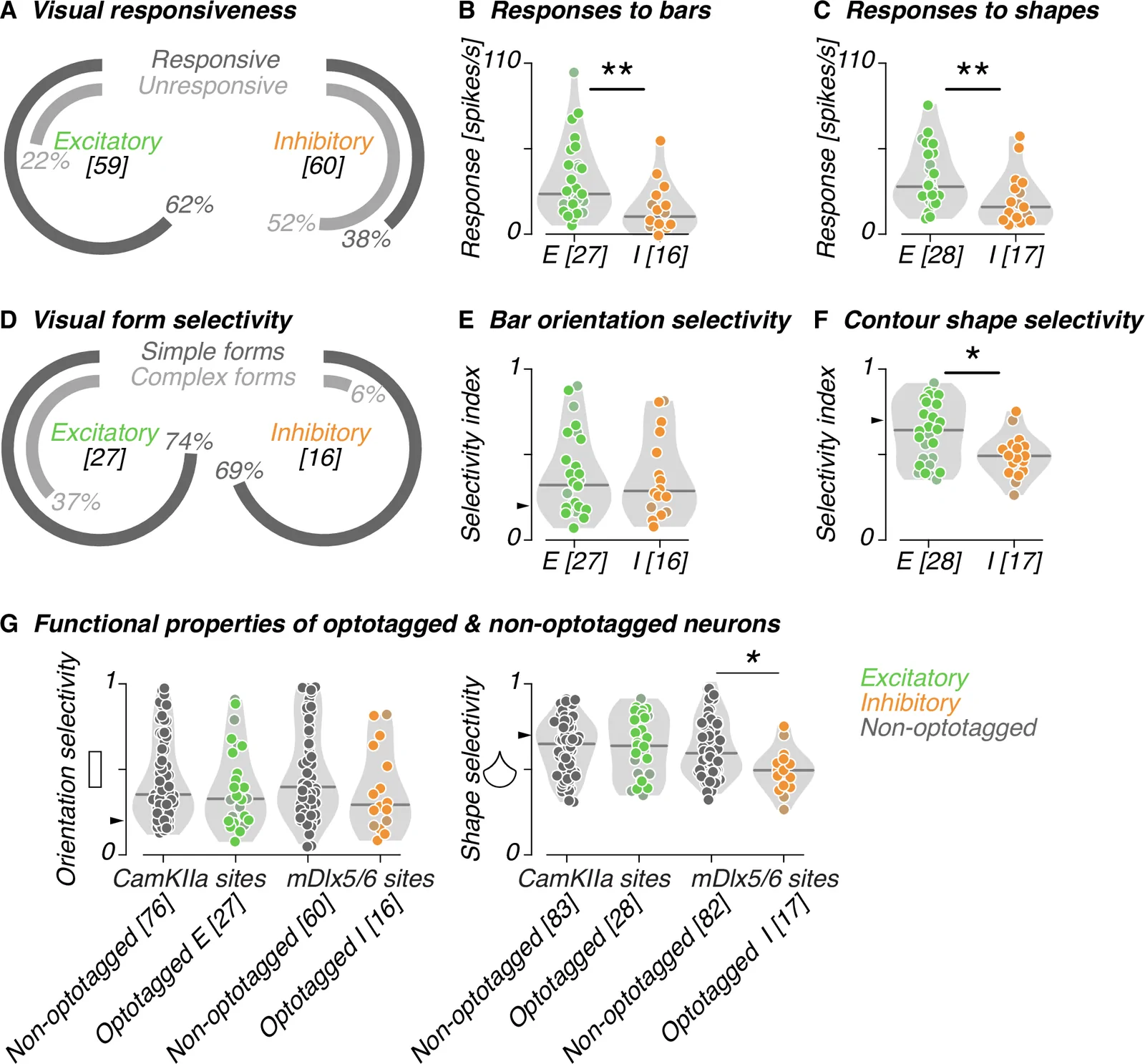
Responses of excitatory and inhibitory neurons to visual stimulation. **A** Proportion of visually responsive and unresponsive optotagged neurons (dark and light gray; some neurons were untested). Sample sizes for excitatory and inhibitory neurons are indicated (green and orange). **B** Distribution of peak responses to bar stimuli (*p* ≤ 0.01; data are color-coded by neuronal cell type and activation latency). **C** Distribution of peak responses to shape stimuli (*p* ≤ 0.01). **D** Proportion of visually responsive optotagged neurons that are selective for simple forms (i.e., bar orientation) and complex forms (i.e., contour shape; same format as in **A**). **E** Distribution of orientation selectivity indices (arrowhead shows criterion for bar orientation selectivity; index ≥0.2). **F** Distribution of shape selectivity indices (*p* ≤ 0.05; arrowhead shows criterion for contour shape selectivity; index ≥0.7). **G** Functional properties of optotagged and non-optotagged neurons. We recorded a collection of non-optotagged neurons (*N* = 165; gray) in each of three macaques, at the same sites in which we recorded optotagged neurons (Supplementary Table S2; also see Supplementary Fig. S9). Left: Compared to non-optotagged neurons (gray), excitatory and inhibitory neurons had similar selectivity for simple forms. Right: Compared to non-optotagged neurons, excitatory neurons had similar selectivity for complex forms, but inhibitory neurons had lower selectivity (*p* ≤ 0.05). Violin plots in **B**, **C** and **E**–**G** show marginal distributions (gray), and median values (gray lines); sample sizes are indicated along the abscissa. Statistical comparisons were based on a Wilcoxon rank sum test.

**Fig. 6:**
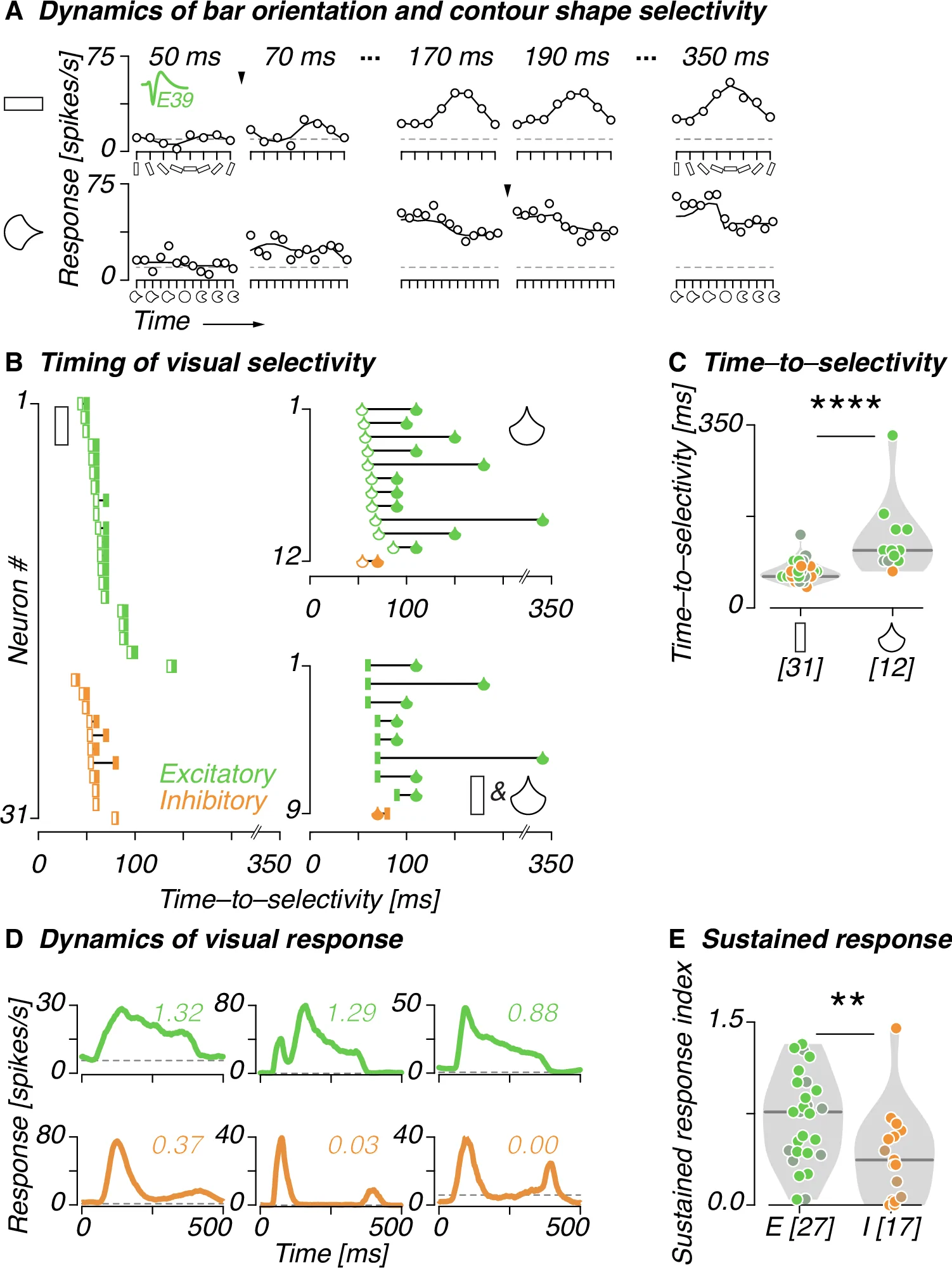
Dynamics of visual form selectivity in excitatory and inhibitory neurons. **A** Responses of an excitatory neuron, calculated over different epochs. Responses to bar and shape stimuli are shown separately (top and bottom; waveform is shown in green). The neuron’s visual latency was ~50 ms and the stimulus duration was 300 ms. Time–to–selectivity for bar orientation and contour shape are marked (see arrowheads; 60 ms and 180 ms). **B** We examined the dynamics of visual form selectivity in optotagged neurons that were selective for bar orientation, contour shape, or both (*N* = 31, 12, 9, respectively). Measures of visual latency (open symbols) and time–to–selectivity (filled symbols) are shown for each optotagged neuron. Data for excitatory and inhibitory neurons are shown separately (green and orange), and are sorted by increasing visual latency. Left: Data for neurons selective for bar orientation. Top right: Data for  neurons selective for contour shape. Bottom right: Within-neuron comparisons of time–to–selectivity measures for bar orientation and contour shape for neurons selective for both types of visual forms. **C** Distribution of time–to–selectivity measures for bar orientation and contour shape across all visually selective optotagged neurons (*p* ≤ 0.0001; data are color-coded by neuronal cell type and activation latency). We also provide results for only the shortest latency optotagged neurons (<5 ms; see Supplementary Fig. S12). **D** Example PSTHs of excitatory and inhibitory neurons. Many excitatory neurons had a late sustained response phase whereas many inhibitory neurons did not. The sustained response index for each neuron is listed above its PSTH. **E** Distribution of sustained response index for excitatory and inhibitory neurons. Excitatory neurons had higher sustained response indices than inhibitory neurons (*p* ≤ 0.01). Violin plots in **C**, **E** show marginal distributions (gray), and median values (gray lines); sample sizes are indicated along the abscissa. Statistical comparisons were based on a Wilcoxon rank sum test.

**Fig. 7:**
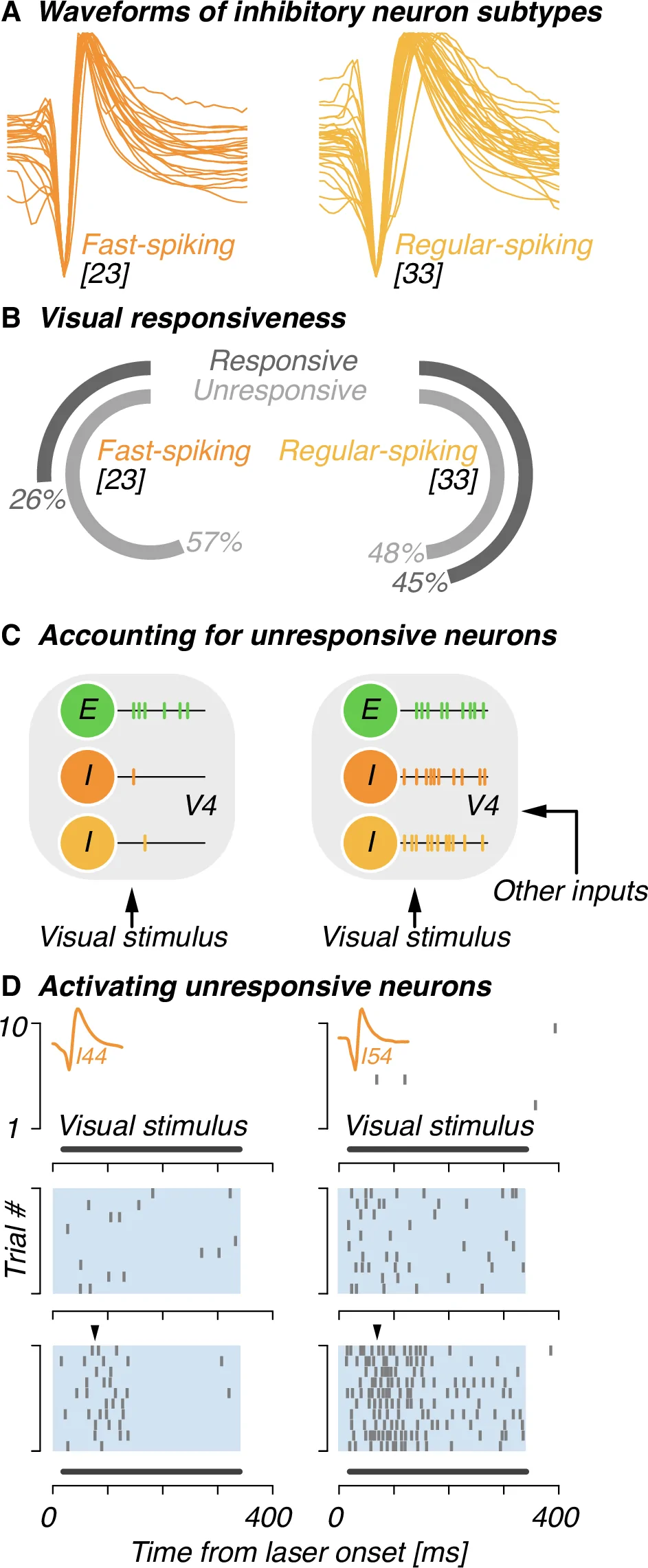
Visual responsiveness of subtypes of inhibitory neurons. **A** Extracellular waveforms of fast-spiking and regular-spiking inhibitory neurons (dark and light orange). Waveforms with trough–to–peak values of <0.2 ms were classified as fast-spiking; sample sizes for each subtype are indicated. **B** Proportion of visually responsive and unresponsive neurons among fast-spiking and regular-spiking inhibitory neurons (dark and light gray; some neurons were untested). **C** Schematic outlining a hypothesis for why some V4 inhibitory neurons may be visually unresponsive: these neurons may not respond to visual stimulation alone (left), and may require a coincidence of inputs to become visually responsive (right). **D** Data from two experimental sessions in which we tested the hypothesis in **C** for two fast-spiking inhibitory neurons (columns; waveforms are shown; see text for details). Black lines along abscissa show the visual stimulation period; blue shaded regions show the optical stimulation period; arrowheads show the average visual response latency of other V4 inhibitory neurons that were visually responsive.

We deployed the optotagging approach in macaques to interrogate excitatory and inhibitory neurons in cortical area V4, a cornerstone of the visual form processing pathway in primates (Fig. 1). In what follows, we highlight functional signatures of the two neuronal cell types uncovered by this work. We first describe how excitatory and inhibitory neurons respond to optical stimulation, establishing a robust methodological pipeline for optotagging these neuronal cell types in the macaque cerebral cortex (Figs. 2, 3). We then describe how excitatory and inhibitory neurons respond to visual stimulation, demonstrating their specialized roles in form vision (Figs. 4–6). And we end by exploring functional differences between subtypes of inhibitory neurons, identifying key directions for future experiments (Fig. 7).

### Rationale for data collection strategy

Excitatory neurons constitute ~80% of all cortical neurons in macaques, whereas inhibitory neurons are only ~20%^45,46^. As such, traditional neurophysiological studies of the macaque visual cortex are likely to have oversampled excitatory neurons due to their prevalence. Given these proportional differences, it stands to reason that we can already make judicious guesses about the functional properties of V4 excitatory neurons based on prior work. In contrast, we know little about the functional properties of V4 inhibitory neurons. To elucidate cell type-specific mechanisms of high-level vision, we deliberately tailored our experimental approach in three ways. First, to gain insights into an understudied neuronal cell type, we focused the majority of our data collection efforts on sampling optotagged inhibitory neurons in each of two macaques. Second, to facilitate direct functional comparisons between neuronal cell types, we also sampled optotagged excitatory neurons in a third macaque. We carefully matched the datasets for excitatory and inhibitory neurons in terms of size, cortical extent and receptive field eccentricity. Third, to link our findings to previous work, we also sampled a *generalist* population of V4 neurons, recorded agnostically without optotagging, in all three macaques. We further confirmed that the generalist neuronal population had functional properties that were consistent with prior studies^3,11,47^ and similar across all three macaques. The functional similarities across animals demonstrate that the visual response properties central to the current study are not susceptible to differences across individual animals. This three-pronged approach provided a solid foundation for asking how the responses of inhibitory neurons differed from those of excitatory neurons, from the generalist excitatory-dominated population, and from expectations based on prior work.

### Excitatory and inhibitory neurons differ in their responses to optical stimulation

We characterized the electrophysiological responses of excitatory and inhibitory neurons, identified by optotagging, using various patterns of blue light illumination (450 nm). Optotagged neurons were sampled across a large cortical extent, and across many electrode penetrations, thereby increasing the likelihood of surveying different functional domains within V4 (Supplementary Table S2). The optical stimulation conditions included both sinusoidal and square wave modulation (Fig. 2A, B; see *Methods* for rationale). An example excitatory neuron showed robust, short-latency responses across all laser modulation frequencies tested (Fig. 2A; activation latency was 2.9 ms). An example inhibitory neuron showed similarly robust, short-latency responses to all but the highest modulation frequencies tested (Fig. 2B; activation latency was 2.5 ms). Both example neurons were capable of following laser modulation frequencies up to 32–64 Hz. These data demonstrate the feasibility of our optotagging approach, and its utility for identifying the spiking responses of individual excitatory and inhibitory neurons in the macaque cerebral cortex.

We compared the electrophysiological response properties of all optotagged excitatory and inhibitory neurons (59 E; 60 I). Blue light illumination elicited short-latency responses in both neuronal cell types (Fig. 2C**;** median was 3.3 ms and 2.5 ms for E and I). Sinusoidal and square wave laser conditions yielded similar activation latencies (Supplementary Fig. S3A, B; latencies for the two conditions were highly correlated; *r* = 0.87, *p* < 0.0001 for E; *r* = 0.71, *p* < 0.0001 for I). For each optotagged neuron, we took the shortest latency value across all laser conditions as the activation latency. Note that excitatory neurons with long activation latencies (>5 ms) may represent cases of indirect activation via neighboring neurons, whereas inhibitory neurons with long activation latencies do not. Given the paucity of studies characterizing the responses of optotagged excitatory and inhibitory neurons in the macaque cerebral cortex, we have elected to include data from all neurons in this study, regardless of their activation latency. However, to ensure the accuracy of our findings, we have conducted all quantitative comparisons between excitatory and inhibitory neurons in two ways: (i) including all optotagged neurons, and (ii) including only optotagged neurons with short activation latencies (<5 ms). In all cases, statistical comparisons of excitatory and inhibitory neuron properties yielded similar results with and without this short latency criterion. We therefore report results based on the inclusion of all optotagged neurons, and we represent data from neurons with short and long activation latencies separately (brighter and darker shades). We also provide results for only the short-latency optotagged neurons separately (Supplementary Figs. S11–S12), and we report all statistical comparisons between excitatory and inhibitory neurons for each of the two latency criteria.

To assess the robustness of our optogenetic approach, we quantified the responsiveness of optotagged neurons across optical stimulation conditions. Inhibitory neurons showed more diverse response patterns than excitatory neurons (see Supplementary Fig. S4 for additional examples of inhibitory neuron responses). Across the population, we found that optical stimulation elicited vigorous responses in both neuronal cell types, but the peak responses of excitatory neurons were lower than those of inhibitory neurons, consistent with the known biophysical properties of the two classes of cortical neurons^48^ (Fig. 2D**;** for all neurons, *p* < 0.0001; median was 61 spikes/sec and 121 spikes/sec for E and I; for short-latency neurons, *p* < 0.01; median was 84 spikes/sec and 142 spikes/sec for E and I). Sinusoidal and square wave laser conditions elicited similar peak responses (see Supplementary Fig. S3C). Spontaneous responses derived from trials without optical stimulation were equally low for the two neuronal cell types, consistent with reports of low spontaneous activity in cortical area V4^49^ (for all neurons, mean was 2 spikes/sec and 3 spikes/sec for E and I; for short-latency neurons, mean was 2 spikes/sec and 3 spikes/sec for E and I). These findings demonstrate that selective optogenetic perturbations of excitatory and inhibitory neurons can have robust effects on cortical activity in macaques.

To assess the sensitivity of excitatory and inhibitory neurons to time-varying patterns of optical stimulation, we studied the responses of all optotagged neurons across the sinusoidal modulation frequencies tested. Excitatory neurons followed higher laser modulation frequencies than inhibitory neurons. The preferred frequency, corresponding to the peak response, was higher for excitatory neurons than inhibitory neurons (Fig. 2E**;** for all neurons, *p* < 0.0001; median was 17 Hz and 9 Hz for E and I; for short-latency neurons, *p* < 0.0001; median was 22 Hz and 9 Hz for E and I). Similarly, the high-frequency cutoff, corresponding to the highest frequency eliciting half of the peak response, was also higher for excitatory neurons than inhibitory neurons (for all neurons, *p* < 0.0001; median was 99 Hz and 68 Hz for E and I; for short-latency neurons, *p* < 0.0001; median was 116 Hz and 68 Hz for E and I). These findings suggest that future experiments seeking to manipulate excitatory and inhibitory neuron activity in macaques may benefit from using different patterns of optical stimulation for each neuronal cell type.

To provide broader insights into the properties of excitatory and inhibitory neurons in macaques, we leveraged the optotagging approach to study the relationship between the genetic identities of cortical neurons and the properties of their extracellularly recorded spike waveforms. Waveform features have been previously used in monkeys to classify cortical neurons into putative excitatory or inhibitory neuronal cell classes^32–34^, but this relationship is only correlative and our optotagging approach is ideally suited to test it. We therefore compared the spike waveforms of all optotagged excitatory and inhibitory neurons (Fig. 3A; thick lines are average waveforms; see Supplementary Fig. S5 for individual waveforms of all optotagged neurons). Despite considerable overlap, the average waveform of excitatory neurons was broader than that of inhibitory neurons (for all neurons, mean was 0.29 ms and 0.23 ms for E and I; for short-latency neurons, mean was 0.32 ms and 0.23 ms for E and I). To assess the waveform properties of optotagged neurons quantitatively, we calculated two waveform features: (i) the trough–to–peak time, and (ii) the repolarization time (Fig. 3B, see left inset). When examining the two waveform features jointly, we found that excitatory and inhibitory neuron waveforms were weakly clustered (Fig. 3B; ellipses represent the output of a k-means clustering procedure; subplots on right show data for short latency neurons only). The centroids of excitatory and inhibitory neuron clusters were determined to be significantly different based on a shuffle analysis (*p* < 0.001; see *Methods*). On average, excitatory neurons had broader extracellular waveforms than inhibitory neurons, with longer trough–to–peak times (Fig. 3C, left; for all neurons, *p* < 0.01; median was 0.27 ms and 0.21 ms for E and I; for short-latency neurons, *p* < 0.001; median was 0.30 ms and 0.22 ms for E and I). Excitatory neurons also had longer repolarization times (Fig. 3C, right; for all neurons, *p* < 0.001; median was 0.10 ms and 0.07 ms for E and I; for short-latency neurons, *p* < 0.001; median was 0.13 ms and 0.07 ms for E and I). Additionally, a linear classifier could predict neuronal cell type based on waveform features with an average performance accuracy of 63% (see *Methods*). These findings confirm that extracellular waveform features are useful for surveying putative excitatory and inhibitory neurons in the macaque cerebral cortex, but classification errors are to be expected, and studies relying on cell type-specificity will benefit from the higher precision of the optotagging approach.

### Excitatory and inhibitory neurons differ in their responses to visual stimulation

We characterized the visual responses of excitatory and inhibitory V4 neurons using two classes of form stimuli: simple and complex. The simple forms were bar stimuli used to assess orientation selectivity, a property of neurons in early visual cortical areas. The complex forms were shape stimuli used to assess contour shape selectivity, a property of neurons in higher visual cortical areas, including V4. A major advantage of the shape stimuli used in this study is that they contain systematic variations in contour curvature, enabling us to characterize the preferences of V4 neurons for curved contour segments at specific positions relative to object center^3,11^. An example excitatory neuron showed selective responses to the two stimulus classes, responding systematically to only a subset of stimuli within each class (Fig. 4A, left). This excitatory neuron preferred a vertical bar and a shape resembling a fortune cookie pointing downward and leftward (hand-drawn contour features show the preferred contour segment identified by model fitting). An example inhibitory neuron showed selective responses to bar stimuli, but no systematic responses to shape stimuli (Fig. 4B, left). This inhibitory neuron preferred a near-vertical bar but no specific subset of shape stimuli. Optotagged neurons showed various preferences for bar orientation and contour shape (see Fig. 4C–F for additional example responses).

To quantify the visual form selectivity of optotagged neurons, we fit their responses using well-established models of neuronal selectivity for bar orientation and contour shape (see *Methods*). The example excitatory neuron showed responses that were moderately selective for bar orientation (Fig. 4A, top right; solid line shows model fit; dashed line shows spontaneous activity). The same excitatory neuron showed responses that were selective for contour shape, as visualized at the optimal stimulus rotation (Fig. 4A, bottom right; line shows model fit; hand-drawn contour features show the preferred contour segment identified by model fitting; also see Supplementary Fig. S6 for responses and model fits across all stimulus rotations). In contrast, the example inhibitory neuron showed responses that were moderately selective for bar orientation (Fig. 4B, top right) but unselective for contour shape (Fig. 4B, bottom right; also see Supplementary Fig. S7 for responses and model fits across all stimulus rotations). These data demonstrate the feasibility of driving selective responses from V4 excitatory and inhibitory neurons using the two classes of visual form stimuli.

We compared the visual response properties of all optotagged excitatory and inhibitory neurons, revealing key functional differences between them. Based on preliminary tests with standard visual stimuli used in many V4 studies (e.g., bars, spots, sinusoidal gratings and two-dimensional shapes), we found that most excitatory neurons were visually responsive, whereas far fewer inhibitory neurons were responsive (Fig. 5A; 62% and 38% for E and I; see *Methods* for details). Following these preliminary tests, we proceeded to characterize all visually responsive optotagged neurons in quantitative experiments. Consistent with the preliminary tests, excitatory neurons responded more vigorously to both stimulus classes than inhibitory neurons. Excitatory neurons had higher peak responses to bar stimuli (Fig. 5B; for all neurons, *p* < 0.01; median was 26 spikes/sec and 11 spikes/sec for E and I; for short-latency neurons, *p* < 0.05; median was 30 spikes/sec and 9 spikes/sec for E and I). Excitatory neurons also had higher peak responses to shape stimuli (Fig. 5C; for all neurons, *p* < 0.01; median was 31 spikes/sec and 17 spikes/sec for E and I; for short-latency neurons, *p* < 0.01; median was 28 spikes/sec and 16 spikes/sec for E and I). In contrast, spontaneous responses derived from trials without visual stimulation were equally low for both neuronal cell types (for all neurons, median was 1 spike/sec and 6 spikes/sec for E and I; for short-latency neurons, median was 2 spikes/sec and 5 spikes/sec for E and I). These findings point to clear differences in the visual responsiveness of V4 excitatory and inhibitory neurons.

To facilitate comparisons of visual form selectivity across neuronal cell types, we fit the responses of each optotagged neuron using well-established quantitative models of bar orientation selectivity and contour shape selectivity (see *Methods* for details). The model we used for quantifying shape selectivity captured how well each neuron’s responses could be accounted for in terms of a preference for a curved contour feature at a specific position relative to object center^3^. Based on these model fits, we computed two selectivity indices for neuronal classification. Neurons with bar orientation selectivity index ≥0.2 were classified as selective for bar orientation, indicating that they signaled simple visual forms. We chose this criterion for orientation selectivity based on prior studies in early visual cortex. Neurons with contour shape selectivity index ≥0.7 were classified as selective for contour shape, indicating that they signaled complex visual forms. We chose this criterion for shape selectivity based on prior studies in V4^3,11^—a stringent criterion ensuring unambiguous classification of neurons. We found that an equal proportion of excitatory and inhibitory neurons were selective for simple visual forms (Fig. 5D; for all neurons, 74% and 69% for E and I; for short-latency neurons, 71% and 77% for E and I). In contrast, a substantially larger proportion of excitatory neurons were selective to complex visual forms (Fig. 5D; for all neurons, 37% and 6% for E and I; for short-latency neurons, 47% and 8% for E and I). In fact, only one inhibitory neuron was classified as selective for contour shape (see Fig. 4F). Given the known, imperfect targeting specificity of AAVs containing the mDlx5/6 enhancer, it is conceivable that this shape-selective neuron was not inhibitory. The responses of all other inhibitory neurons could not be well explained in terms of selectivity for contour shape information. Across the population of optotagged neurons, we found that excitatory and inhibitory neurons were equally selective for bar orientation information (Fig. 5E; for all neurons, median selectivity index was 0.33 and 0.29 for E and I; for short-latency neurons, median selectivity index was 0.34 and 0.31 for E and I; arrowhead shows criterion for selectivity). In contrast, excitatory neurons were more selective for contour shape information (Fig. 5F; for all neurons, *p* < 0.05; median selectivity index was 0.64 and 0.49 for E and I; for short-latency neurons, *p* < 0.01; median selectivity index was 0.70 and 0.49 for E and I; arrowhead shows criterion for selectivity). Within-neuron comparisons of the two selectivity indices showed that some neurons were selective for bar orientation, contour shape or both, but there was no correlation between the two selectivity measures (Supplementary Fig. S8A). To ensure that our observation of few shape-selective inhibitory neurons did not depend on the criterion level used to classify neurons, we tested a range of criterion values of the shape selectivity index (Supplementary Fig. S8B; see *Methods* for details). At each criterion level tested, we re-evaluated the percentage of shape-selective neurons that were excitatory or inhibitory. Across criterion levels, we found that the large majority of neurons classified as shape-selective were excitatory. These findings demonstrate that strong selectivity for contour shape information was primarily restricted to V4 excitatory neurons.

To corroborate our observation of higher shape selectivity in excitatory neurons, we conducted three supporting analyses. First, to evaluate the statistical significance of our shape selectivity index, we conducted a shuffle analysis on this measure. The shuffle analysis determined the probability that each neuron’s observed index was larger than expected by random sampling. This analysis confirmed that the shape selectivity indices for many optotagged neurons were statistically significant (Supplementary Fig. S8C; for all neurons, *p* < 0.05; 67% and 58% for E and I; median selectivity index was 0.64 and 0.49 for E and I; for short-latency neurons, *p* < 0.005; 83% and 64% for E and I; median selectivity index was 0.70 and 0.49 for E and I). When we only included neurons with statistically significant indices, we found a larger difference in shape selectivity between excitatory and inhibitory neurons (Supplementary Fig. S8C; for all neurons, *p* < 0.0001; median was 0.74 and 0.54 for E and I; for short-latency neurons, *p* < 0.0001; median was 0.74 and 0.54 for E and I). Second, to confirm the robustness of our shape selectivity index, we conducted a cross-validation analysis on this measure. This analysis confirmed that excitatory neurons were more selective for contour shape information than inhibitory neurons, and that this difference in shape selectivity held for both train and test data (Supplementary Fig. S8D**;** for all neurons, *p* < 0.05; for train data, median selectivity index was 0.66 and 0.52 for E and I; for test data, the median selectivity index was 0.48 and 0.16 for E and I; for short-latency neurons, *p* < 0.01; for train data, median selectivity index was 0.72 and 0.52 for E and I; for test data, the median selectivity index was 0.58 and 0.20 for E and I). Third, to further validate insights from our model-driven measure of shape selectivity, we also evaluated an alternative, data-driven measure of shape selectivity calculated directly from neuronal responses at the optimal stimulus rotation. This analysis also demonstrated that excitatory neurons were more shape-selective than inhibitory neurons (Supplementary Fig. S8E; for all neurons, *p* < 0.05; the median data-driven selectivity index was 1.77 and 1.10 for E and I; for short-latency neurons, *p* > 0.05; the median data-driven selectivity index was 1.52 and 1.02 for E and I). Collectively, the results of these supporting analyses bolster our finding that excitatory neurons are more selective for complex visual forms than inhibitory neurons.

### Comparing the properties of excitatory and inhibitory neurons to those of non-optotagged neurons

It is possible that our observation of weak shape selectivity in inhibitory neurons could have arisen incidentally by confining recordings of optotagged inhibitory neurons to V4 regions lacking shape-selective neurons. To evaluate this possibility directly, we asked how the functional properties of excitatory and inhibitory neurons identified by optotagging compared to those of a generalist population of V4 neurons nearby. We therefore recorded a collection of neurons without knowledge of their cell type identity (*N* = 165), herein referred to as *non-optotagged* neurons, sampled within the same cortical sites where we recorded optotagged neurons (Supplementary Table S2; see *Methods* for details). We first confirmed that non-optotagged neurons had similar functional properties across animals, demonstrating the lack of a functional bias across cortical sites and subjects (Supplementary Fig. S9A). Having established this functional similarity, we then proceeded to compare the functional properties of optotagged neurons to those of non-optotagged neurons. Excitatory and inhibitory neurons had similar selectivity for bar orientation compared to non-optotagged neurons (Fig. 5G, left). Excitatory neurons also had similar selectivity for contour shape compared to non-optotagged neurons (Fig. 5G, right; arrowhead shows criterion for selectivity). In contrast, inhibitory neurons had lower selectivity for contour shape compared to non-optotagged neurons (Fig. 5G, right; *p* < 0.05; median selectivity index was 0.49 for all I, 0.49 for short-latency I, and 0.56 for non-optotagged neurons). These observations demonstrate that V4 inhibitory neurons are clear outliers in terms of their weak shape selectivity.

We also asked whether the functional properties of optotagged and non-optotagged neurons varied across cortical depth. While our single-electrode recording approach is not ideally suited for localizing neurons in specific cortical layers, we reasoned that a sensible proxy would be to examine the relative cortical depth of all recorded neurons, calculated based on their distance from brain surface. We found that excitatory and inhibitory neurons were recorded across a similar range of cortical depths (Supplementary Fig. S9B); for both neuronal cell types, the distributions of relative cortical depth were not different from uniform (*p* > 0.05 based on chi-square goodness of fit test of normality). These findings demonstrate that the laminar profiles of excitatory and inhibitory neurons sampled in the study were qualitatively similar, consistent with the comparable laminar transduction patterns of the two AAVs used (see Fig. 1C, D). We also observed similar functional properties of neurons across cortical depth. Specifically, we found neurons that were selective for simple and complex forms, as well as neurons that were visually responsive and unresponsive at various cortical depths (Supplementary Fig. S9C). For both neuronal cell types, the distributions of functional properties across relative cortical depth were not different from uniform (*p* > 0.05 based on chi-square goodness of fit test of normality). These findings demonstrate that the functional properties of V4 neurons evaluated in our study were not strongly restricted to specific laminar compartments, consistent with a previous study^50^.

### Excitatory and inhibitory neurons differ in their response dynamics

We wondered whether the preferences of V4 neurons for simple and complex visual forms were inherited from afferent inputs or generated de novo within V4. We therefore examined the dynamics of visual form selectivity in neurons that were selective for bar orientation, contour shape or both. We calculated the time it took for each neuron’s responses to reach criterion levels of visual form selectivity, herein referred to as time–to–selectivity (see *Methods*). Data from an example excitatory neuron show the temporal evolution of responses to bar and shape stimuli (Fig. 6A, top and bottom; solid line shows model fit; dashed line shows spontaneous activity; responses to shape stimuli are shown at the optimal stimulus rotation). Selectivity for bar orientation reached criterion levels fairly early (arrowhead shows time–to–selectivity was 60 ms). In contrast, selectivity for contour shape reached criterion levels substantially later in time (arrowhead shows time–to–selectivity was 180 ms).

To determine when V4 neurons signaled information about bar orientation and contour shape, we examined the time–to–selectivity measures for each optotagged neuron. Additionally, we compared these time–to–selectivity measures to the time it took for visual information to reach each V4 neuron, herein referred to as visual response latency. The visual response latencies of optotagged neurons were around 60 ms, consistent with previous work^51^. Inhibitory neurons had slightly earlier visual response latencies than excitatory neurons, but the difference was not statistically significant (for all neurons, median visual latency was 55 ms and range was 37–79 ms for I and median visual latency was 61 ms and range was 40–136 ms for E; for short-latency neurons, median visual latency was 55 ms and range was 37–79 ms for I and median visual latency was 60 ms and range was 40–87 ms for E). Across the population, we found that excitatory and inhibitory neurons were selective for bar orientation shortly after their visual response latencies (Fig. 6B, left; open symbols show visual latency; filled symbols show time–to–selectivity). In contrast, neurons were selective for contour shape much later in time, suggesting a more protracted processing time for complex forms (Fig. 6B, top right; open symbols show visual latency; filled symbols show time–to–selectivity). Additionally, for the subset of neurons that were selective for both bar orientation and contour shape, within-neuron comparisons confirmed that bar orientation selectivity developed earlier than contour shape selectivity (Fig. 6B, bottom right; bar and shape symbols show time–to–selectivity for each stimulus class). Across all visually responsive neurons, bar orientation selectivity developed earlier than contour shape selectivity (Fig. 6c; for all neurons, *p* < 0.0001; median was 60 ms and 110 ms for bar orientation and contour shape; for short-latency neurons, *p* < 0.0001; median was 60 ms and 110 ms for bar orientation and contour shape). By visualizing the entire time course of visual form selectivity for individual neurons, we confirmed that selectivity for bar orientation was typically highest in early response epochs whereas selectivity for contour shape was highest in later response epochs (Supplementary Fig. S13A–B). To ensure that these findings did not depend on how we quantified time–to–selectivity, we repeated the analyses using a different measure (see *Methods*) and obtained qualitatively similar results (Supplementary Fig. S13C; for all neurons, *p* < 0.0001; median was 70 ms and 120 ms for bar orientation and contour shape). These findings suggest that information about bar orientation may already be present in the afferent inputs to V4 neurons, whereas information about contour shape may be computed gradually over time, and primarily in excitatory neurons. The gradual synthesis of shape information over time is consistent with previous studies^5,52^.

In addition to the differences in visual selectivity dynamics described, we also observed clear differences in the visual response dynamics of the two neuronal cell types. The peri-stimulus time histograms (PSTHs) of many excitatory neurons had a characteristic, late and sustained response phase whereas many inhibitory neurons lacked this property (Fig. 6D). We quantified the sustained response dynamics using a modulation index and examined this measure across the two neuronal cell types. We found that across the population of optotagged neurons, excitatory neurons had higher sustained response indices than inhibitory neurons (Fig. 6E; for all neurons, *p* < 0.01; median index was 0.76 and 0.37 for E and I; for short-latency neurons, *p* < 0.05; median index was 0.76 and 0.46 for E and I). This finding suggests that excitatory neurons may receive different afferent inputs over time, possibly reflecting recurrent or feedback processing, which may in turn gradually boost their shape selectivity.

### Visual responsiveness of inhibitory neuron subtypes

More than half of V4 inhibitory neurons we recorded were visually unresponsive, an unexpected finding that merits further investigation. Given the known molecular diversity of cortical inhibitory neurons, it is possible that inhibitory neuron subtypes differ in their visual responsiveness^53^. The AAV vector we used targeted most inhibitory neuron subtypes indiscriminately (but see ref. ^29^), preventing us from classifying their electrophysiological signatures with confidence. Nevertheless, we wondered whether we could discern any functional differences among putative subtypes of inhibitory neurons. Following previous studies, we classified optotagged inhibitory neurons into two functional classes, fast-spiking and regular-spiking, based on the width of their extracellular waveforms^54–56^. Waveform-based classification of subtypes of inhibitory neurons is an imperfect approach that may not map precisely onto molecularly distinct cell types. Nevertheless, we leverage this approach here as a useful starting point for surveying potential functional differences between inhibitory neuron subtypes in macaques. Neurons with trough–to–peak times <0.2 ms were classified as fast-spiking, and neurons with longer trough–to–peak times were classified as regular-spiking (Fig. 7A; median trough–to–peak times were 0.15 ms and 0.27 ms for fast-spiking and regular-spiking). Based on this functional classification, we found that subtypes of inhibitory neurons did indeed differ in their visual responsiveness. Only a small proportion of fast-spiking neurons were visually responsive and most were unresponsive (Fig. 7B; 26% responsive and 57% unresponsive). In contrast, a roughly equal proportion of regular-spiking neurons were visually responsive and unresponsive (Fig. 7B; 45% responsive and 48% unresponsive). These observations motivate future studies aimed at exploring the role of V4 inhibitory neuron subtypes in visual information processing.

Why are so many V4 inhibitory neurons unresponsive to visual stimuli? It is possible that feedforward visual inputs alone cannot drive robust responses in these inhibitory neurons. Instead, unresponsive inhibitory neurons may require a coincidence of inputs to become visually responsive (Fig. 7C). To test this possibility experimentally, we used optical stimulation to gently depolarize unresponsive inhibitory neurons, thereby mimicking the effects of additional coincident inputs, and we asked whether these neurons became responsive to visual stimulation coincident with optical stimulation. Data from two fast-spiking inhibitory neurons demonstrate the absence of robust responses to visual stimulation alone (Fig. 7D, top row; black line shows stimulus presentation period). We chose a level of optical stimulation that elicited low to modest responses from each neuron when presented alone (Fig. 7D, middle row; blue box shows optical stimulation period). We then combined the ineffective visual stimulation with low-level optical stimulation. Surprisingly, we found that the combined stimulation condition produced stronger responses that far exceeded the sum of responses to visual and optical stimulation alone (Fig. 7D, bottom row). In fact, the largest increase in activity in the combined stimulation condition occurred near the expected visual response latencies of inhibitory neurons (Fig. 7D, bottom row; arrowheads indicate the average visual response latency for other visually responsive V4 inhibitory neurons; 55 ms). While our data using combined optical and visual stimulation are limited in extent, these tantalizing findings suggest that the visual responses of some V4 inhibitory neurons may be gated by a coincidence of inputs.

## Discussion

The expansion of the cerebral cortex is a defining evolutionary feature of primates, and a major substrate for their higher visual functions. A prime example is the perception of complex visual shapes—an essential function that is highly elaborated in primates. To gain a deeper mechanistic understanding of the cells and circuits underlying this high-level visual function, we developed and applied an optotagging approach for monitoring the responses of bona fide excitatory and inhibitory neurons in the macaque cerebral cortex. This approach facilitated detailed functional characterization of the two neuronal cell types, revealing key distinctions between them. Below, we highlight the major conceptual and technical advances achieved in this work.

### Conceptual advances

Our study provides critical insights into the cell type-specific mechanisms underlying high-level vision in primates. Leveraging the optotagging approach, we identified several differences in the visual response properties of excitatory and inhibitory neurons in cortical area V4. First, excitatory neurons were more visually responsive than inhibitory neurons, and a large proportion of inhibitory neurons were unresponsive under the passive viewing conditions of our experiments (Fig. 5A–C). Second, excitatory and inhibitory neurons were equally selective for bar orientation, yet excitatory neurons were more selective for contour shape (Fig. 5D–G). These findings point to clear functional specializations in form vision: inhibitory neurons signaled simple visual forms almost exclusively, whereas excitatory neurons signaled both simple and complex visual forms. Third, excitatory neurons developed their selectivity for contour shape significantly later than their selectivity for bar orientation (Fig. 6A–C and Supplementary Fig. S13). Fourth, excitatory neurons had more protracted visual response dynamics than inhibitory neurons, potentially accounting for their stronger shape selectivity (Fig. 6D, E). Collectively, our findings elucidate a unique and dominant role for excitatory neurons in the processing of complex visual shapes. Excitatory neurons may compute contour shape information gradually over time, possibly from orientation-selective inputs as suggested in prior work^52^ and other dynamically changing afferent inputs. In contrast, most inhibitory neurons, which also appear to receive orientation-selective inputs, do not compute contour shape information. The mechanistic insights uncovered in this study are ideally suited to guide refinements in biologically inspired computational models of high-level vision, which currently do not incorporate cell type-specific architectures^18–21^.

Much of what we know about the role of cortical inhibitory neurons in visual information processing comes from studies conducted in other species, thereby motivating comparisons between their findings and ours in macaques. Inhibitory neurons in cortical area V1 of mice, cats and rabbits are reported to be selective for many basic visual properties, albeit more broadly selective than excitatory neurons (e.g., for orientation, spatial frequency, size and contrast)^35–41,57–63^. Inhibitory neurons in V1 of these other species are also reported to be strongly responsive to visual stimuli^48,63–65^. Thus, our finding that inhibitory neurons in cortical area V4 of macaques have weaker contour shape selectivity than excitatory neurons generally agrees with observations made in other species. In contrast, our finding that many inhibitory neurons are not strongly responsive to visual stimuli seems to deviate from observations made in other species. The weaker stimulus-evoked responses of inhibitory neurons need not imply that inhibition in V4 is weak relative to excitation, as this balance depends also on the unknown synaptic strengths and the correlations among inputs to individual neurons. Additionally, as cortical areas appear to operate in a loosely balanced regime^66^, there is some flexibility in the requirements for excitatory/inhibitory balance. The paucity of visually responsive inhibitory neurons in our study suggests that inhibitory neurons in higher areas of the primate cerebral cortex may contribute to visual information processing in fundamentally different ways than do excitatory neurons.

Most of the V4 inhibitory neurons we identified by optotagging were unresponsive to visual stimuli. These unresponsive inhibitory neurons are likely to have been under-sampled in previous V4 studies where visual stimuli were tailored to individual neurons^3,11^. It is possible that some of these inhibitory neurons are hyper-selective for visual features not examined in our study. We tested this possibility by comparing the responses of a small population of inhibitory neurons to a broader array of stimulus classes: our main stimuli (bars and shapes) and new stimuli (colors, complex shapes and real-world objects). In these limited cases, we observed similar peak responses across all stimulus classes, suggesting that V4 inhibitory neurons are unlikely to be hyper-selective, consistent with insights from mouse V1 studies reporting broadly responsive inhibitory neurons^35–41,60,63^. An alternative possibility is that unresponsive inhibitory neurons in V4 typically exist in a hyperpolarized state, becoming responsive to visual stimuli only under specific, permissive conditions. We tested this possibility in only a handful of sessions by gently depolarizing unresponsive inhibitory neurons optogenetically, and we asked whether they became responsive to visual stimuli. In these limited cases, we found that application of low-power optical stimulation coincident with visual stimulation was sufficient to drive stimulus-evoked responses in unresponsive inhibitory neurons (Fig. 7D). Using a waveform-based classification approach, we also found that a larger proportion of unresponsive inhibitory neurons were fast-spiking compared to regular-spiking, pointing to potential functional differences between inhibitory neuron subtypes (Fig. 7B). Given the limited accuracy of the waveform-based classification approach, future studies are needed to confirm such functional differences using AAVs targeting molecularly distinct subtypes of inhibitory neurons. Nevertheless, our current findings, though limited in extent, suggest that unresponsive inhibitory neurons in V4 may require a coincidence of inputs to become visually responsive (Fig. 7C). Our observation of many unresponsive neurons may be related to other observations made in macaques. For example, optogenetic stimulation of neurons in the frontal eye fields only evokes saccades when combined with low-amplitude electrical stimulation^67^; similarly, electrical stimulation of these neurons strongly modulates visual cortical activity only when combined with a visual stimulus^68–70^. Future studies are needed to determine the nature of the coincident inputs and the permissive conditions necessary for activating unresponsive inhibitory neurons in V4.

### Technical advances

Our study establishes a robust methodological pipeline for optotagging excitatory and inhibitory neurons in the cerebral cortex of macaques. Of particular relevance to macaque research, the optotagging approach facilitated direct comparisons of the extracellular waveforms of the two neuronal cell types. The waveforms of excitatory neurons tended to be broader than those of inhibitory neurons, consistent with correlative measurements used in previous studies^32–34^ (Fig. 3). Nevertheless, there was considerable overlap between the waveform features of excitatory and inhibitory neurons, suggesting that the optotagging approach provides superior identification of these neuronal cell types in macaques.

Can optotagging studies in mice obviate the need for similar studies in monkeys? A study employed optotagging approaches in the cerebellum of transgenic mice to learn cell type-specific electrophysiological signatures that can generalize to the cerebellum of macaques^71^. This cross-species approach may be useful for studying neuronal cell types with similar proportions, densities and connectivity patterns across the two species, including the highly stereotypic cell types of the cerebellum. However, the same cross-species approach may be unfeasible for cortical excitatory and inhibitory neurons given known differences in their properties across species and across cortical areas^40,72–74^. Thus, developing optotagging approaches for interrogating excitatory and inhibitory neuronal cell types in macaques, as we have done here, has broad utility for discovering their unique roles in primate brain functions. Furthermore, conducting optotagging studies in different species will enable us to test general principles of cortical cell function across the animal kingdom.

### Limitations of the study

The optotagging strategy we developed and applied in this study is not without limitations. While our use of single recording electrodes enabled clear identification of optotagged neurons with high isolation quality, this approach hindered us from surveying neuronal functional properties at a larger spatial scale. We were unable to compare the properties of excitatory and inhibitory neurons across different cortical layers. We were also unable to probe functional interactions between optotagged neurons and other surrounding neurons, with the exception of one experimental session where we were fortunate to record two well-isolated neurons simultaneously (see Supplementary Fig. S14). Future studies combining cell type-specific optogenetics with large-scale neural recordings across cortical layers are therefore needed to yield such mechanistic insights. In particular, it would be useful to simultaneously record single optotagged neurons and local field potentials, as such datasets would enable valuable cross-species comparisons of cellular and circuit mechanisms between monkeys and mice. Additionally, the weak AAV-mediated transduction of neurons in the granular layer, as observed in this and other studies^27,28,75^, limits the interpretation of optogenetic circuit dissection studies in the macaque cerebral cortex. Specifically, the inability to transduce and identify optotagged neurons in the granular layer constrains our efforts to discern cell type-specific contributions across cortical microcircuits. Future efforts to develop AAVs with layer-specific gene regulatory elements will greatly facilitate the investigation of laminar cortical computations in macaques.

### Future directions

Our study motivates several lines of future investigation, both within and beyond visual cortical area V4. First, it would be valuable to compare the responses of V4 excitatory and inhibitory neurons across a broader array of visual stimuli, and across different behavioral states; such studies may clarify the contribution of visual and other inputs to the responses of each neuronal cell type. Second, it would be valuable to compare the properties of V4 excitatory and inhibitory neurons across different cortical layers; such studies may clarify how the two neuronal cell types contribute to visual processing in specific microcircuits. Third, it would be valuable to probe functional interactions between V4 excitatory and inhibitory neurons, and between optotagged neurons and surrounding neurons; such studies may clarify principles of cortical processing at the population-level. Fourth, it would be valuable to compare the response properties of inhibitory neurons across many visual cortical areas; such studies are necessary for evaluating the specificity and generality of inhibitory neuron function across stages of information processing. Fifth, it would be valuable to expand the optotagging approach to study the functional properties of molecularly distinct inhibitory neurons; such studies will advance a more comprehensive understanding of how inhibitory circuits influence cortical function in macaques.

At its core, our study seeks to reshape how neuroscientists investigate the primate brain by pushing the limits of what is experimentally feasible in monkey models. By employing optotagging methods in macaques, we can begin to generate and test new hypotheses regarding how cortical circuits mediate the advanced visual abilities of primates. Ultimately, such efforts will furnish mechanistic circuit models of high-level visual computations, which can then be used to make experimental predictions for causal activity perturbations of distinct neuronal cell types. Critically, the experimental strategy we are advocating is generalizable. Here, we used optotagging to address a classically difficult problem in primate vision, but the same approach will be fruitful for investigating many other aspects of sensory, motor and cognitive function in primates.

## Methods

### Subjects and experimental procedures

Four healthy male rhesus macaque monkeys (*Macaca mulatta*) participated in the study (8–13 years; 8–13 kg). All aspects of animal care, surgical procedures and experimental protocols conformed to the *NIH Guide for the Care and Use of Laboratory* Animals, and were approved by the Institutional Animal Care and Use Committee at Columbia University.

Animal care, including housing and husbandry, was overseen by the Institute of Comparative Medicine at Columbia University. Animals had ad libitum daily access to nutrient-rich biscuits (Fiber Plus Monkey Diet Jumbo 5050, Lab Diet) and controlled daily access to fresh produce and water. Animals were single-housed, with regular access to a large play cage. Animals also had continued visual access to other animals and regular access to a television providing additional sensory enrichment. Animal cages were cleaned daily and changed biweekly. Animals were examined by a member of the veterinary staff twice daily (am and pm), and received physical exams twice yearly.

Three animals (Monkeys C, B and D) contributing neurophysiological data received an anatomical MRI scan for the purpose of generating individualized skull and brain reconstructions. These MRI-based reconstructions were used to guide the design of two custom titanium devices for each animal: (i) a head-holding device and (ii) a recording chamber centered on dorsal cortical area V4. The animals underwent two surgical procedures: a surgery in which they were implanted with the custom titanium devices, and a surgery in which they received a craniotomy providing access to brain parenchyma. A fourth animal (Monkey R) contributing only histological data underwent two surgical procedures: a surgery in which two craniotomies were performed and viral vectors were injected into the brain, and a perfusion surgery prior to brain extraction.

The animals underwent standard surgical procedures performed under aseptic conditions in a dedicated operating suite and supervised by a clinical veterinarian. Procedures requiring a craniotomy and soft tissue manipulation (e.g., implantation surgeries and the AAV vector injections performed in Monkey R) used ketamine/dexmedetomidine induction with isoflurane and adjunct propofol maintenance. Postoperative analgesia consisted of an opioid combined with an NSAID, as prescribed by veterinary staff. AAV vector injections performed in the awake state and through an existing recording chamber (in Monkeys C, B and D) did not require anesthesia or analgesia.

#### AAV vector production

Two AAV viral vectors were used in this study: AAV1–CaMKIIa–ChR2–eYFP and AAV9–mDlx5/6–ChR2–mCherry, with vector titers ranging from 6–8 × 10^12^ genomes/mL (Supplementary Table S1). AAVs were produced in our laboratory using conventional triple-plasmid transfection of human embryonic kidney cells (HEK293T; ATCC CRL-3216) with Polyethylenimine (Polysciences, PEI MAX, 24765). AAV vector production methods have been detailed in a previous study and are described briefly below^76^. Cells were cultured in Dulbecco’s Modified Eagle’s Medium containing 10% fetal bovine serum, penicillin (50 U/ml) and streptomycin (50 µg/ml), and they were incubated at 37 °C with 5% CO_2_. Cells were harvested after 72 h of incubation and pelleted by centrifugation. Vectors were released from cells by repeated freeze-thaw cycles, purified by ultracentrifugation through an iodixanol gradient column (Sigma-Aldrich, OptiPrep Density Gradient Medium, D1556), and exchanged into phosphate buffered saline by centrifugation (Cytiva, 100 K Omega, Clear Microsep Advanced Centrifugal Device, MCP100C41). Vectors were titered by qPCR and tested for fungal contamination and endotoxins (<0.13 EU/ml).

#### AAV vector injections

Prior to making AAV vector injections, all four macaques were screened for neutralizing antibodies against AAV serotypes, confirming minimal native immunity to AAV1 and AAV9 serotypes (Franklin Biolabs)^77^. Three animals contributing neurophysiological data (Monkeys C, B and D) were injected with AAVs in the awake state, guided by neurophysiological recordings in V4 cortical regions of interest. These AAV vector injections occurred within existing recording chambers and thus did not require anesthesia or analgesia. A fourth animal (Monkey R) contributing only histological data was injected with AAVs in the anesthetized state, guided by stereotaxic coordinates targeting V4 cortex and following standard surgical procedures for anesthesia and analgesia described earlier.

AAV vector injection methods have been detailed in previous studies and are described briefly below^76,78^. AAVs were delivered to the brain via a flexible cannula made of fused silica capillary tubing (Polymicro Technologies; Molex 1068150024) connected to a Hamilton syringe (Hamilton 81020) held in a manual infusion/withdrawal pump (Stoelting 51222). The cannula and syringe were filled with super low-viscosity silicone fluid (Clearco Products, Octamethyltrisiloxane, 107-51-7) mixed with fluorescent leak detection dye (Tracerline, Dye-Lite, TP-3090) and filter-sterilized by passage through a syringe filter (EMD Millipore, Millex-GS 0.22 µm, SLGS033SB). The fluorescent dye facilitated visualization of the meniscus between vector and silicone fluid under blue light illumination during the injection procedure.

AAV vector injections were made at multiple sites throughout the depth of V4 gray matter, starting from the deepest point and spaced 500 µm apart. We injected along a ~ 5 mm track, starting slightly below V4 gray matter and ending slightly above brain surface. Because the recording chambers were placed very laterally (centered ~25 mm from midline), the injection and recording tracks often traversed a highly curved portion of dorsal area V4, and gray matter thickness was estimated to be ~2.5–3.0 mm based on brain reconstructions generated from anatomical MRI scans. At each site, we injected 2 µl of vector slowly over the course of 2 min, followed by a 2-min wait period before moving to the next site. We waited an additional 10 minutes after the final injection to avoid the vector escaping along the injection track. AAV titers and volumes for each animal are provided (Supplementary Table S1). Two animals were injected with higher vector volumes to accommodate their participation in other studies.

#### Neurophysiology

Extracellular recordings of brain voltage signals were carried out using single tungsten microelectrodes (FHC, 30072). A microelectrode and an optical fiber were lowered into the brain through a common guide tube, using a custom microdrive system (Narishige, MO–96–S) allowing independent control of each component. Raw voltage signals (0.3 Hz–7.5 kHz) were recorded using a neural recording system (Blackrock Neurotech, Cerebus, 64-channel). Spike waveforms were detected by filtering the raw voltage signals (0.3–250 Hz) and extracting events that were 2.7 standard deviations above the noise. The waveforms of single neurons were then isolated manually using spike-sorting software (Plexon Systems, Offline Sorter).

We strived to ensure identical recording quality for both optotagged excitatory and inhibitory neurons. First, we applied identical recording filter settings and spike detection and extraction procedures for all neurons, regardless of cell type. Second, we used the same spike-sorting procedures for all neurons; we only included data from well-isolated neurons, with fewer than 1% of spike intervals shorter than 1 ms^79^. Third, we conducted post-hoc autocorrelation analyses for all sorted neurons and confirmed that their auto-correlograms exhibited a clear refractory period, consistent with well-isolated single-unit activity.

The recording chambers were centered on dorsal area V4 along the prelunate gyrus, approximately 22–26 mm lateral of midline and 8–10 mm posterior of inter-aural zero. Optotagged neurons had receptive fields in the contralateral, inferior visual quadrant at 2.5°–11.5° of eccentricity (median 7.7°) and preferred stimulus sizes 0.5°–8.5° in diameter (median 3.5°) (Supplementary Fig. S10).

#### Optical stimulation

Approximately 7–10 weeks after AAV vector injections were made, we began searching for responses to blue light in cortical area V4 (Supplementary Table S1). Light (450 nm; 4–119 mW; median ~90 mW) was delivered to the brain via an optical fiber (Thor labs, FT300UMT; 300 µm) connected to a custom laser modulation device (Washington National Primate Research Center, Bioengineering group). For each optotagged neuron, we chose the minimal laser power needed to elicit clear, time-locked neural responses based on manual tests. In most sessions, we also manually confirmed selective neuronal responses to blue (450 nm) but not red (638 nm) light illumination, thereby mitigating concerns about thermal or light artifacts. Laser trial events were controlled by custom open-source software (MWorks). Optotagged neurons were usually identified when the distance between the electrode tip and optical fiber tip was within 500 µm.

Two patterns of optical stimulation were used: sinusoidal (2–500 Hz) and square wave (16 or 32 Hz). We used sinusoidal patterns in most trials for two key reasons. First, sinusoidal patterns deliver lower overall light power and have less abrupt light transitions compared to square wave patterns, due to the gentler rise and fall phases—properties that may confer network stability during optotagging. Second, sinusoidal patterns allow us to characterize how excitatory and inhibitory neurons respond to temporally varying patterns of optical stimulation (i.e., by computing F1 responses). Such functional comparisons across cell types are helpful for identifying optimal optical stimulation parameters for future cell type-specific perturbation experiments in macaques.

In our experience, sinusoidal patterns work well for optotagging cortical excitatory and inhibitory neurons, as well as cerebellar Purkinje cells in macaques^78^. Nevertheless, we anticipate that other groups may wish to use standard square wave patterns for optotagging. For this reason, we also included the square wave condition in our experiments. Activation latencies obtained using sinusoidal and square wave patterns were highly correlated (Supplementary Fig. S3), confirming that future studies can also use square wave modulation for optotagging.

#### Visual stimulation

Visual stimuli were presented on a calibrated research-grade LCD display with a resolution of 1920 × 1080 pixels and a refresh rate of 120 Hz (VPixx, VIEWPixx 3D). The display was positioned directly in front of the animals, at a distance of 50–56 cm. The animals passively viewed visual stimuli on the display while maintaining fixation of a 0.15° central point within a window of radius 1–1.5° in return for liquid rewards (juice or water). Eye position was monitored using an infrared-based tracking system (SR Research, Eyelink 1000 Plus). Stimuli were presented for 300 ms each, with an inter-stimulus interval of 200 ms. A subset of trials had no visual stimuli to facilitate the measurement of spontaneous activity. Stimuli were presented against an achromatic gray background. Stimulus onset and offset times were based on photodiode detection of synchronized pulses in the upper left corner of the display. Stimulus generation and behavioral trial events were controlled by custom open-source software (The MWorks Project, MWorks).

We tested optotagged neurons using two classes of visual stimuli: traditional bar stimuli and two-dimensional shape stimuli (Fig. 4A, B). Bar stimuli were presented at 8 orientations (0–157.5° in 22.5° steps). Shape stimuli were presented at 8 rotations (0–315° in 45° steps). Trials containing bar and shape stimuli were randomly interleaved to ensure identical recording conditions. Most neurons were tested with both stimulus classes; 2 neurons (one excitatory, one inhibitory) recorded in our earliest data collection sessions were only tested with shape stimuli. Stimuli were presented at an approximately preferred color, position and size, as identified in preliminary experiments.

#### Data inclusion criteria

The study is based on data collected from 119 optotagged neurons: 59 excitatory neurons and 60 inhibitory neurons (Supplementary Table S2; 59 neurons from Monkey C; 19 from Monkey B and 41 from Monkey D). We also include data collected from 165 neurons recorded without knowledge of their cell type identity, herein referred to as non-optotagged neurons (Supplementary Table S2; 83 neurons from Monkey C; 20 neurons from Monkey B and 62 neurons from Monkey D). Non-optotagged neurons were sampled within the same cortical sites where we injected AAV vectors and where we recorded optotagged neurons. Non-optotagged neurons were recorded at similar microdrive x/y coordinates where optotagged neurons were recorded, but in separate experimental sessions (e.g., during routine experiments for basic receptive field mapping and other data collection sessions conducted before and after AAV vector injections were made). This approach ensured that data from non-optotagged neurons could serve a suitable benchmark for functional comparisons between optotagged neurons and the generalist population of V4 nearby.

We ensured that all optotagged neurons in our dataset had receptive fields consistent with the known topography of dorsal area V4^80^ (Supplementary Fig. S10). In less than a handful of recording sessions, we encountered optotagged neurons deep in gray matter that had much larger and/or more peripheral receptive fields, consistent with neurons located in the superior temporal sulcus. In these rare cases, we elected not to test these neurons further, so as to ensure correct identification of neurons within V4.

To make functional comparisons between excitatory and inhibitory neurons recorded across a limited number of animals, we strived to equate three major factors. First, we ensured similar visual response properties at the injection sites of all three animals (Supplementary Fig. S9A). Second, we sampled a similar total number of optotagged excitatory and inhibitory neurons (E = 59; I = 60; Supplementary Table S2). Third, we sampled optotagged neurons within a similar area of V4 cortex (E = 17.5 mm^2^; I = 15.5 mm^2^; Supplementary Table S2).

Our data inclusion criteria were three-fold. First, only well-isolated neurons were included. Second, for experiments characterizing the electrophysiological responses of optotagged neurons, at least 8 repeats of each optical stimulation condition were collected (the median was 15 repeats; the range was 8–20 repeats). Third, for experiments characterizing the visual responses of optotagged neurons, at least 3 repeats of each visual stimulus condition were collected (the median was 7 repeats; the range was 3–20 repeats).

#### Histology

We examined AAV vector-mediated opsin expression histologically in one macaque that did not contribute neurophysiological data (Monkey R; Supplementary Table S1). This animal was injected in a surgical procedure using the same viral vector aliquots and methods used for the other three animals. Following a survival period of approximately two months, the animal was euthanized with Euthasol and perfused through the heart with 1X PBS solution followed by 4% paraformaldehyde and a gradient of sucrose in 1X PBS (10, 20 and 30% sucrose). The brain was extracted and cryoprotected in 30% sucrose. Sagittal sections (50 μm thick) were cut on a sliding microtome and mounted onto slides. Transduced cells were first localized by inspecting native fluorescence signals. Sections near regions of interest were then processed using primary antibodies: chicken anti-GFP (Abcam 1,3970, 1:1000) and mouse anti-mCherry (Takara/Clontech 632543, 1:250), and rabbit anti-Parvalbumin (Swant PV27a, 1:1000). The sections were subsequently processed using secondary antibodies against: donkey anti-chicken Alexa Fluor 488 (Jackson Immuno Research 703-545-155, 1:400), donkey anti-mouse Alexa Fluor 568 (Invitrogen A10037, 1:5000), donkey anti-rabbit Alexa Fluor 488 (Invitrogen A21206, 1:500) and donkey anti-rabbit Alexa Fluor 568 (Invitrogen A10042, 1:500). Finally, the sections were processed with the nuclear stain DAPI (Invitrogen Molecular Probes D-21490, 1:10,000) and were prepared for visualization by epifluorescence and confocal microscopy (Fig. 1, Supplementary Fig. S1 and S2).

In the case of both viral vectors used, we observed robust AAV-mediated ChR2 expression in many neurons, spanning ~2–4 mm of V4 cortex. At sites injected with AAV–CaMKII–ChR2–eYFP, we observed strong ChR2 expression that extended throughout apical and basal dendrites and long-range projection axons, all of which are diagnostic morphological features of excitatory neurons but not inhibitory neurons (Supplementary Fig. S1). This strong expression in excitatory neuron processes hindered our ability to clearly visualize excitatory cell bodies across cortical layers. Thus, for the main histological figure (Fig. 1C), we selected a histological section that was 3 mm away from the AAV vector injection site, and we used lower antibody concentrations so as to optimally reveal a few excitatory cell bodies across cortical layers.

### Quantification and statistical analysis

All analysis was performed in Matlab (Mathworks) as described below. All hypothesis testing procedures were non-parametric (Wilcoxon rank sum test, implemented in Matlab as ‘p= ranksum(x,y)’; this is a two-sided test).

#### Extracellular waveform features

To characterize the extracellular spike waveforms of optotagged neurons, we calculated two commonly used measures of waveform shape: (i) the time from trough to peak; and (ii) the repolarization time (Fig. 3B; see inset on left). For each optotagged neuron, we aligned the waveforms recorded in response to all laser conditions to the dominant trough, and computed the average waveform for use in further analyses. We calculated the trough–to–peak time as the interval between the first trough and the first peak^32–34^. We calculated the repolarization time as the interval between the peak and the inflection point of the following falling flank of the curve^56,81^. We performed this waveform analysis only for neurons with waveforms characterized by a dominant trough followed by a dominant peak (50 E and 56 I). Neurons with triphasic waveforms were excluded from this analysis (9 E and 4 I). The average waveforms of all excitatory and inhibitory neurons identified by optotagging are provided (Supplementary Fig. S5).

To determine whether excitatory and inhibitory neurons differed with respect to their joint waveform features (i.e., trough–to–peak time and repolarization  time), we performed a shuffle analysis. The goal of this analysis was to assess the likelihood that the observed separation between the centroids of excitatory and inhibitory neuron clusters could have occurred by chance (Fig. 3B). We first calculated the average trough–to–peak time and repolarization time for each neuronal cell type, and we took the pairwise distance between their average values as the observed separation between the neuronal cell types in the two-dimensional waveform feature space. On each iteration of the shuffle procedure, we randomly shuffled the labels for excitatory and inhibitory neuron data and drew simulated populations of the same size as our datasets, and we calculated the average separation between the centroids of the simulated populations. Finally, we used the distribution of separations generated across all iterations of the shuffle analysis (*N* = 1000) to calculate the probability that the observed separation for optotagged data could have occurred by chance. We found that the observed separation between optotagged excitatory and inhibitory neurons was statistically significant (*p* < 0.001). Thus, this shuffle analysis confirmed that the centroids of spike waveform features for excitatory and inhibitory neurons were significantly different.

To test whether we could predict cortical cell type based on waveform features, we performed a cross-validation analysis based on a linear classifier approach. In this analysis, we assessed the ability of a linear classifier to discriminate between excitatory and inhibitory neurons based on waveform features (using Matlab’s fit discriminant analysis classifier ‘fitcdiscr’). On each iteration of the cross-validation procedure, we partitioned the optotagged data into training and testing sets (90% train, 10% test), and we trained a linear classifier on the train set and used it to predict whether neurons in the test set were excitatory or inhibitory. Finally, we calculated the fraction of correct classifications across all iterations of the cross-validation analysis (*N* = 1000). This analysis produced a distribution of classification accuracies, with an average performance accuracy of 63%. Thus, we could accurately predict cell type based on extracellular waveform parameters in only ~60% of cases.

#### Responses to optical stimulation

For each optotagged neuron, we generated raster plots and peri-stimulus time histograms (PSTHs) from the responses to each laser modulation condition (Fig. 2A, B). From these responses, we calculated several quantities of interest, as described below.

##### Activation latency

To quantify the latency of optogenetic activation, we pooled each neuron’s responses across low (2–8 Hz), mid (16–64 Hz) and high (129–500 Hz) sinusoidal modulation conditions, and generated PSTHs for each of these conditions (0.2 ms bins). We also calculated baseline activity during the 200 ms period prior to laser onset. We calculated activation latency as the first time point when responses in two consecutive PSTH bins reached 3 standard deviations above baseline activity. We also performed the same latency analysis for square wave modulation conditions (16 or 32 Hz). For each optotagged neuron, we chose the shortest latency value across all laser conditions as the activation latency.

##### Responsiveness

To quantify the robustness of responses to optical stimulation, we calculated each neuron’s average firing rate over the laser stimulation period (500 ms) for each laser condition. We then took the peak response across all laser conditions as a measure of responsiveness for each neuron.

##### Preferred frequency

To quantify the preferences of optotagged neurons for sinusoidal modulation conditions, we calculated the response amplitude at each laser modulation frequency condition (F1 response) and fitted these responses with a log-Gaussian function^78,82^. The model fits provided a good description of observed responses. From these fits, we extracted two quantities for each neuron: (i) the preferred laser frequency, corresponding to the peak response and (ii) the high-frequency cutoff, corresponding to the highest frequency eliciting half of the peak response^78^.

#### Responses to visual stimulation

We started each data collection session by attempting to drive each optotagged neuron, under passive viewing conditions, using a standard battery of visual stimuli commonly used in V4 studies. The stimuli included drifting sinusoidal gratings, spots of light, oriented bars and two-dimensional geometric shapes; the stimuli could also vary in position, size, color and rotation, under the experimenter’s control. Based on this initial and qualitative receptive field mapping procedure, we classified each optotagged neuron as visually responsive or unresponsive. Many optotagged neurons were visually responsive, but some were determined to be unresponsive (Fig. 5A). In the case of unresponsive neurons, we always confirmed that we could readily activate nearby neurons using the same battery of stimuli, thereby confirming the suitability of our stimuli for driving nearby V4 neurons. We then proceeded to characterize visually responsive neurons in quantitative experiments. For each visually responsive neuron, we calculated the average response for each stimulus condition (bar and shape stimuli) during the stimulus presentation period (300 ms). These responses were the basis for several analyses, described below.

##### Visual response latency

To calculate the time at which each V4 neuron became visually responsive, we generated PSTHs (0.5 ms bins) from the responses across all stimulus conditions. We then smoothed the PSTHs by applying a sliding boxcar filter (30 ms bins; 1 ms sliding window). Based on these smoothed PSTHs, we identified visual latency as the time at which the response reached 20% of its peak value^83^.

##### Bar orientation selectivity

For each neuron, we computed the average response to each stimulus condition during a 300 ms epoch starting from visual response latency. To quantify selectivity for bar orientation, we fit the average responses of each neuron to all bar stimuli (0–157.5°) using a modified von Mises distribution function^84,85^. This modified function accounted for two possible response patterns observed in V4: some neurons showed preference for a single bar orientation whereas others showed a preference for two bar orientations that were often (but not always) orthogonal^1,84,85^. The model fit provided a good description of observed responses for all optotagged neurons. Based on this fit, we calculated an index of bar orientation selectivity as follows:

1 $${{{Bar}\; {orientation}\; {selectivity}\; {index}}}=[{{{R}}}_{{{preferred}}}-{{{R}}}_{{{null}}}]/[{{{R}}}_{{{preferred}}}+{{{R}}}_{{{null}}}]$$

where *R*_preferred_ is the average response at the preferred orientation and *R*_null_ is the average response at the orientation eliciting the weakest response. Index values ranged from 0 to 1, with higher values indicating stronger orientation selectivity. Neurons with good model fits and selectivity indices ≥0.2 were classified as selective for bar orientation information.

##### Contour shape selectivity

For each neuron, we computed the average response to each stimulus condition during a 300 ms epoch starting from visual response latency. To quantify selectivity for contour shape, we fit the responses of each neuron to all shape stimuli (104 stimuli; i.e., 13 shapes at each of 8 rotations) using a well-established quantitative model^3^. This model has been used previously to fit the responses of V4 neurons to exactly the same stimuli used in this study; fitting procedures have been detailed elsewhere and are described briefly below^11^. We fit each neuron’s responses with a model that incorporated four parameters of contour shape: angular position, primary curvature, and two adjacent curvatures (clockwise and counterclockwise)^3,11^. Based on this model fitting procedure, we calculated an index of contour shape selectivity as follows:

2 $${{{{Contour}\; {shape}\; {selectivity}\; {index}}}}_{{{model}}-{{driven}}}={{corr}}({{{R}}}_{{{observed}}},\,{{{R}}}_{{{predicted}}})$$

where *corr* is the correlation coefficient, *R*_observed_ is the observed response to each shape stimulus and *R*_predicted_ is the predicted response to each shape stimulus. Intuitively, the index captures how well each neuron’s responses can be accounted for in terms of a preference for a curved contour feature at a specific position relative to object center. Index values ranged from 0 to 1, with higher values indicating stronger shape selectivity. Neurons with selectivity indices ≥0.7 were classified as selective for contour shape information. This stringent criterion was chosen based on previous V4 studies and ensured unambiguous classification of shape-selective neurons^3,11^.

To evaluate the statistical significance of our shape selectivity index, we conducted a shuffle analysis. For each optotagged neuron, we repeatedly shuffled the identity of the stimulus, then constructed a tuning curve from the shuffled responses, and computed a shape selectivity index for the shuffled data (*N* = 2000 shuffles). Based on the distribution of shuffled shape selectivity indices for each neuron, we evaluated the probability that the observed index was larger than expected by random sampling. This shuffle analysis showed that the shape selectivity indices for many neurons were significant, further confirming the difference in shape selectivity between excitatory and inhibitory neurons (Supplementary Fig. S8C).

To confirm the robustness of our shape selectivity index, we conducted a cross-validation analysis. For each optotagged neuron, we fit the model on 80% of the data and then used the fit to predict responses to the 20% of held-out data, and we documented the model’s performance on both datasets (*N* = 50 iterations). We then computed the shape selectivity index as the average model performance across all iterations of the cross-validation procedure. This cross-validation analysis confirmed that the difference in shape selectivity between excitatory and inhibitory neurons was statistically significant for both test and train data (Supplementary Fig. S8C).

In addition to our model-driven shape selectivity index described above, we also evaluated an alternative data-driven measure of shape selectivity. For each optotagged neuron, we calculated a modulation index based on the responses to shape stimuli at the optimal rotation (i.e., 13 shapes at 1 rotation). Intuitively, this index captures how much of the neuron’s observed responses modulate around the average firing rate, and is calculated as follows:

3 $${{Contour}\; {shape}\; {selectivity}\; {index}}_{{data}-{driven}}={variance}({{{R}}}_{{{observed}}})/{{mean}}({{{R}}}_{{{observed}}})$$

where *R*_observed_ is the observed response to each shape stimulus at the optimal rotation. Index values ranged from 0 to 6.5, with higher values indicating stronger selectivity. This data-driven measure also showed a significant difference in shape selectivity between excitatory and inhibitory neurons (Supplementary Fig. S8E).

##### Time–to–selectivity

For each neuron that was selective for bar orientation, contour shape or both, we identified the time at which selectivity first reached criterion levels for classification^86^. To this end, we calculated responses to all stimulus conditions over progressively longer epochs, extending through the stimulus presentation period (starting from each neuron’s visual response latency and extending to 300 ms after that latency, in 10 ms increments; Fig. 6A). We then fit the responses in each epoch as described earlier. Based on the model fits, we identified the first time point at which selectivity exceeded a criterion level enabling reliable classification for two consecutive response epochs. Time–to–selectivity for bar orientation was defined as the first time point when bar orientation selectivity index was ≥0.2. Time–to–selectivity for contour shape was defined as the first time point when contour shape selectivity index was ≥0.7. Data showing the entire time course of each neuron’s selectivity for bar orientation and contour shape are provided (Supplementary Fig. 13A, B). For additional verification of our findings, we also used a different measure of the temporal dynamics of visual form selectivity. In this case, time–to–selectivity was defined as the first time point at which the selectivity index reached 90% (or 80%) of its final value for two consecutive time bins; this analysis yielded qualitatively similar results (Supplementary Fig. 13C).

##### Sustained response index

For each neuron, we generated a PSTH (1 ms bins) from the responses across all stimulus conditions (bars and shapes). We then calculated the total area under the PSTH that exceeded baseline during the stimulus presentation epoch (0–300 ms), and we normalized the resultant by the total area. We then partitioned the normalized area under the PSTH into two equally sized epochs: an early epoch (0–150 ms) and a late epoch (150–300 ms). Based on the area for each epoch, we calculated a sustained response index as follows:

4 $${\rm{Sustained}\; {\rm{response}}\; {\rm{index}}}={{\rm{PSTH}}\; {\rm{area}}}\,_{{\rm{Late}}}/{{\rm{PSTH}\; {\rm{area}}}}\,_{{\rm{Early}}}$$

where *PSTH area*_Late_ is the area under the PSTH in the late epoch (150–300) and *PSTH area*_*Early*_ is the area under the PSTH in the early epoch (0–150). Index values ranged from −0.04 to 1.45, with higher values indicating a stronger sustained response (Fig. 6E).

### Reporting summary

Further information on research design is available in the Nature Portfolio Reporting Summary linked to this article.

## Supplementary information

Supplementary Information

Reporting Summary

Transparent Peer Review file

## Source data

Source data

## Data Availability

The full dataset is available in a public repository: https://doi.org/10.5281/zenodo.21342746. Source data are provided with this paper.

## Code availability

Code for data analysis is available in a public repository: https://doi.org/10.5281/zenodo.21342746.
