# Supplementary material for: Optotagging in primate cortex uncovers excitatory and inhibitory neuron specializations in form vision
Source: Nat Commun. 2026 Aug 26;17:10269. doi: 10.1038/s41467-026-76521-4 (PMC13616898; doi:10.1038/s41467-026-76521-4)
Supplement: Supplementary file 1 [file 41467_2026_76521_MOESM1_ESM.pdf]

SUPPLEMENTARY INFORMATION

Optotagging in primate cortex uncovers excitatory and inhibitory neuron specializations in form vision

Chuyi Su[1,2] and Yasmine El-Shamayleh[1,2,3,*]

[1] Zuckerman Mind Brain Behavior Institute, Columbia University, New York, NY, 10027, USA

[2] Department of Neuroscience, Columbia University, New York, NY, 10027, USA

[3] Kavli Institute for Brain Science, Columbia University, New York, NY, 10027, USA

* Correspondence: yasmine.shamayleh@columbia.edu (Y.E.S.)

Contents

- **Supplementary Tables S1–S2**
- **Supplementary Figures S1–S14**

SUPPLEMENTARY TABLES

Monkey	Vector	Vector titer	Vector volume	Time from vector injection to first data collection
C	AAV1–CaMKIIa–ChR2–eYFP	6 x 10^{12} genomes/mL	20 µl	69 days
B	AAV9–mDlx5/6–ChR2–mCherry	8 x 10^{12} genomes/mL	40 µl	70 days
D	AAV9–mDlx5/6–ChR2–mCherry	8 x 10^{12} genomes/mL	40 µl	52 days
R	AAV9–mDlx5/6–ChR2–mCherry	8 x 10^{12} genomes/mL	20 µl	68 days
R	AAV1–CaMKIIa–ChR2–eYFP	6 x 10^{12} genomes/mL	20 µl	68 days

Supplementary Table S1. Viral vectors injected in each animal. AAV vectors were injected into the cerebral cortex of four macaques. AAV vectors were produced in the laboratory, using plasmids pAAV-CamKIIa-hChR2(H134R)-eYFP (Addgene #26969; deposited by the lab of Dr. Karl Deisseroth) and pAAV-mDlx-ChR2-mCherry-Fishell-3 (Addgene #83898; deposited by the lab of Dr. Gordon Fishell). Vector titers and volumes are provided. We began searching for neuronal responses to optical stimulation in Monkeys C, B and D approximately 7–10 weeks after the injections were made. Monkeys B and D received larger volumes of vector to accommodate participation in other studies. We began histological analysis in Monkey R approximately two months after the injections were made.

Monkey	Optotagged neurons	Non-optotagged neurons	# Penetrations	Area sampled (mm^2)
C	59 Excitatory	83	70	17.5
B	19 Inhibitory	20	20	5
D	41 Inhibitory	62	72	10

Supplementary Table S2. Extent of cortical sampling in each animal. We recorded optotagged and non-optotagged neurons within the same cortical sites in three macaques, but in separate experimental sessions. We sampled a similar number of optotagged excitatory and inhibitory neurons (59 E and 60 I), as well as a similar number of non-optotagged neurons across injection sites (83 in AAV–CaMKIIa sites and 82 in AAV–mDlx5/6 sites). Optotagged and non-optotagged neurons were recorded across many electrode penetrations and over a large area of V4 cortex. This broad sampling approach increased the likelihood of surveying various functional domains within V4. The extent of cortical area sampled is derived based on the microdrive coordinates of each electrode penetration. The microdrive is aligned to a fiducial mark on each recording chamber, thereby enabling relative measurements of electrode penetration positions over days.

SUPPLEMENTARY FIGURE S1

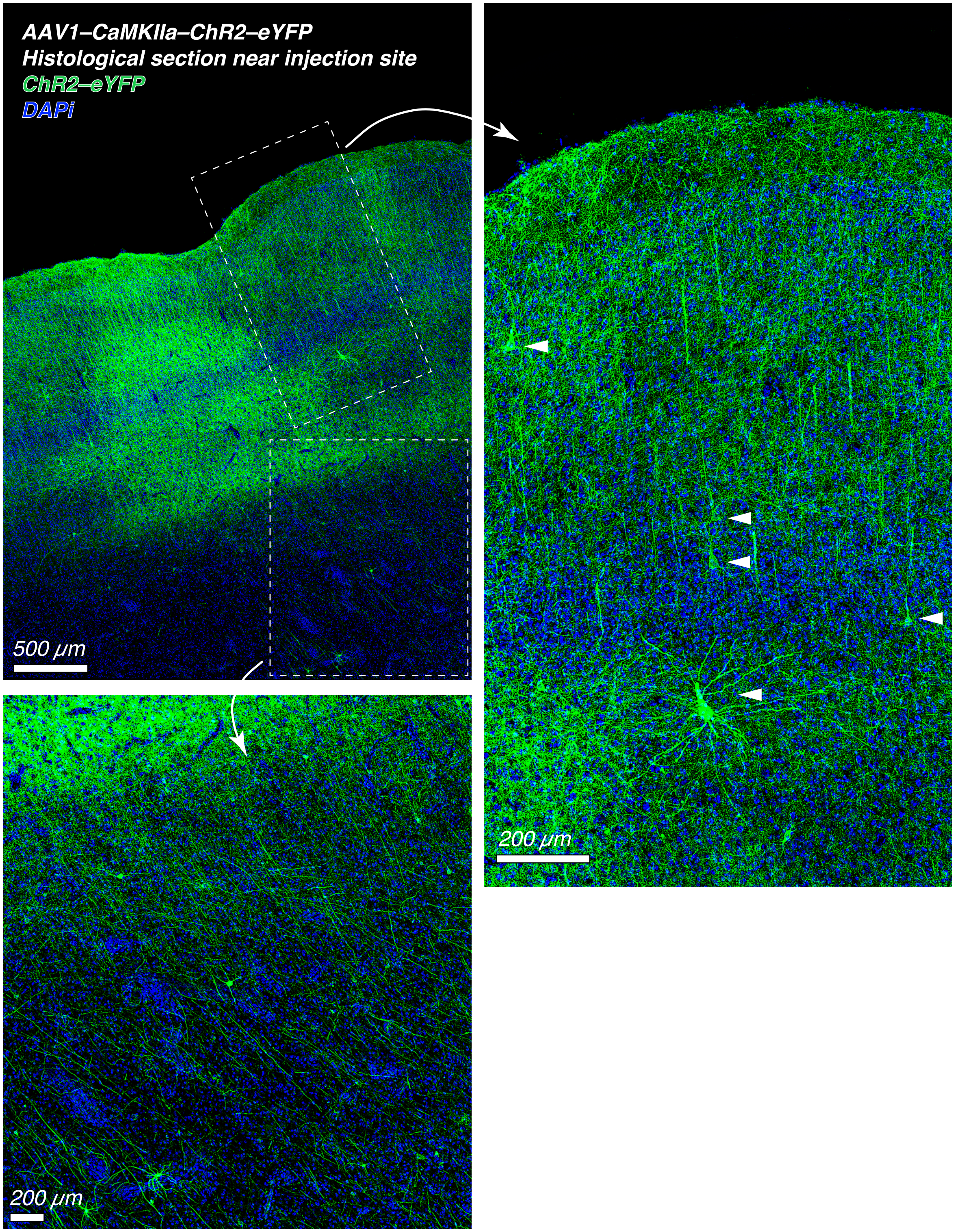

Supplementary Figure S1. ChR2 expression close to the CaMKIIa vector injection site. Top left: Histological analysis of a V4 cortical site in Monkey R injected with AAV1–CaMKIIa–ChR2–eYFP (20X montage; scale bar corresponds to 500 μm). ChR2-positive neurons and processes are in green; nuclei stained by DAPi are in blue. The image shows strong expression across most cortical layers (except layer 4), including in apical and basal dendrites and in axons—all of which are characteristic features of excitatory neurons but not inhibitory neurons. A few pyramidal cell bodies are visible across layers but most are occluded by the strong expression in neuronal processes. Top right: expanded image of the region outlined in the dashed white box within gray matter, showing strong ChR2–eYFP expression in several pyramidal cells marked by arrowheads (20X montage; scale bar corresponds to 200 μm). Bottom left: expanded image of the region outlined in a dashed white box within white matter, with increased gain of the eYFP signal to show expression in projection axons in white matter clearly (20X montage; scale bar corresponds to 200 μm).

SUPPLEMENTARY FIGURE S2

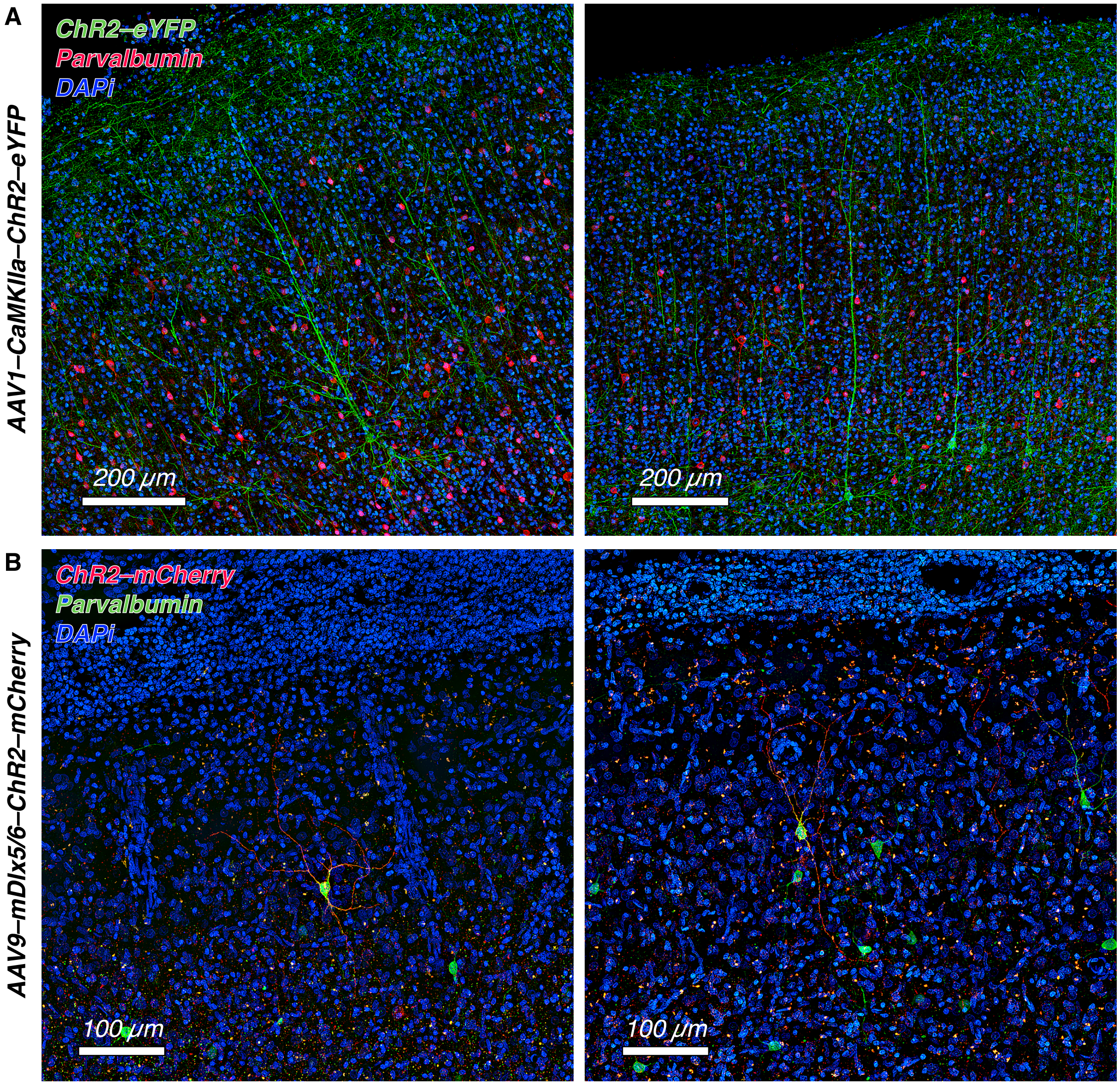

Supplementary Figure S2. Morphology of transduced neurons. (A) Higher magnification images from a site injected with AAV1–CaMKIIa–ChR2–eYFP (40X montage; scale bar corresponds to 200 *μ*m). ChR2-positive neurons are in green; parvalbumin-positive neurons are in red; nuclei stained by DAPi are in blue. ChR2-positive neurons had clear pyramidal cell morphology and were not double-labeled for the inhibitory neuronal marker parvalbumin, consistent with their classification as excitatory neurons. **(B)** Higher magnification images from a site injected with AAV9–mDlx5/6–ChR2–mCherry (40X montage; scale bar corresponds to 100 *μ*m). ChR2-positive neurons are in red; parvalbumin-positive neurons are in green; nuclei stained by DAPi are in blue. ChR2-positive neurons had non-pyramidal cell morphology and some were double-labeled for the inhibitory neuronal marker parvalbumin (see neurons at center of images), consistent with their classification as inhibitory neurons.

SUPPLEMENTARY FIGURE S3

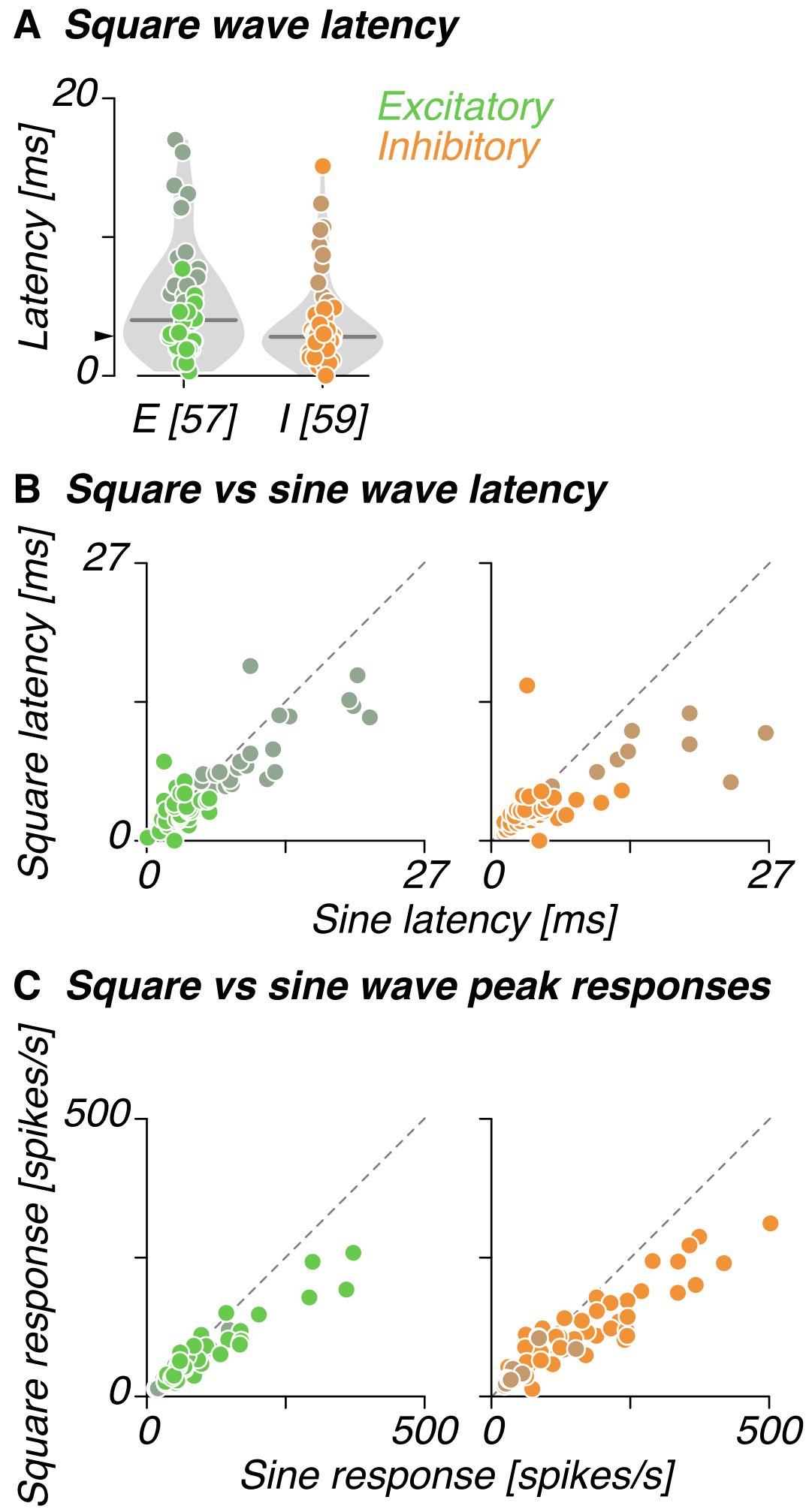

Supplementary Figure S3. Responses of optotagged neurons to sinusoidal and square wave modulation. (A) Distribution of activation latencies for excitatory and inhibitory neurons (green and orange) obtained using square wave modulation. Data from neurons with short and long activation latencies are color-coded (brighter and darker shades; arrowhead shows the latency criterion of 5 ms). Sample sizes are indicated along the abscissa. **(B)** Scatter plots of activation latencies obtained using square and sine wave modulation, showing a positive correlation (r=0.87, p<0.0001 for E; r=0.71, p<0.0001 for I). **(C)** Scatter plots of peak responses to square and sine wave modulation, showing a positive correlation (r=0.95, p<0.0001 for E, r=0.92, p<0.0001 for I). In our study, we always report the shortest activation latency and the highest peak response across laser modulation conditions for each neuron.

SUPPLEMENTARY FIGURE S4

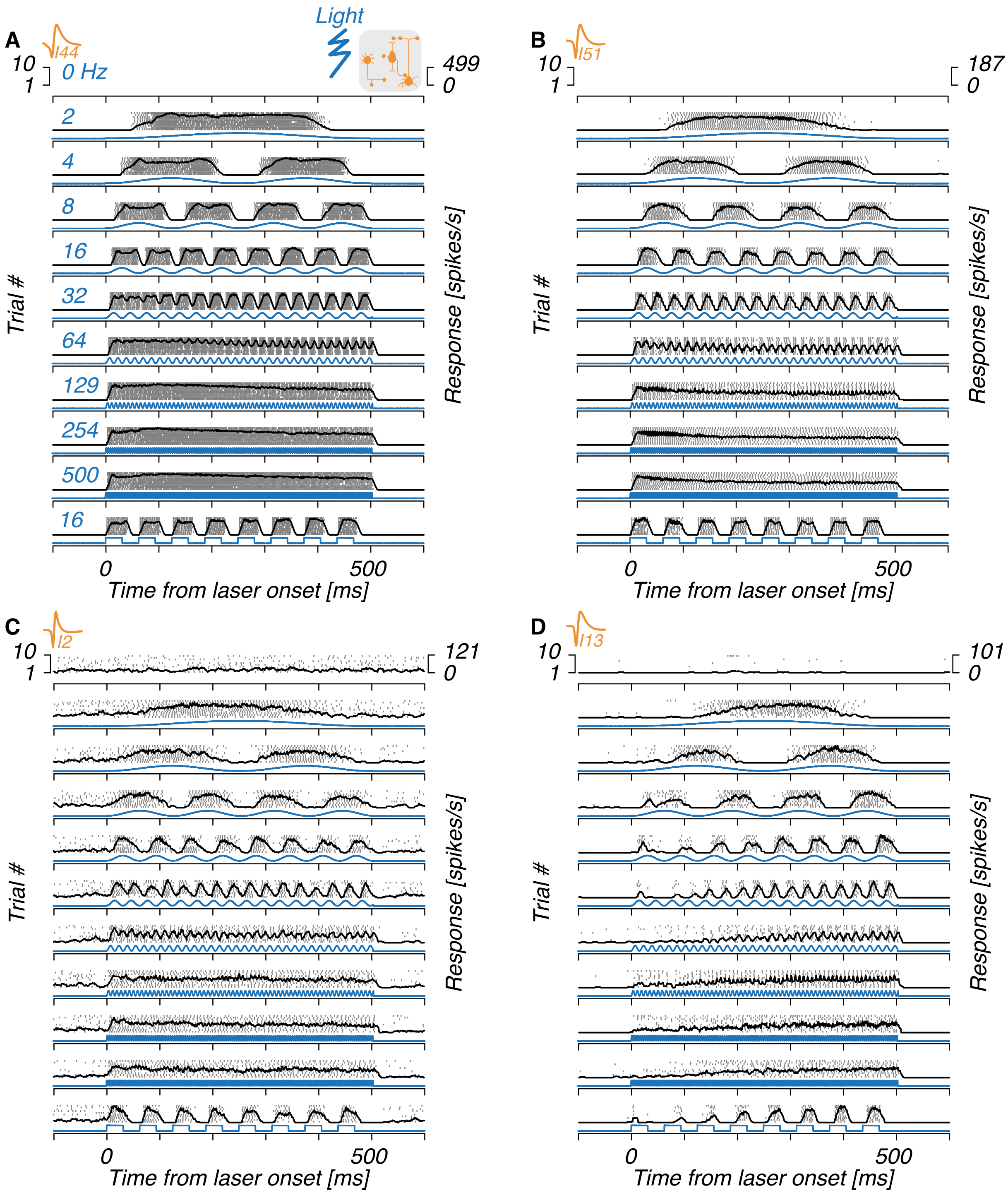

Supplementary Figure S4. Responses of inhibitory neurons to optical stimulation. Responses of four example inhibitory neurons, demonstrating a range of response patterns (same format as in **Figure 2A–B**; average waveforms are shown in orange). **(A)** Responses of an inhibitory neuron showing robust activation across all laser modulation conditions (activation latency was 1.7 ms). **(B)** Responses of an inhibitory neuron showing exquisitely precise spike times (activation latency was 2.7 ms). **(C)** Responses of an inhibitory neuron showing high spontaneous activity, fairly uncharacteristic of neurons in cortical area V4 (activation latency was 3.3 ms). **(D)** Responses of an inhibitory neuron showing a facilitating activation pattern; responses grew stronger over the laser stimulation period (activation latency was 1.7 ms).

SUPPLEMENTARY FIGURE S5

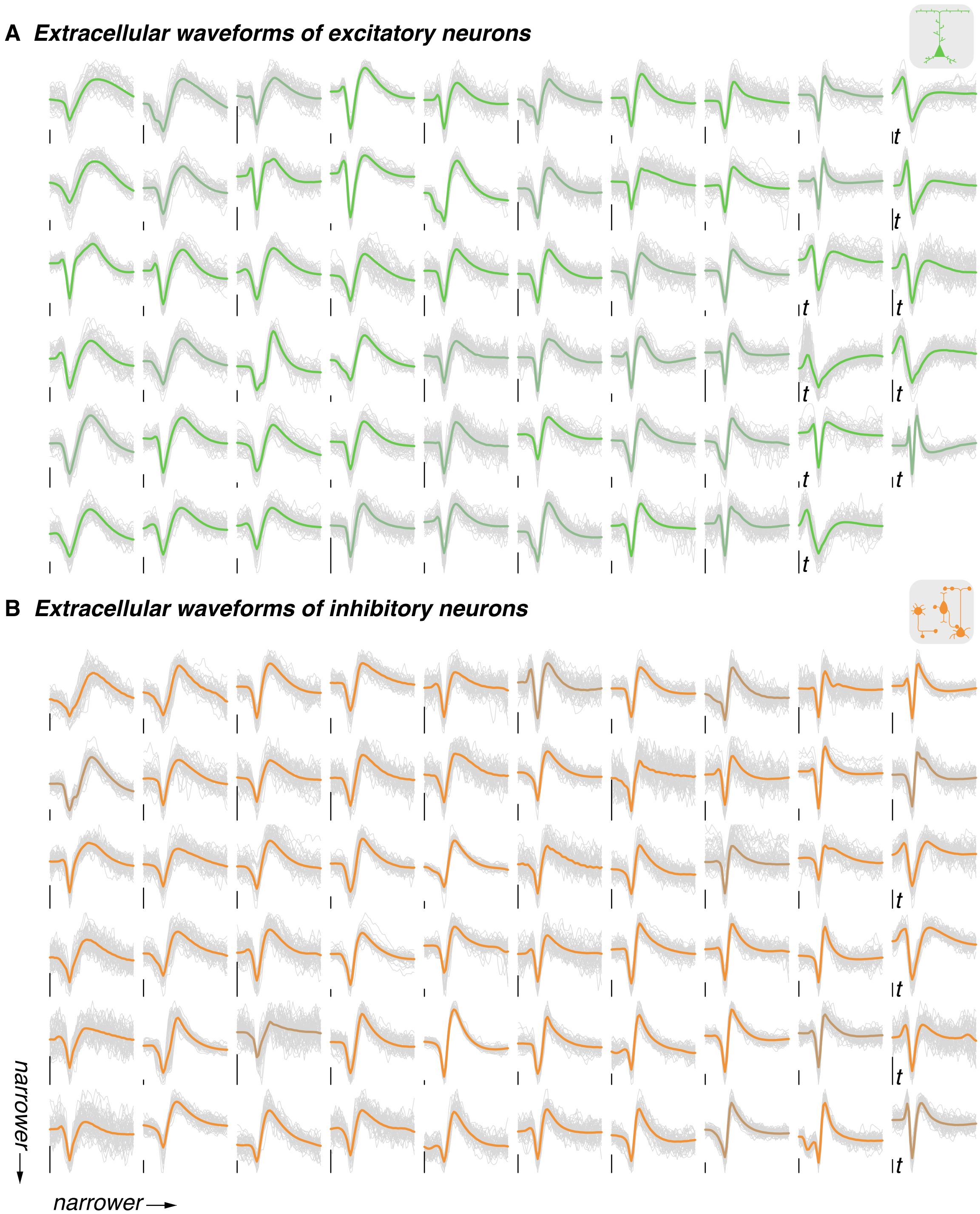

Supplementary Figure S5. Extracellular waveforms of all optotagged neurons. (A) Waveforms of all excitatory neurons (average waveforms are in green; a random selection of 50 individual waveforms in gray). **(B)** Waveforms of all inhibitory neurons (average waveforms are in orange; same format as in **A**). Waveforms are sorted by descending trough–to–peak times, both within and across columns; thus, waveforms get progressively narrower moving across columns. Only waveforms with a dominant trough followed by a dominant peak contributed to the analysis of waveform features (see **Figure 3**). Triphasic waveforms (marked with t) were excluded from further analysis. Data from optotagged neurons with short and long activation latencies (< 5ms or above) are color-coded (bright and dark shades, for short and long latencies). All waveforms had a similar duration of 1.77 ms. Scale bars under each waveform correspond to 10 μV.

SUPPLEMENTARY FIGURE S6

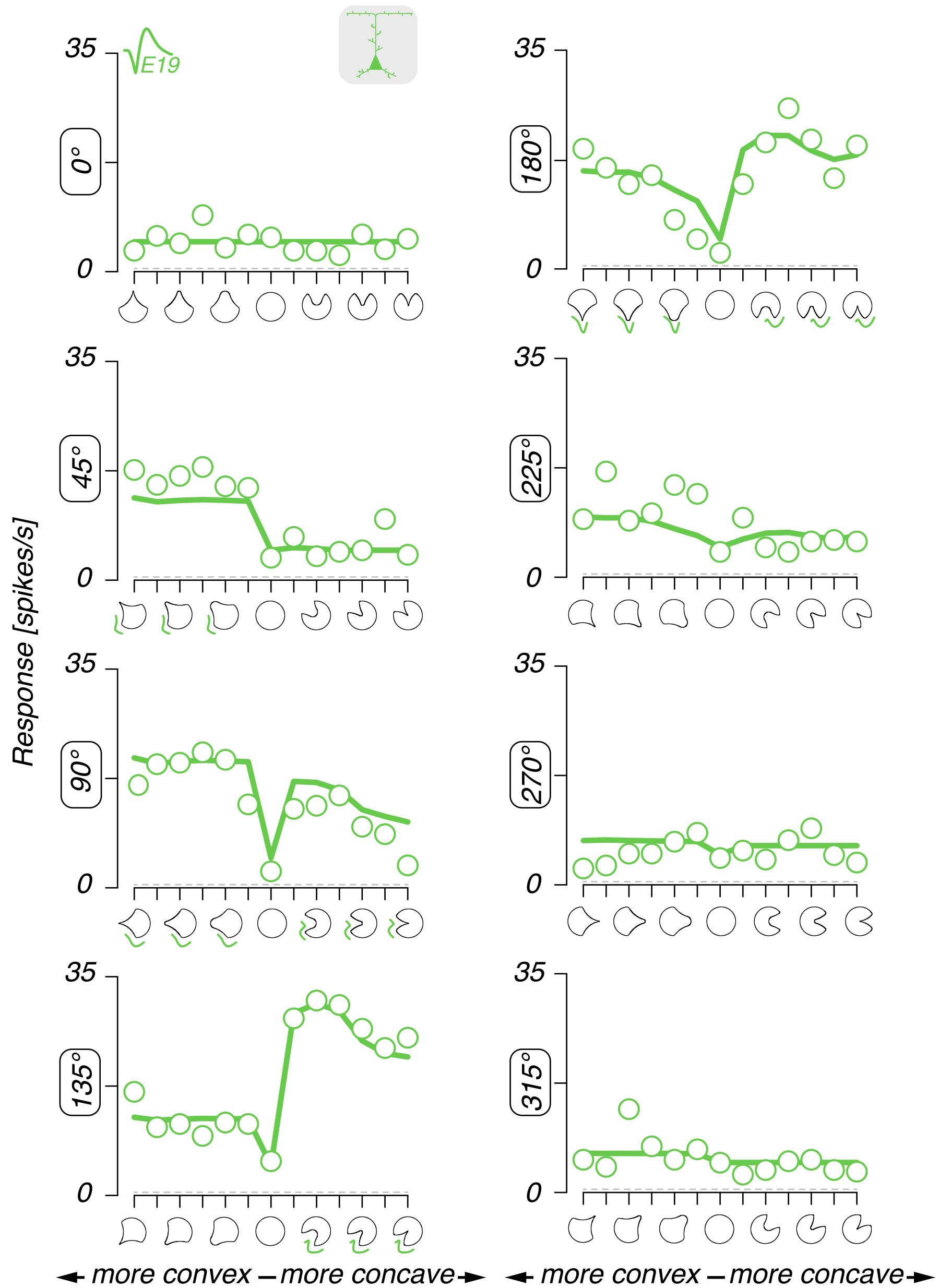

Supplementary Figure S6. Responses of an excitatory neuron to shape stimuli presented at different rotations. Responses of an example neuron to all shape stimuli (13 shapes presented at 8 stimulus rotations; same data as in **Figure 4A**). Each subplot shows responses to stimuli at one stimulus rotation (0–315° in 45° steps). The neuron responded strongly and selectively to only a subset of stimulus rotations; the optimal stimulus rotation was 135°. Responses at all stimulus rotations were jointly fit by a quantitative model of shape selectivity (lines show model fit; dashed gray line shows spontaneous activity). The model fit provided a good account of the observed responses (shape selectivity index was 0.92). The model identified a preferred contour segment that elicited strong responses at several stimulus rotations: a convexity with an adjoining concavity on one side (shown schematically as hand-drawn features in green along the abscissa).

SUPPLEMENTARY FIGURE S7

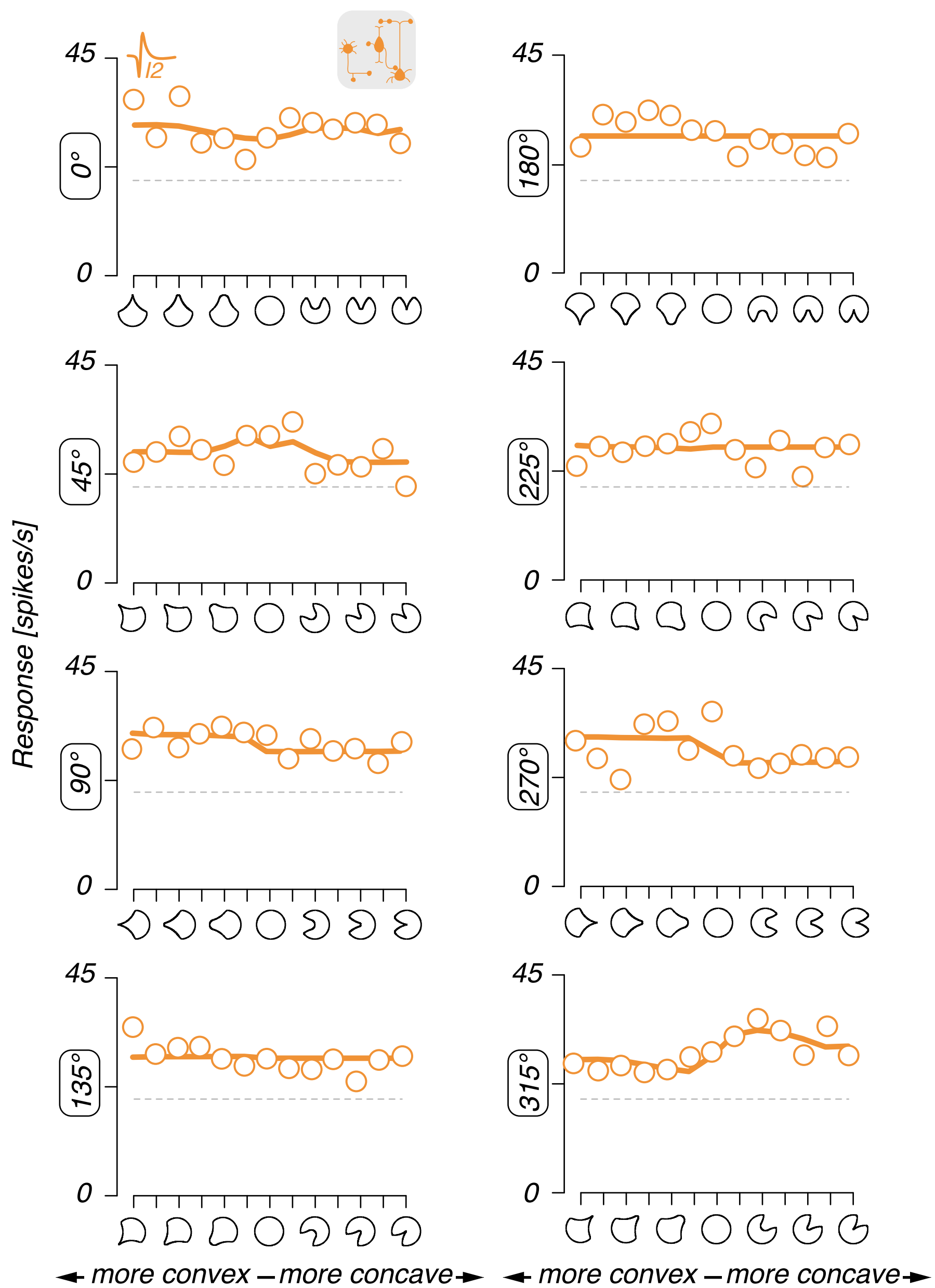

Supplementary Figure S7. Responses of an inhibitory neuron to shape stimuli presented at different rotations. Responses of an example neuron to all shape stimuli (13 shapes presented at 8 stimulus rotations; same data as in **Figure 4B**). Each subplot shows responses to stimuli at one stimulus rotation (0–315º in 45º steps). The neuron responded strongly and broadly to all stimulus rotations; the optimal stimulus rotation was 315º. Responses at all stimulus rotations were jointly fit by a quantitative model of shape selectivity (lines show model fit; dashed gray line shows spontaneous activity). The model fit provided a good account of the observed responses, demonstrating only weak selectivity for contour shape (shape selectivity index was 0.53).

SUPPLEMENTARY FIGURE S8

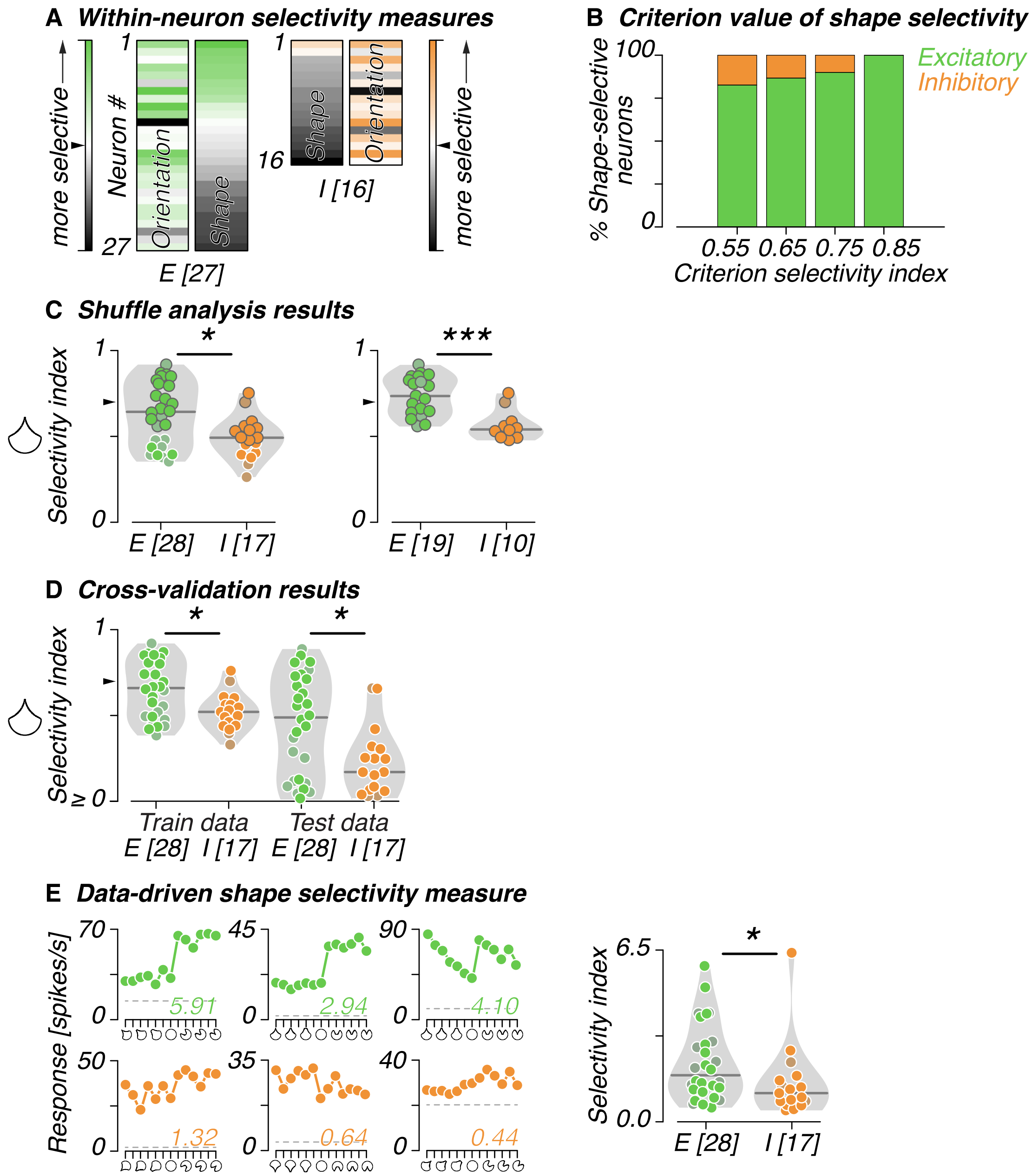

Supplementary Figure S8. Supporting analyses of visual form selectivity. (A) Within-neuron comparisons of selectivity measures for bar orientation and contour shape. Data are color coded by the degree of selectivity, with more saturated colors indicating higher selectivity above average criterion levels (arrowheads show criteria for selectivity); sample sizes are indicated. We did not find a significant correlation between orientation and shape selectivity measures for either cell type. **(B)** Percentage of neurons classified as shape-selective based on different criterion levels of the shape selectivity index. **(C)** Left: Distribution of shape selectivity indices for excitatory and inhibitory neurons (green and orange; arrowhead shows criterion for contour shape selectivity; index ≥ 0.7). Data points are color-coded based on the statistical significance determined by a shuffle analysis (gray outlines; $p \leq 0.05$). Data from neurons with short and long activation latencies are also color-coded (brighter and darker shades); sample sizes are indicated along the abscissa. Excitatory neurons had higher shape selectivity indices than inhibitory neurons ($p \leq 0.05$). Right: Distribution of significant shape selectivity indices for excitatory and inhibitory neurons based on the shuffle analysis ($p \leq 0.001$). **(D)** Distribution of cross-validated shape selectivity indices for excitatory and inhibitory neurons. The indices were computed separately for train and test data (80% train and 20% test; same format as in **B**; $p<0.05$). **(E)** Left: Responses of example neurons to stimuli presented at the optimal rotation. The data-driven shape selectivity index for each neuron is listed above its responses. This alternative measure captures how much of the observed responses modulate around the average firing rate. Right: Distribution of data-driven shape selectivity indices for excitatory and inhibitory neurons (same format as in **B**). Excitatory neurons had higher shape selectivity indices than inhibitory neurons ($p \leq 0.05$). Statistical comparisons were based on a Wilcoxon rank sum test.

SUPPLEMENTARY FIGURE S9

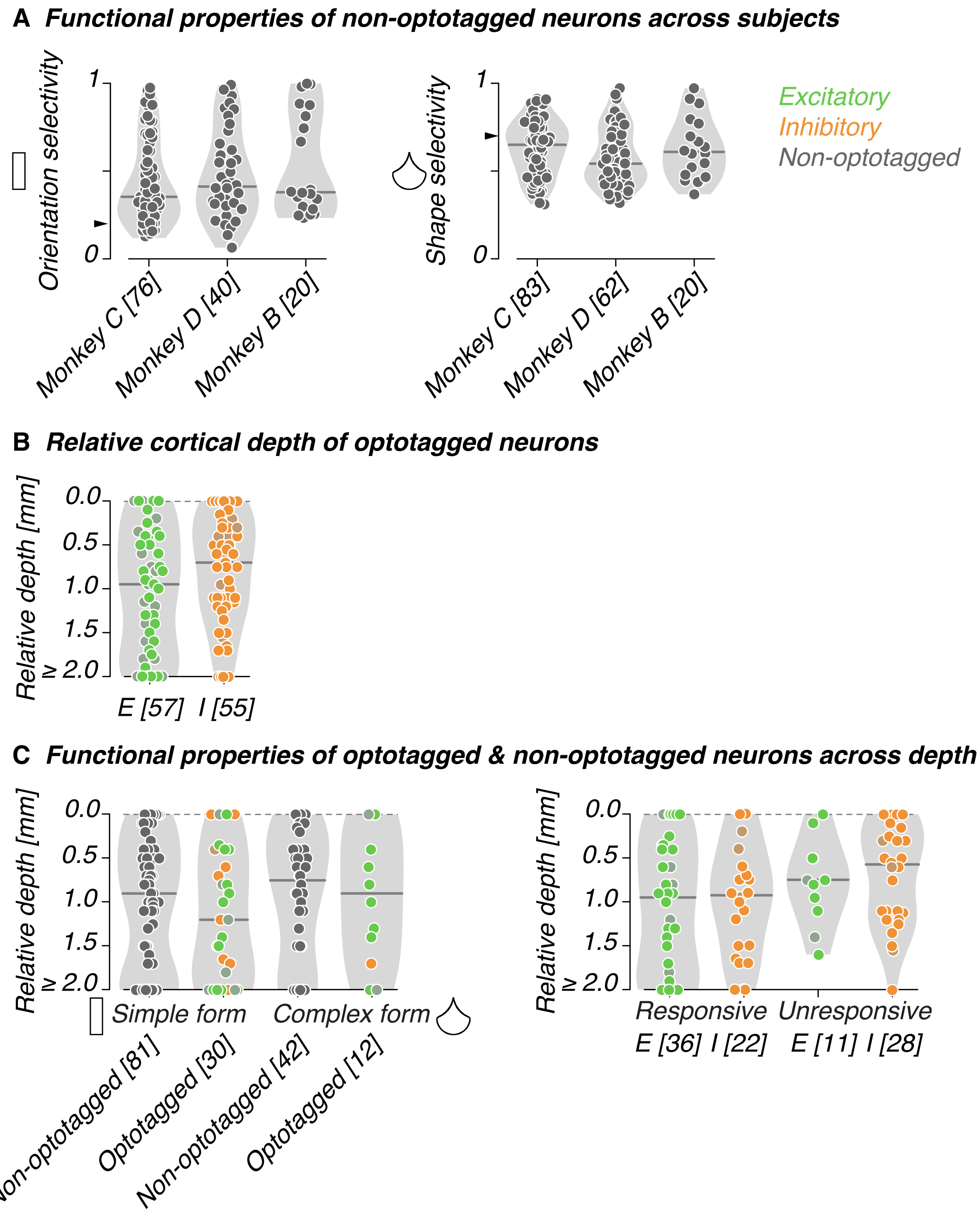

Supplementary Figure S9. Comparisons of optotagged and non-optotagged neurons across animals and cortical depth. (A) We recorded a collection of non-optotagged neurons (N=165; gray) in each of three macaques, at the same sites in which we recorded optotagged neurons (**Supplementary Table S2**). Left: Non-optotagged neurons had similar selectivity for simple forms (arrowhead shows criterion for selectivity); sample sizes are indicated along the abscissa. Right: non-optotagged neurons also had similar selectivity for complex forms. **(B)** Relative cortical depth of all optotagged excitatory and inhibitory neurons recorded (green and orange). Data from neurons with short and long activation latencies are color-coded (brighter and darker shades). We did not observe a clear gap at intermediate cortical depths corresponding to the absence of transduced neurons in the granular layer (see **Figure 1**), but this is likely due to the limited accuracy in estimating the position of brain surface in our experimental setup. **(C)** Functional comparisons of optotagged and non-optotagged neurons across depth. Left: Relative cortical depth of neurons that were selective for simple and complex forms. Right: Relative cortical depth of neurons that were visually responsive and unresponsive. Data from neurons with short and long activation latencies are color-coded (brighter and darker shades). We did not observe strong functional differences across cortical depth.

SUPPLEMENTARY FIGURE S10

A ***Receptive fields of optotagged excitatory neurons***

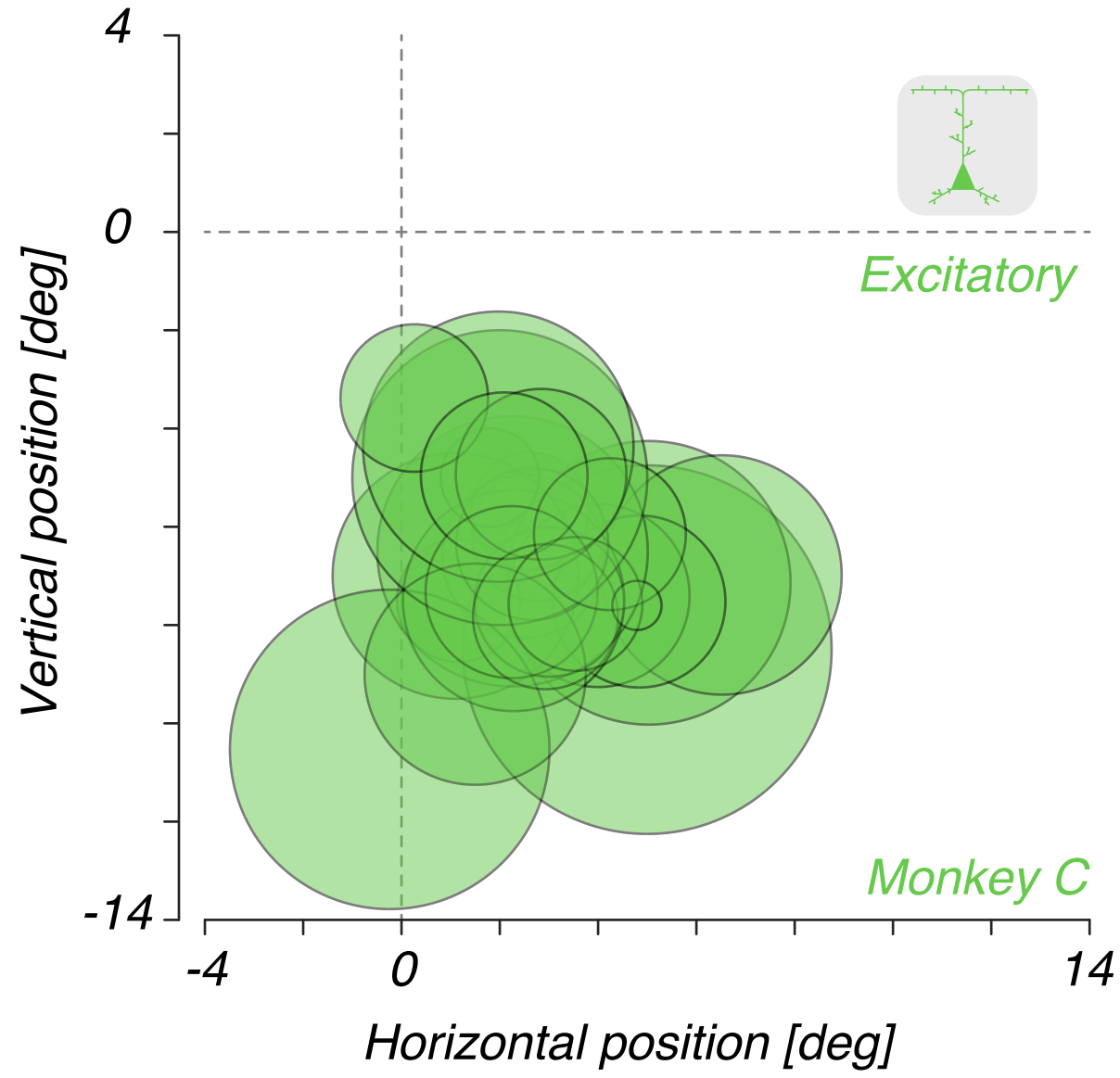

B ***Receptive fields of optotagged inhibitory neurons***

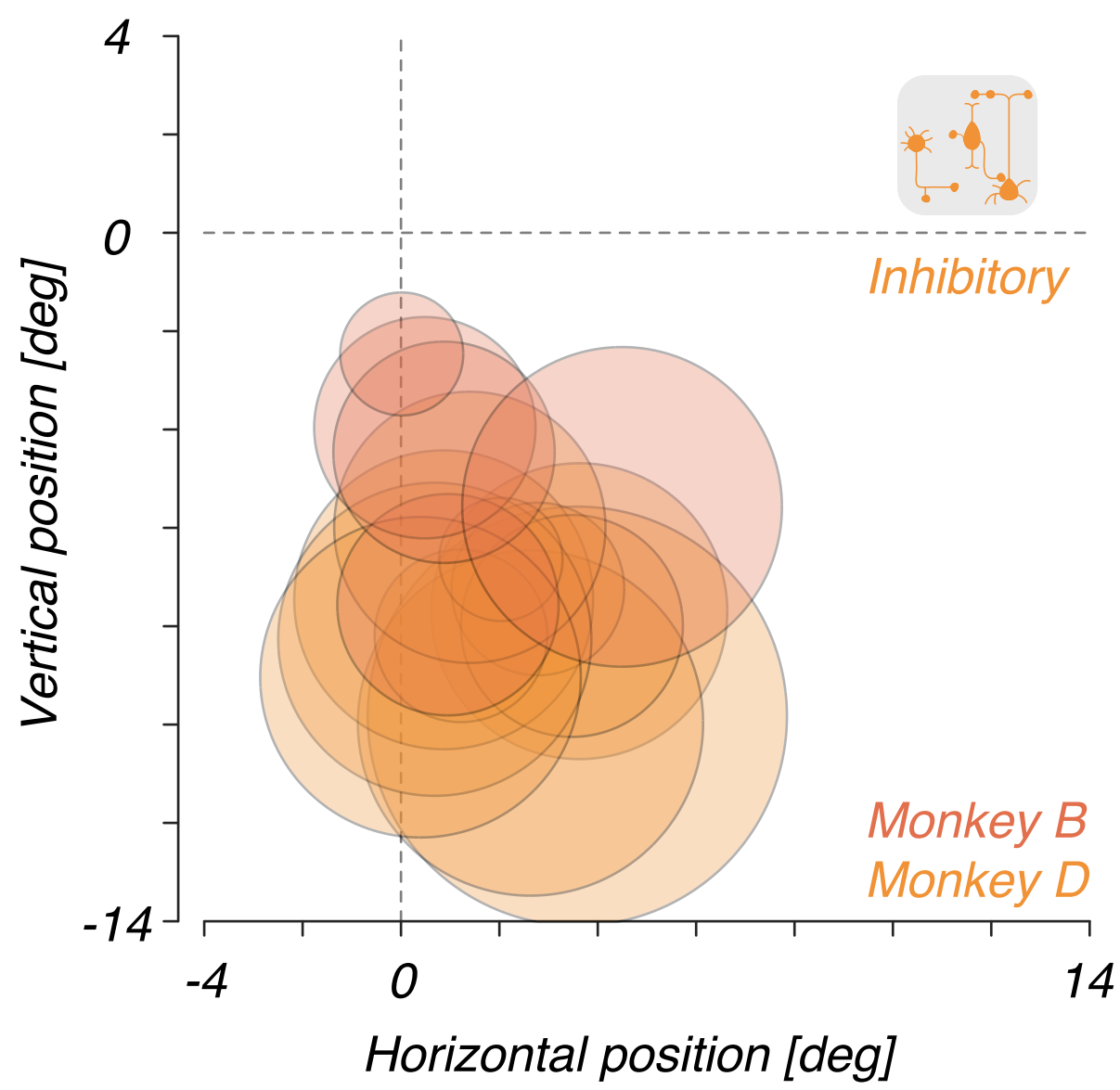

Supplementary Figure S10. Receptive fields of all visually responsive optotagged neurons. (A) Receptive field positions of all excitatory neurons (green; recorded in Monkey C). **(B)** Receptive field positions of all inhibitory neurons (orange; neurons recorded in Monkey B and Monkey D are shown in different shades). The size of each receptive field corresponds to the preferred stimulus size, determined quantitatively or qualitatively in separate mapping experiments. The receptive field eccentricities were similar for excitatory and inhibitory neurons (range was 2.5°–11.5° and median was 7.7° for E; range was 2.5°–11.5° and median was 7.8° for I). The preferred stimulus sizes were also similar for excitatory and inhibitory neurons (range was 0.5°–8.5° and median was 3.5° for E; range was 0.5°–8.5° and median was 4° for I). We did not find a significant correlation between receptive field eccentricities and measures of orientation and shape selectivity for either cell type.

SUPPLEMENTARY FIGURE S11

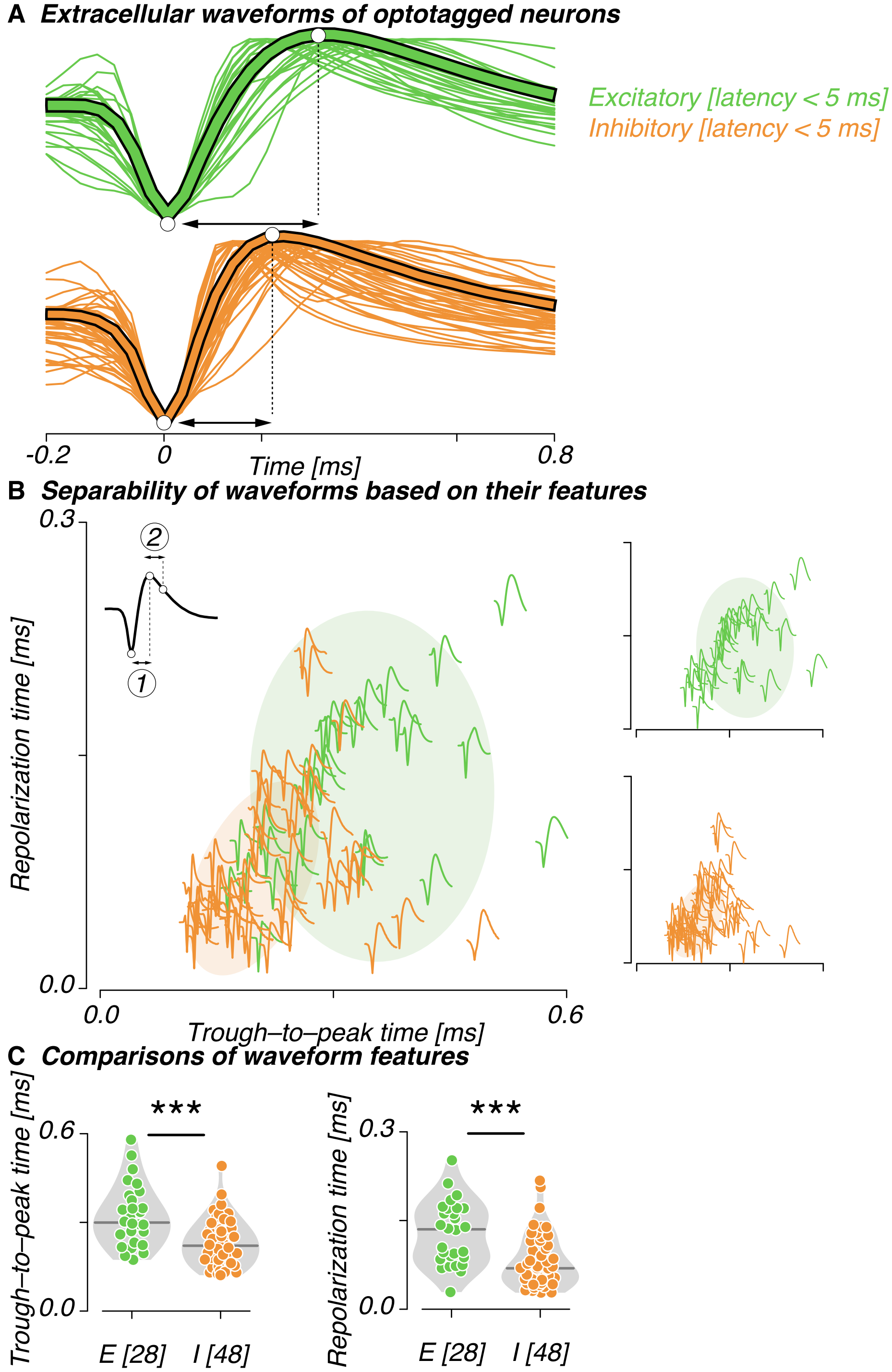

Supplementary Figure S11. Extracellular waveforms of excitatory and inhibitory neurons with short activation latencies. We repeated all comparisons between excitatory and inhibitory neurons, but this time including only neurons with activation latencies below 5 ms. **(A)** Average waveforms of all optotagged excitatory and inhibitory neurons (green and orange; thin lines are average waveforms for each neuron; thick lines are global averages for each neuronal cell type). **(B)** Separability of excitatory and inhibitory neuron waveforms. To compare the waveform properties of excitatory and inhibitory neurons, we computed two features: (i) trough–to–peak time and (ii) repolarization time (see inset). Left: Scatter plot of average waveforms for all optotagged neurons, sorted by their waveform features (data are color-coded by neuronal cell type and activation latency). Ellipses represent the output of a k-means clustering procedure to partition waveforms into two clusters; ellipses show two times the 95% confidence estimates around cluster centroids. Right: Scatter plots of average waveforms, shown separately for excitatory and inhibitory neurons. **(C)** Comparisons of waveform features for all optotagged neurons. Left: Distribution of trough–to–peak times ($p \leq 0.001$). Right: Distribution of repolarization times ($p \leq 0.001$). Sample sizes are indicated along the abscissa. Only waveforms with a dominant trough followed by a dominant peak contributed to the analysis of waveform features. Statistical comparisons were based on a Wilcoxon rank sum test.

SUPPLEMENTARY FIGURE S12

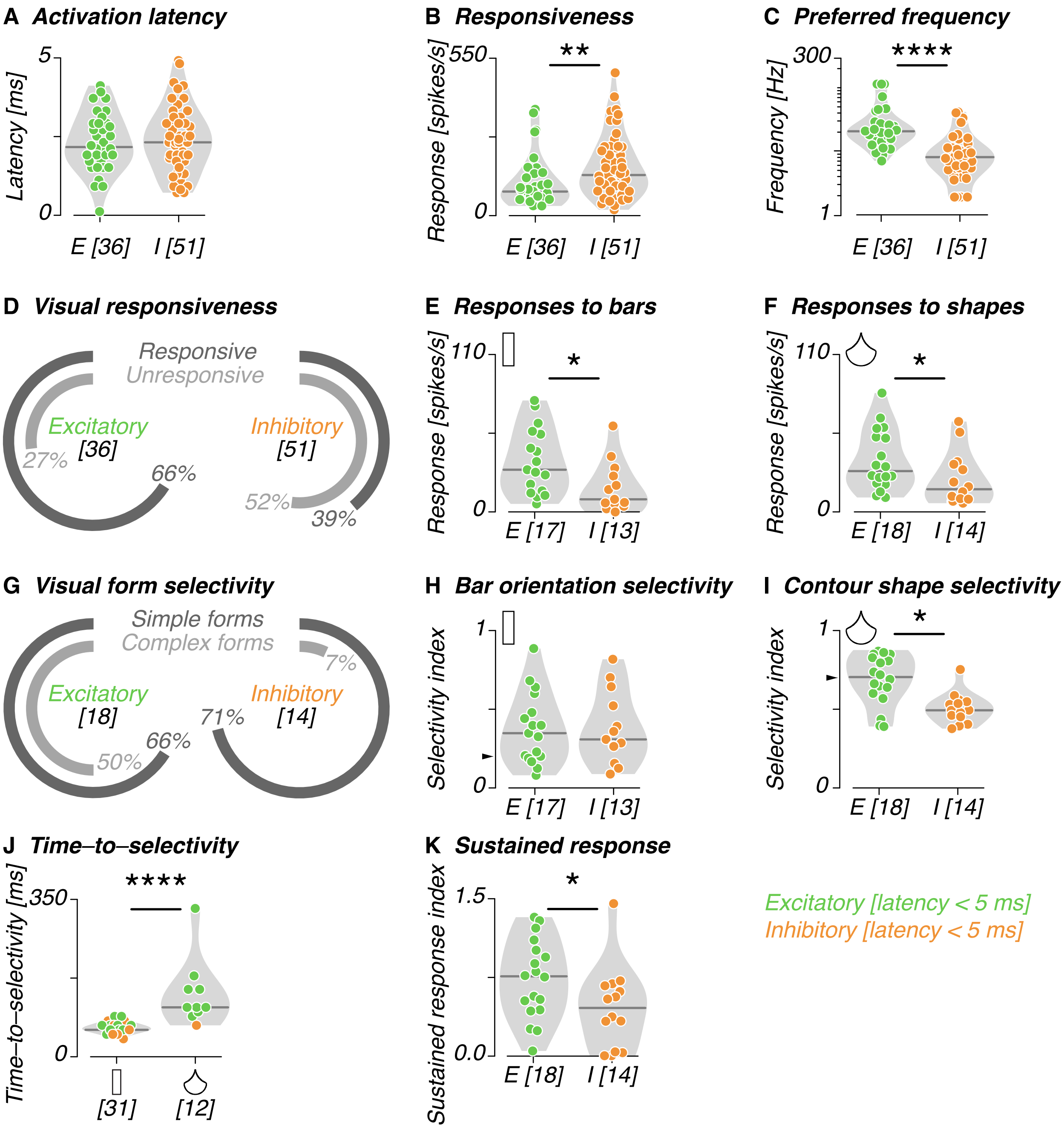

Supplementary Figure S12. Functional comparisons of excitatory and inhibitory neurons with short activation latencies. We repeated all comparisons between excitatory and inhibitory neurons, but this time including only neurons with activation latencies below 5 ms. **(A)** Distribution of activation latencies for all optotagged neurons. Data from excitatory and inhibitory neurons are shown separately (green and orange). Violin plots show marginal distributions (gray), and median values (gray lines). Sample sizes are indicated along the abscissa. **(B)** Distribution of peak responses to laser stimulation ($p \leq 0.01$). **(C)** Distribution of preferred laser modulation frequencies ($p \leq 0.0001$). **(D)** Proportion of visually responsive and unresponsive optotagged neurons (dark and light gray; some neurons were untested). **(E)** Distribution of peak responses to bar stimuli ($p \leq 0.05$). **(F)** Distribution of peak responses to shape stimuli ($p \leq 0.05$). **(G)** Proportion of visually responsive optotagged neurons selective for simple forms (i.e. bar orientation) and complex forms (i.e. contour shape; same format as in **D**). **(H)** Distribution of orientation selectivity indices (arrowhead shows criterion for bar orientation selectivity; index ≥ 0.2). **(I)** Distribution of shape selectivity indices ($p \leq 0.05$; arrowhead shows criterion for contour shape selectivity; index ≥ 0.7). **(J)** Distribution of time–to–selectivity measures for bar orientation and contour shape across all visually selective optotagged neurons ($p \leq 0.0001$). **(K)** Distribution of sustained response index ($p \leq 0.05$). Statistical comparisons were based on a Wilcoxon rank sum test.

SUPPLEMENTARY FIGURE S13

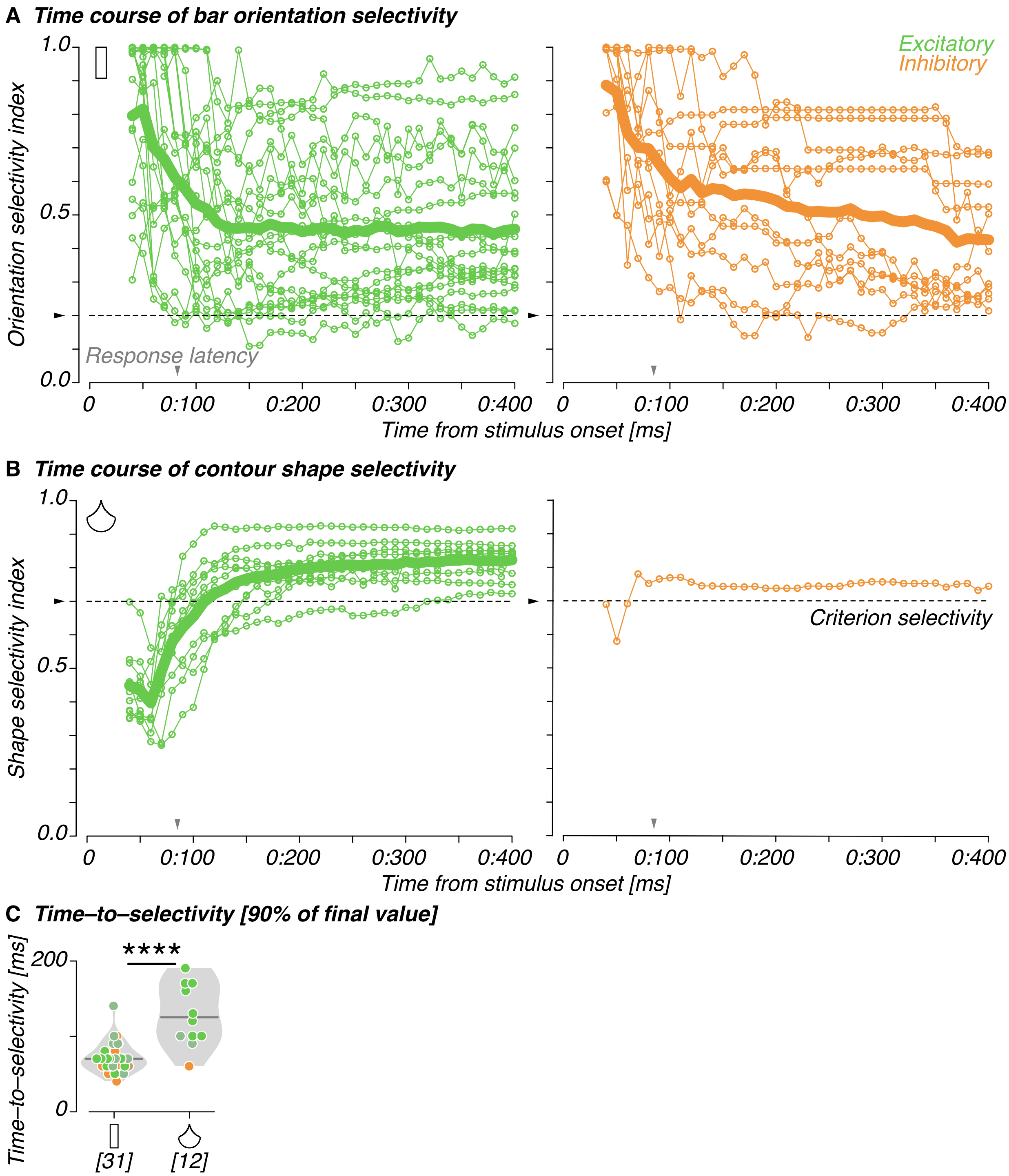

Supplementary Figure S13. Time course of visual form selectivity. We quantified neuronal selectivity for bar orientation and contour shape over progressively longer response epochs (shown along abscissa). **(A)** Time course of bar orientation selectivity for excitatory and inhibitory neurons (green and orange; thin lines are data for individual neurons; thick lines are the average; horizontal arrowhead and dashed line mark the criterion threshold for classification). Selectivity for bar orientation was typically highest in the earliest response epochs. **(B)** Time course of contour shape selectivity for excitatory and inhibitory neurons (same format as in **A**). Selectivity for contour shape was typically highest in the later response epochs. Only neurons that reached criterion levels of selectivity for either bar orientation or contour shape were included in this analysis. **(C)** Distribution of time–to–selectivity measures for neurons selective for bar orientation and contour shape ($p \leq 0.0001$; data are color-coded by neuronal cell type and activation latency). In this case, time–to–selectivity was defined as the first time point when the selectivity index reached 90% of its final value for two consecutive time bins. Statistical comparisons were based on a Wilcoxon rank sum test.

SUPPLEMENTARY FIGURE S14

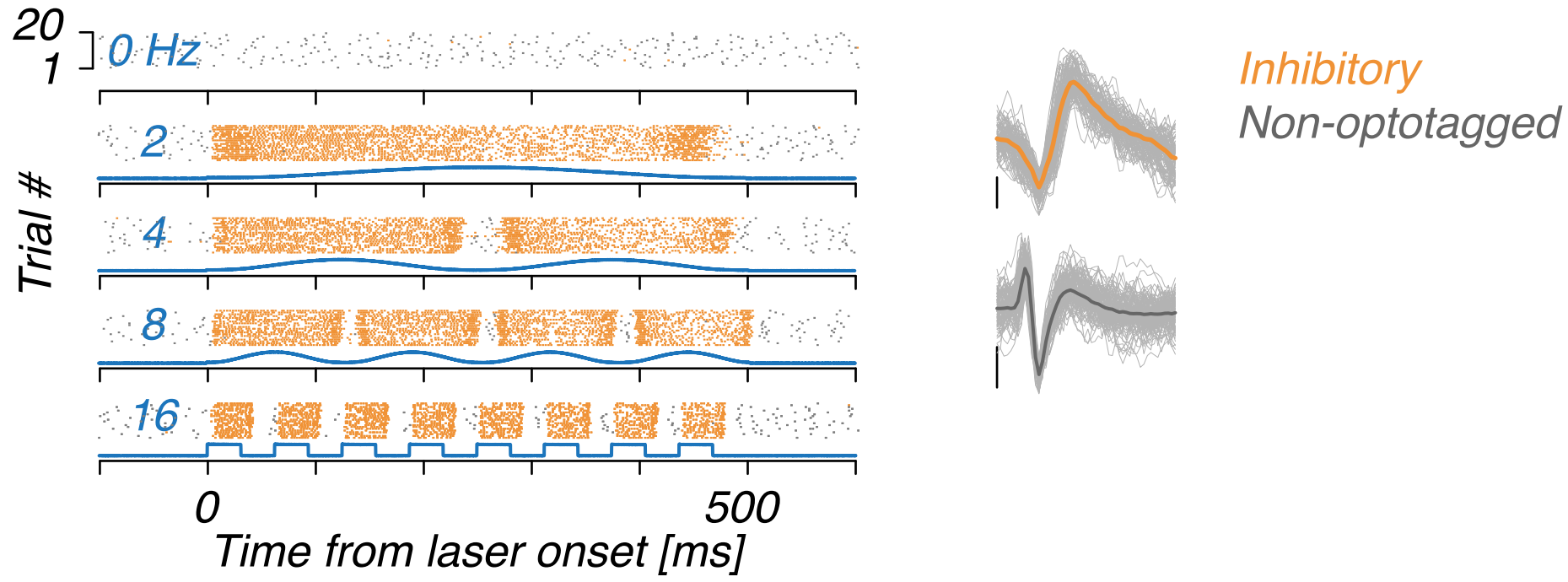

Supplementary Figure S14. Responses of an inhibitory neuron and a non-optotagged neuron recorded simultaneously. Left: Responses of an optotagged inhibitory neuron (orange) showing robust, short latency activation by blue light (activation latency was 3.0 ms). Also included are responses of a simultaneously recorded non-optotagged neuron (gray) showing suppression by blue light. Right: Extracellular waveforms of the inhibitory neuron and non-optotagged neuron. Scale bars under each waveform correspond to 10 μV.
