# Supplementary material for: Optotagging in primate cortex uncovers excitatory and inhibitory neuron specializations in form vision
Source: Nat Commun. 2026 Aug 26;17:10269. doi: 10.1038/s41467-026-76521-4 (PMC13616898; doi:10.1038/s41467-026-76521-4)
Supplement: Supplementary file 2 [file 41467_2026_76521_MOESM2_ESM.pdf]

nature portfolio

Corresponding author(s): Yasmine El-Shamayleh

Last updated by author(s): 07/13/2026

Reporting Summary

Nature Portfolio wishes to improve the reproducibility of the work that we publish. This form provides structure for consistency and transparency in reporting. For further information on Nature Portfolio policies, see our Editorial Policies and the Editorial Policy Checklist.

Statistics

For all statistical analyses, confirm that the following items are present in the figure legend, table legend, main text, or Methods section.

n/a	Confirmed	
☐	☒	The exact sample size (*n*) for each experimental group/condition, given as a discrete number and unit of measurement
☐	☒	A statement on whether measurements were taken from distinct samples or whether the same sample was measured repeatedly
☐	☒	The statistical test(s) used AND whether they are one- or two-sided<br>*Only common tests should be described solely by name; describe more complex techniques in the Methods section.*
☒	☐	A description of all covariates tested
☐	☒	A description of any assumptions or corrections, such as tests of normality and adjustment for multiple comparisons
☐	☒	A full description of the statistical parameters including central tendency (e.g. means) or other basic estimates (e.g. regression coefficient) AND variation (e.g. standard deviation) or associated estimates of uncertainty (e.g. confidence intervals)
☐	☒	For null hypothesis testing, the test statistic (e.g. *F*, *t*, *r*) with confidence intervals, effect sizes, degrees of freedom and *P* value noted<br>*Give P values as exact values whenever suitable.*
☒	☐	For Bayesian analysis, information on the choice of priors and Markov chain Monte Carlo settings
☒	☐	For hierarchical and complex designs, identification of the appropriate level for tests and full reporting of outcomes
☒	☐	Estimates of effect sizes (e.g. Cohen's *d*, Pearson's *r*), indicating how they were calculated

Our web collection on statistics for biologists contains articles on many of the points above.

Software and code

Policy information about availability of computer code

Data collection	Raw voltage signals (0.3Hz–7.5 kHz) were recorded using a neural recording system (Blackrock Neurotech, Cerebus, 64-channel).
Data analysis	Spike waveforms were detected by filtering the raw voltage signals (0.3–250 Hz) and extracting events that were 2.7 standard deviations above the noise (Plexon Systems, Offline Sorter). The waveforms of single neurons were then isolated manually using spike-sorting software (Plexon Systems, Offline Sorter). Further analyses on the data were conducted in MATLAB.<br><br>Code for data analysis is available in a public repository: https://doi.org/10.5281/zenodo.21342746

For manuscripts utilizing custom algorithms or software that are central to the research but not yet described in published literature, software must be made available to editors and reviewers. We strongly encourage code deposition in a community repository (e.g. GitHub). See the Nature Portfolio guidelines for submitting code & software for further information.

Data

Policy information about availability of data

All manuscripts must include a data availability statement. This statement should provide the following information, where applicable:

- Accession codes, unique identifiers, or web links for publicly available datasets
- A description of any restrictions on data availability
- For clinical datasets or third party data, please ensure that the statement adheres to our policy

Source data are provided, and the full dataset is available in a public repository: https://doi.org/10.5281/zenodo.21342746

Research involving human participants, their data, or biological material

Policy information about studies with human participants or human data. See also policy information about sex, gender (identity/presentation), and sexual orientation and race, ethnicity and racism.

Reporting on sex and gender	N/A
Reporting on race, ethnicity, or other socially relevant groupings	N/A
Population characteristics	N/A
Recruitment	N/A
Ethics oversight	N/A

Note that full information on the approval of the study protocol must also be provided in the manuscript.

Field-specific reporting

Please select the one below that is the best fit for your research. If you are not sure, read the appropriate sections before making your selection.

- [x] Life sciences
- [ ] Behavioural & social sciences
- [ ] Ecological, evolutionary & environmental sciences

For a reference copy of the document with all sections, see nature.com/documents/nr-reporting-summary-flat.pdf

Life sciences study design

All studies must disclose on these points even when the disclosure is negative.

Sample size	The study is based on data collected from 119 optotagged neurons: 59 excitatory neurons and 60 inhibitory neurons (Supplementary Table S2; 59 neurons from Monkey C; 19 from Monkey B and 41 from Monkey D). We also include data collected from 165 neurons recorded without knowledge of their cell type identity, referred to here as non-optotagged (Supplementary Table S2; 83 neurons from Monkey C; 20 neurons from Monkey B and 62 neurons from Monkey D). Non-optotagged neurons were sampled within the same cortical sites in which we recorded optotagged neurons.<br><br>To make functional comparison between excitatory and inhibitory neurons recorded across a limited number of animals, we strived to equate three major factors. First, we ensured similar visual response properties at the injection sites of all three animals (Supplementary Figure S8A). Second, we sampled a similar total number of optotagged excitatory and inhibitory neurons (E=59; I=60; Supplementary Table S2). Third, we sampled optotagged neurons within a similar area of V4 cortex (E=17.5 mm2; I =15.5 mm2; Supplementary Table S2).
Data exclusions	Our data inclusion criteria were three-fold. First, only well-isolated neurons were included. Second, for experiments characterizing the electrical responses of optotagged neurons, at least 8 repeats of each optical stimulation condition were collected (the median was 15 repeats; the range was 8–20 repeats). Third, for experiments characterizing the visual responses of optotagged neurons, at least 3 repeats of each visual stimulus condition were collected (the median was 7 repeats; the range was 3–20 repeats).
Replication	We required a specific number of repeated trials for each experimental condition. For experiments characterizing the electrical responses of optotagged neurons, at least 8 repeats of each optical stimulation condition were collected (the median was 15 repeats; the range was 8–20 repeats). For experiments characterizing the visual responses of optotagged neurons, at least 3 repeats of each visual stimulus condition were collected (the median was 7 repeats; the range was 3–20 repeats).<br>All attempts at replication were successful.
Randomization	Macaques were assigned randomly to the two experimental groups (vector injections targetting excitatory neurons and inhibitory neurons)
Blinding	N/A. It was not possible for investigators to be blinded to the optogenetic manipulations in this study because we needed to be aware of which viral vector was injected into which animal, and in which cortical location.

Reporting for specific materials, systems and methods

We require information from authors about some types of materials, experimental systems and methods used in many studies. Here, indicate whether each material, system or method listed is relevant to your study. If you are not sure if a list item applies to your research, read the appropriate section before selecting a response.

Materials & experimental systems

n/a	Involved in the study
	☒ Antibodies
☒	☐ Eukaryotic cell lines
☒	☐ Palaeontology and archaeology
	☒ Animals and other organisms
☒	☐ Clinical data
☒	☐ Dual use research of concern
☒	☐ Plants

Methods

n/a	Involved in the study
☒	☐ ChIP-seq
☒	☐ Flow cytometry
	☒ MRI-based neuroimaging

Antibodies

Antibodies used	Macaque cortical sections were processed using primary antibodies: chicken anti-GFP (Abcam 13970, 1:1000) and mouse anti-mCherry (Takara/Clontech 632543, 1:250), and rabbit anti-Parvalbumin (Swant PV27a, 1:1000). The sections were subsequently processed using secondary antibodies against: donkey anti-chicken Alexa Fluor 488 (Jackson Immuno Research 703-545-155, 1:400), donkey anti-mouse Alexa Fluor 568 (Invitrogen A10037, 1:5000), donkey anti-rabbit Alexa Fluor 488 (Invitrogen A21206, 1:500) and donkey anti-rabbit Alexa Fluor 568 (Invitrogen A10042, 1:500). Finally, the sections were processed with the nuclear stain DAPI (Invitrogen Molecular Probes D-21490, 1:10,000).
Validation	The antibodies were acquired from reputable commercial sources with appropriate certificates of analysis. All antibodies were validated experimentally for sensitivity in macaque cortical sections before use.

Animals and other research organisms

Policy information about studies involving animals; ARRIVE guidelines recommended for reporting animal research, and Sex and Gender in Research

Laboratory animals	Four healthy male rhesus macaque monkeys (Macaca mulatta) participated in the study (8–13 years).
Wild animals	N/A
Reporting on sex	Only male macaques were used.
Field-collected samples	N/A
Ethics oversight	All aspects of animal care, surgical procedures and experimental protocols conformed to the NIH Guide for the Care and Use of Laboratory Animals, and were approved by the Institutional Animal Care and Use Committee at Columbia University.

Note that full information on the approval of the study protocol must also be provided in the manuscript.

Plants

Seed stocks	N/A
Novel plant genotypes	N/A
Authentication	N/A

Magnetic resonance imaging

Experimental design

Design type	*Indicate task or resting state; event-related or block design.*
Design specifications	*Specify the number of blocks, trials or experimental units per session and/or subject, and specify the length of each trial or block (if trials are blocked) and interval between trials.*
Behavioral performance measures	*State number and/or type of variables recorded (e.g. correct button press, response time) and what statistics were used to establish that the subjects were performing the task as expected (e.g. mean, range, and/or standard deviation across subjects).*

Acquisition

Imaging type(s)	*Specify: functional, structural, diffusion, perfusion.*
Field strength	*Specify in Tesla*
Sequence & imaging parameters	*Specify the pulse sequence type (gradient echo, spin echo, etc.), imaging type (EPI, spiral, etc.), field of view, matrix size, slice thickness, orientation and TE/TR/flip angle.*
Area of acquisition	*State whether a whole brain scan was used OR define the area of acquisition, describing how the region was determined.*

Diffusion MRI ☐ Used ☐ Not used

Preprocessing

Preprocessing software	*Provide detail on software version and revision number and on specific parameters (model/functions, brain extraction, segmentation, smoothing kernel size, etc.).*
Normalization	*If data were normalized/standardized, describe the approach(es): specify linear or non-linear and define image types used for transformation OR indicate that data were not normalized and explain rationale for lack of normalization.*
Normalization template	*Describe the template used for normalization/transformation, specifying subject space or group standardized space (e.g. original Talairach, MNI305, ICBM152) OR indicate that the data were not normalized.*
Noise and artifact removal	*Describe your procedure(s) for artifact and structured noise removal, specifying motion parameters, tissue signals and physiological signals (heart rate, respiration).*
Volume censoring	*Define your software and/or method and criteria for volume censoring, and state the extent of such censoring.*

Statistical modeling & inference

Model type and settings	*Specify type (mass univariate, multivariate, RSA, predictive, etc.) and describe essential details of the model at the first and second levels (e.g. fixed, random or mixed effects; drift or auto-correlation).*
Effect(s) tested	*Define precise effect in terms of the task or stimulus conditions instead of psychological concepts and indicate whether ANOVA or factorial designs were used.*

Specify type of analysis: ☐ Whole brain ☐ ROI-based ☐ Both

Statistic type for inference (See Eklund et al. 2016)	*Specify voxel-wise or cluster-wise and report all relevant parameters for cluster-wise methods.*
Correction	*Describe the type of correction and how it is obtained for multiple comparisons (e.g. FWE, FDR, permutation or Monte Carlo).*

Models & analysis

n/a	Involved in the study
☐	☐ Functional and/or effective connectivity
☐	☐ Graph analysis
☐	☐ Multivariate modeling or predictive analysis

Functional and/or effective connectivity	*Report the measures of dependence used and the model details (e.g. Pearson correlation, partial correlation, mutual information).*
Graph analysis	*Report the dependent variable and connectivity measure, specifying weighted graph or binarized graph, subject- or group-level, and the global and/or node summaries used (e.g. clustering coefficient, efficiency, etc.).*

Multivariate modeling and predictive analysis | *Specify independent variables, features extraction and dimension reduction, model, training and evaluation metrics.*
