# Supplementary material for: Optotagging in primate cortex uncovers excitatory and inhibitory neuron specializations in form vision
Source: Nat Commun. 2026 Aug 26;17:10269. doi: 10.1038/s41467-026-76521-4 (PMC13616898; doi:10.1038/s41467-026-76521-4)
Supplement: Supplementary file 3 [file 41467_2026_76521_MOESM3_ESM.pdf]

nature portfolio

Peer Review File

Optotagging in primate cortex uncovers excitatory and inhibitory neuron specializations in form vision

Corresponding Author: Dr Yasmine El-Shamayleh

This file contains all reviewer reports in order by version, followed by all author rebuttals in order by version.

Version 0:

Reviewer comments:

Reviewer #1

(Remarks to the Author)
Su and El-Shamayleh use an optogenetic tagging approach, originally developed in rodents, to identify and functionally characterize excitatory and inhibitory neurons in macaque visual area V4. By combining cell-type identification with responses to both oriented bars and complex (contour) shapes, the authors provide a very rare and valuable characterization of cell-type-specific response properties in a higher-order visual area of the primate cortex.
The study reveals clear functional differences between excitatory and inhibitory neurons. By and large, many inhibitory neurons were visually unresponsive; those that were responsive showed some orientation selectivity, but, unlike excitatory V4 neurons, exhibited little to no selectivity for complex shapes. In contrast, shape selectivity in excitatory neurons emerged at longer time scales than orientation selectivity and was associated with more sustained responses. These results strongly support the hypothesis that excitatory neurons play a prominent role in the development of shape selectivity in area V4, at least under passive viewing conditions.
This is an exceedingly challenging and very well-executed study that yields important insights into cell-type-specific mechanisms underlying shape processing in the visual system of nonhuman primates. Both technically and conceptually, the work sets the stage for future studies using similar approaches to elucidate the contributions of distinct cell types to higher cognitive functions in primates.
The manuscript is detailed, clearly written, and a genuine pleasure to read. The analytical approaches are sophisticated, appropriate, and carefully applied. Although particularly critical readers may note that the number of optotagged neurons is relatively modest (entirely expected given the technical challenges of this approach) the reported effects appear robust and reproducible. I appreciate the use of multiple complementary analytical approaches .In summary, this reviewer is highly enthusiastic about the work and strongly supports publication. I have only a small number of minor comments that the authors may wish to consider to further improve the manuscript.

Minor Comments
1. Introduction, second-to-last paragraph: I suggest revising the wording of “poor visual activity in mice make them unsuitable…”. While the point is understood, a more neutral phrasing would avoid unnecessarily alienating rodent researchers.
2. Introduction, last paragraph: The statement that shape selectivity is “a property likely computed within cortical area V4” may be too strong. While longer processing times are clearly required, the data do not rule out contributions from feedback or recurrent processing involving other areas. A more cautious phrasing may be appropriate.
3. I may have missed this, but what was the rationale for using both square-wave and sine-wave laser stimulation?
4. I appreciate the demonstration of the weak correspondence between optotagged cell-type identification and waveform-based classification. This result underscores how unreliable waveform-based classification can be at the single-cell level (i.o.w., except for neurons at the tails of the distribution, it is impossible to assess whether a neuron is inhibitory or excitatory) . A formal cross-validation analysis could further quantify this limitation. Regardless, this technical point deserves stronger emphasis in the discussion, and possibly even in the abstract, if space permits.
5. If I understood correctly, only one optotagged inhibitory neuron showed shape selectivity. Given that Dlx specificity is not perfect, it seems conceivable that this neuron may have been misclassified. This possibility may be worth briefly mentioning?
6. Figure 1 shows that layer 4 contains the fewest transduced neurons (for both Dlx5/6 and CaMKII), consistent with previous reports. Given this, I was surprised by the apparently homogeneous distribution of optotagged neurons as a function of cortical depth (Fig. S8). In this context, the statement on page 8 (“…consistent with our histological analyses…”) does not

seem fully justified. Do the authors have an explanation for this discrepancy, beyond the inherent difficulty of assigning precise laminar locations during electrophysiological recordings with single contact points?
7. Figure 7C and D (Page 10): The final result described here is anecdotal but highly intriguing. It is reminiscent of behavioral findings showing that optogenetic stimulation of FEF neurons primarily evokes saccades (only) when combined with low-amplitude electrical stimulation (J Neurosci, 2013; DOI:10.1523/JNEUROSCI.2675-13.2013). It is also consistent with evidence that FEF microstimulation strongly modulates visual cortical activity only in the presence of a retinal stimulus (Nature, 2003; Science, 2007). This connection might be worth briefly noting.
8. Page 11, second paragraph: The claim that contour shape selectivity emerges over time from orientation-selective inputs is not directly supported by the data. While this interpretation is plausible, other mechanisms, some of which are already listed, may be more important. A more cautious phrasing is recommended.
9. Page 14: Injection depth: The Methods state that injections were made throughout the depth of gray matter (~5 mm). Given that macaque neocortex is approximately 2 mm thick (or less), does this imply that injections extended beyond the prelunate gyrus (e.g., into the STS or lunate sulcus)?
10. The laser power used is relatively high. Although thermal artifacts seem unlikely, can they be completely excluded? In principle, such effects could differentially influence excitatory and inhibitory neurons.
11. Were non-optotagged neurons recorded in regions where viral injections were performed (i.e., neurons that were simply not transduced), or were these recordings obtained from cortical sites without viral expression?
12. I may have missed this, but what criterion was used to classify a neuron as visually responsive?

Reviewer #2

(Remarks to the Author)
In their manuscript titled ‘Optotagging in primate cortex uncovers excitatory and inhibitory neuron specializations in form vision’, Chuyi Su and Yasmine El-Shamayleh demonstate an optogenetic approach in macaque V4 to directly dissociate excitatory and inhibitory neuron contributions to visual form processing. The authors demonstrate the technical feasibility of cell-type specific viral targeting and in vivo optical identification in primate cortex, which represents an important methodological step for circuit-level investigations in non-human primates. Functionally, the data show that excitatory neurons are more visually responsive and exhibit stronger contour shape selectivity than inhibitory neurons, which appear to primarily encode simple orientation information. The temporal analyses further suggest delayed emergence of contour shape selectivity relative to orientation tuning and differences in response dynamics across cell types. These findings clarify how form information may be distributed across cortical cell types and highlight the promise of optogenetic tagging for computational modeling and causal circuit analysis in primates.
While the study is technically ambitious and addresses an important question, several aspects of the analyses and interpretations require clarification and more cautious framing. I outline these major concerns below.

Major comments
1) The histological validation (figure 1C–D) was performed in a different animal (Monkey R), whereas neurophysiology was conducted in Monkeys C, B, and D. While the images suggest cell-type specificity, the localization of ChR2 in the soma of V4 excitatory neurons appears relatively sparse compared to inhibitory expression. There is no quantification of ChR2-eYFP expression relative to CaMKII-positive cells, making it unclear what fraction of excitatory neurons were successfully transduced. Although sparse expression may not compromise light-evoked control of optotagged neurons’ spiking for identification purposes, it raises concerns about sampling bias. If only a subset of pyramidal neurons expresses ChR2, the optotagged excitatory population may not represent the broader excitatory pool in V4. This is particularly relevant given the central claim that excitatory neurons preferentially encode complex contour shape information. Supplementary Table S2 also suggests relatively low optotagged yield compared to non-tagged neurons across animals. Quantification of transduction efficiency (e.g., ChR2+/CaMKII+ ratios) and clarification of expression consistency across monkeys would substantially strengthen the claims of cell-type specificity and generalizability of the findings (e.g., how effectively this approach could manipulate excitatory populations to probe V4-mediated perception at the circuit level).

2) This laminar bias of ChR2 expression, i.e., little to no expression in the granular layer, might significantly limit interpreting the results. In primate V4, layer 4 receives strong feedforward input from earlier visual areas (e.g., V2) and plays a key role in shaping orientation and contour selectivity before information is distributed to superficial and deep layers. If excitatory and inhibitory neurons in layer 4 are underrepresented or unlabeled, the optotagged population may not fully capture the feedforward circuitry contributing to visual form processing. This limitation could constrain the ability to functionally dissociate cell-type contributions across cortical microcircuits, particularly when interpreting differences in shape selectivity and response dynamics. For example, conclusions about inhibitory neurons signaling primarily simple features may reflect sampling bias away from specific laminar subpopulations rather than a generalized property of V4 inhibitory neurons. Clarifying laminar expression patterns and recording depth distributions relative to layer boundaries would strengthen the cell-type specific interpretation of the findings.

3) While the histological images suggest broad excitatory and inhibitory targeting, the manuscript does not provide quantitative immunohistochemical characterization of which inhibitory subtypes were transduced. The mDlx5/6 enhancer labels GABAergic neurons broadly and does not distinguish among major interneuron classes (e.g., PV+, SST+, VIP+), which are known to differ substantially in connectivity and functional roles. Without subtype-specific validation, it remains unclear how representative the optotagged inhibitory population is with respect to known molecular classes. This limitation becomes particularly relevant where functional differences between fast-spiking and regular-spiking neurons are discussed (Figure 7). Because waveform-based classification does not map cleanly onto molecular identity in primate cortex,

interpretations regarding inhibitory specialization should be framed cautiously.

4) The central claim that excitatory neurons preferentially encode complex contour shape information. While the categorical difference in shape-selective neurons (37% E vs 6% I) is striking, the median contour shape selectivity indices differ more modestly (0.64 vs 0.50), suggesting that the sharp dissociation depends in part on the stringent ≥0.7 classification threshold. This criterion is adopted from previous V4 studies (Pasupathy & Connor, 2001; El-Shamayleh & Pasupathy, 2016), which were based primarily on extracellular recordings from putative pyramidal neurons. While appropriate for excitatory-dominated datasets, its applicability to inhibitory neurons, whose tuning properties, firing rates, and response variance may differ requires explicit justification. Applying the same threshold across cell types assumes comparable tuning structure, which may not hold for interneurons. It would strengthen the manuscript to demonstrate that the reported cell-type differences remain robust across a range of thresholds and to frame conclusions in terms of distributional shifts rather than categorical exclusivity.

5) The time-to-selectivity analysis relies on fixed classification thresholds (≥0.2 for orientation; ≥0.7 for shape) to define the moment at which tuning emerges. While these thresholds are justified for classifying selectivity in prior V4 studies, using them to estimate latency may introduce bias, particularly when comparing two indices with different dynamic ranges and distributions. Because shape selectivity is defined by a more stringent criterion and a higher-dimensional model, part of the observed delay in contour shape selectivity could reflect threshold crossing dynamics rather than intrinsic computational timing. It would strengthen the conclusions to show the full temporal evolution of both selectivity indices and/or to estimate latency using a more unbiased approach (e.g., normalized growth or statistical deviation from baseline), thereby isolating genuine temporal differences in tuning emergence.

6) The authors conclude that visually unresponsive inhibitory neurons require coincident inputs to become active, based on supralinear responses during combined optical and visual stimulation. However, this conclusion appears based on a small number of example neurons and lacks clear population-level quantification. It remains unclear how robust or prevalent this effect is across the inhibitory population. Additionally, subtype classification based solely on waveform width (fast- vs regular-spiking) should be interpreted cautiously in primate cortex, where extracellular waveform does not map cleanly onto molecular identity. The coincidence-gating interpretation should therefore be framed more conservatively.

Minor Comments
1) Although the authors provide supplementary analyses restricted to short-latency (<5 ms) neurons (supplementary figure S10 and S11) and report similar results, the main figures include excitatory neurons with longer activation latencies that may reflect indirect activation. Because inhibitory neurons do not face the same ambiguity, this introduces asymmetry in classification confidence across cell types. Given that the central claims rely on clean cell-type dissociation, it would strengthen the manuscript to either (i) present the <5 ms analyses in the main figures or (ii) more prominently demonstrate that all key statistical comparisons are robust under the stricter latency criterion.
2) Figure 1D legend: “ChR2-positive neurons are in red; nuclei stained by DAPI are in blue” appears twice.

Reviewer #3

(Remarks to the Author)
In the current study, the authors use cell type-selective optogenetic tools to identify excitatory and inhibitory neurons in primate V4. Although optotagging in vivo has been used in rodent models for quite some time, the use of this approach has lagged in non-human primate models due to lack of cell type-specific viral tools. Here the authors use a CAMKII promoter and the dlx enhancer to drive expression broadly in excitatory and inhibitory cells, and then further subdivide the inhibitory interneurons based on action potential waveform. They examine the visual selectivity of these classes for the first time. Overall, this is a nice demonstration of the technical approach, and the authors identify some intriguing differences between the two groups. There are three general areas where the manuscript could be improved:

The study would benefit from digging a little more creatively into the dataset. The authors obviously have population recordings of non-tagged cells during stimulation of either excitatory or inhibitory neurons. Does activating interneurons reduce non-tagged cell firing, as expected? Does activating excitatory neurons recruit non-tagged cells as expected? According to the methods, the authors recorded broadband signals, meaning they also have field potential recordings. Do interneurons show selective spike-field coupling to high frequencies? The authors could do more with the dataset they have to test whether the commonly observed relationships between excitatory and inhibitory neurons and local populations/LFPs are the same for higher-order cortex as for primary sensory areas.

The authors’ treatment of the existing literature on inhibitory interneurons is disappointingly thin and should be expanded to provide a richer context for the current findings. At this point, most of what we know about the sensory response properties of GABAergic interneurons comes from the extensive rodent literature, and the authors should put their findings in the context of that knowledge. The authors’ entire argument about their findings rests on their claim that interneurons in rodents are tuned, making the lack of selectivity in the primate surprising. In fact, most studies, including those in cat V1, make a strong case for a lack of selectivity in interneurons across cell types. In cat V1, FS cells are significantly less selective than RS cells across all layers. In mouse, PV and SST interneurons in V1 and S1 are less tuned, and in some cases entirely untuned, for visual stimulus features such as orientation. PV cells are more tuned than SST and VIP cells for spatial frequency, but less tuned than pyramidal neurons (see DiPoppa et al., 2018, Millman et al., 2020, deVries et al 2019, etc). The single exception may be in the domain of visual contrast, where most existing data suggests equivalent contrast sensitivity for excitatory and inhibitory cells (but see Millman et al., for VIP contrast responses). Although no data exist on interneurons tuning for higher-

order features in rodents, the overall bulk of the existing data suggest that interneurons are not as selective as excitatory neurons. The authors' current findings thus generally fit well with previous literature.

One aspect where the authors' results are quite surprising is the relatively modest percentage of interneurons that show sensory responses. The authors develop this portion of the study using optogenetic depolarization to enhance the responses of these cells. It is not accurate to call these cells 'silent', since they could not be identified unless they were periodically active, and the authors should use a different label. Nevertheless, these results are very intriguing, given the consensus model of cortical circuit function where input to a circuit always evokes yoked excitation and inhibition, with excitation and inhibition always being fairly well balanced. In contrast, the current manuscript makes a case for considerably less inhibition than excitation being evoked in response to a driving stimulus in V4. The authors should flesh out how the low rate of sensory-evoked inhibitory firing does or does not violate the canonical feedforward E-I model.

Version 1:

Reviewer comments:

Reviewer #1

(Remarks to the Author)
The authors have done an excellent job with their revised manuscript. I particularly appreciate their careful approach to acknowledging limitations rather than overclaiming when results on certain aspects remain partly inconclusive. This intellectual honesty strengthens rather than weakens the work. We shoudl not forget that Rome is not built on one day... The study itself is exceedingly valuable, establishing a foundation for future research in this area of opto-tagging in NHP. I highly recommend this manuscript for publication in Nature Communications and commend the authors!

Reviewer #2

(Remarks to the Author)
In their revised manuscript, the authors have addressed the major concerns raised in my original comments through additional analyses, new supplementary figures, and more cautious interpretation of several key findings. While some methodological limitations remain inherent to the dataset (e.g., quantification of transduction efficiency, laminar coverage, and inhibitory subtype specificity), these limitations are now appropriately acknowledged and discussed. I have no major remaining concerns and support publication after minor revisions.

Minor comments
1. The additional analyses substantially strengthen the conclusions regarding temporal dynamics. Although a normalized or statistical-onset latency measure would provide a more criterion-independent estimate of tuning emergence, I do not consider this essential for publication.
2. Typo, page 5: 'The functional similarities across animals demonstrates…': demonstrate.
3. Typo, page 9: 'we reasoned that a sensible proxy would be to examined…': examine
4. Typo, page 10: 'These findings suggest that information about bar orientation may already present…': may already present
5. Page 10: This section is redundant, 'Similarly, we also recorded optotagged neurons that were visually responsive and unresponsive neurons across cortical depth…'

Reviewer #3

(Remarks to the Author)
The authors have largely addressed my concerns, I think this paper is a nice addition to the modern primate cortical literature.

If I may make a suggestion for the future, I strongly encourage the authors to think more broadly about capturing and analyzing both units and fields in their datasets, particularly when it is possible to identify cell classes using optotagging. It would be enormously valuable for the field to have more extensive primate cortical electrophysiological datasets with both multiple single units and field potentials.

Open Access This Peer Review File is licensed under a Creative Commons Attribution 4.0 International License, which permits use, sharing, adaptation, distribution and reproduction in any medium or format, as long as you give appropriate credit to the original author(s) and the source, provide a link to the Creative Commons license, and indicate if changes were made.
In cases where reviewers are anonymous, credit should be given to 'Anonymous Referee' and the source.
The images or other third party material in this Peer Review File are included in the article's Creative Commons license, unless indicated otherwise in a credit line to the material. If material is not included in the article's Creative Commons license and your intended use is not permitted by statutory regulation or exceeds the permitted use, you will need to obtain permission directly from the copyright holder.
To view a copy of this license, visit https://creativecommons.org/licenses/by/4.0/

RESPONSE TO REVIEWERS

NCOMMS-26-000342-T

Optotagging in primate cortex uncovers excitatory and inhibitory neuron specializations in form vision

Chuyi Su & Yasmine El-Shamayleh

We are very grateful to the reviewers for their careful evaluation of our manuscript and helpful comments. We are also pleased that all three reviewers valued the technical and conceptual advances enabled by our study. Guided by their collective feedback, we now submit a revision that incorporates several new analyses, figures and improved text. Below, please find our responses (in blue) to each of the reviewers' comments (in black).

Reviewer #1

Su and El-Shamayleh use an optogenetic tagging approach, originally developed in rodents, to identify and functionally characterize excitatory and inhibitory neurons in macaque visual area V4. By combining cell-type identification with responses to both oriented bars and complex (contour) shapes, the authors provide a very rare and valuable characterization of cell-type-specific response properties in a higher-order visual area of the primate cortex. The study reveals clear functional differences between excitatory and inhibitory neurons. By and large, many inhibitory neurons were visually unresponsive; those that were responsive showed some orientation selectivity, but, unlike excitatory V4 neurons, exhibited little to no selectivity for complex shapes. In contrast, shape selectivity in excitatory neurons emerged at longer time scales than orientation selectivity and was associated with more sustained responses. These results strongly support the hypothesis that excitatory neurons play a prominent role in the development of shape selectivity in area V4, at least under passive viewing conditions.

This is an exceedingly challenging and very well-executed study that yields important insights into cell-type-specific mechanisms underlying shape processing in the visual system of nonhuman primates. Both technically and conceptually, the work sets the stage for future studies using similar approaches to elucidate the contributions of distinct cell types to higher cognitive functions in primates.

The manuscript is detailed, clearly written, and a genuine pleasure to read. The analytical approaches are sophisticated, appropriate, and carefully applied. Although particularly critical readers may note that the number of optotagged neurons is relatively modest (entirely expected given the technical challenges of this approach) the reported effects appear robust and reproducible. I appreciate the use of multiple complementary analytical approaches .In summary, this reviewer is highly enthusiastic about the work and strongly supports publication. I have only a small number of minor comments that the authors may wish to consider to further improve the manuscript.

We thank Reviewer #1 for the constructive feedback, including several excellent suggestions for improved phrasing and additional analyses that have greatly strengthened the revised manuscript. We are also pleased that the reviewer recognizes our work as "an exceedingly challenging and very well executed study that yields important insights" and that "sets the stage for future studies…to elucidate the contributions of distinct cell types to higher cognitive functions in primates".

Minor Comments

1. Introduction, second-to-last paragraph: I suggest revising the wording of "poor visual activity in mice make them unsuitable...". While the point is understood, a more neutral phrasing would avoid unnecessarily alienating rodent researchers.

We greatly value this perspective, and we have followed the reviewer's suggestion. The sentence now reads:

However, more advanced aspects of primate vision, including the perception of complex shapes, are studied more comprehensively in macaques.

2. Introduction, last paragraph: The statement that shape selectivity is “a property likely computed within cortical area V4” may be too strong. While longer processing times are clearly required, the data do not rule out contributions from feedback or recurrent processing involving other areas. A more cautious phrasing may be appropriate.

We agree with the need for more cautious framing, and we have followed the reviewer’s suggestion. The sentence now reads:

Excitatory neurons were similarly selective for simple visual forms, yet a considerable proportion of them were also selective for complex visual forms (i.e. contour shape)—a property likely computed in V4 and downstream cortical areas.

3. I may have missed this, but what was the rationale for using both square-wave and sine-wave laser stimulation?

We thank the reviewer for inviting us to provide more clarification for this aspect of our experimental approach. We have now added relevant information in the Methods and Results sections as follows:

<u>In Methods</u>

Two patterns of optical stimulation were used: sinusoidal (2–500 Hz) and square wave (16 or 32 Hz). We used sinusoidal patterns in most trials for two key reasons. First, sinusoidal patterns deliver lower overall light power and have less abrupt light transitions compared to square wave patterns, due to the gentler rise and fall phases—properties that may confer network stability during optotagging. Second, sinusoidal patterns allow us to characterize how excitatory and inhibitory neurons respond to temporally varying patterns of optical stimulation (i.e. by computing F1 responses). Such functional comparisons across cell types are helpful for identifying optimal optical stimulation parameters for future cell type-specific perturbation experiments in macaques.

*In our experience, sinusoidal patterns work well for optotagging cortical excitatory and inhibitory neurons, as well as cerebellar Purkinje cells in macaques[1]. Nevertheless, we anticipate that other groups may wish to use standard square wave patterns for optotagging. For this reason, we also included the square wave condition in our experiments. Activation latencies obtained using sinusoidal and square wave patterns were highly correlated (**Supplementary Figure S3**), confirming that future studies can also use square wave modulation for optotagging.*

<u>In Results</u>

*The optical stimulation conditions included both sinusoidal and square wave modulation (**Figure 2A–B**; see **Methods** for rationale).*

*Sinusoidal and square wave laser conditions yielded similar activation latencies (**Supplementary Figure S3A–B**; latencies for the two conditions were highly correlated; r=0.87, p<0.0001 for E; r=0.71, p<0.0001 for I).*

4. I appreciate the demonstration of the weak correspondence between optotagged cell-type identification and waveform-based classification. This result underscores how unreliable waveform-based classification can be at the single-cell level (i.o.w., except for neurons at the tails of the distribution, it is impossible to assess whether a neuron is inhibitory or excitatory). A formal cross-validation analysis could further quantify this limitation. Regardless, this technical point deserves stronger emphasis in the discussion, and possibly even in the abstract, if space permits.

We have now conducted additional analyses on the extracellular waveforms of excitatory and inhibitory neurons, as suggested by the reviewer.

(i) Shuffle analysis based on joint waveform features

We had previously only considered statistical comparisons between the two neuronal cell types with respect to a single waveform feature (i.e. either trough–to–peak time or repolatization time). We therefore explored new statistical comparisons that considered both waveform features jointly. We first calculated the average trough–to–peak time and repolarization time for each neuronal cell type. We then calculated the observed separation between excitatory and inhibitory neurons as the pairwise distance between their average values in the two-dimensional waveform feature space. Next, to determine whether this observed separation could have occurred by chance we performed a shuffle analysis. In this analysis, we randomly shuffled the labels for excitatory and inhibitory neurons and repeatedly drew simulated populations with the same number of neurons

observed in our dataset of optotagged neurons. On each iteration, we calculated the separation between the simulated populations, generating a distribution of separations expected by chance. Finally, we calculated the probability that the observed separation for our dataset of optotagged excitatory and inhibitory neurons could have occurred by chance ($p<0.001$). Thus, this shuffle analysis confirms that the centroids of joint spike waveform features for the two neuronal cell types are significantly different.

(ii) Cross-validation analysis

To test whether waveform features could be used to accurately predict cortical cell type, we performed a cross-validation analysis on the optotagged data. In this analysis, we assessed the ability of a linear classifier to discriminate between excitatory and inhibitory neurons based on waveform features (using Matlab's fit discriminant analysis classifier fitcdiscr). In each iteration, we partitioned the optotagged data into training and testing sets (90% train,10% test); we then trained a linear classifier on the train set and used it to predict whether neurons in the test set were excitatory or inhibitory. Finally, we calculated the fraction of correct classifications across all iterations of the cross-validation procedure. This analysis produced a distribution of classification accuracies, with an average performance accuracy of 63%. Thus, this cross-validation analysis suggests that one can accurately predict neuronal cell type based on extracellular waveform parameters more than 50% of the time.

We now refer to these new analyses briefly in the revised manuscript. The new sentences in the Results and Methods are as follows:

<u>In Results</u>

*The centroids of excitatory and inhibitory neuron clusters were determined to be significantly different based on a shuffle analysis ($p<0.001$; see **Methods**).*

*Additionally, a linear classifier could predict neuronal cell type based on waveform features with an average performance accuracy of 63% (see **Methods**).*

<u>In Methods</u>

*To determine whether excitatory and inhibitory neurons differed with respect to their joint waveform features (i.e. trough–to–peak time and repolatization time), we performed a shuffle analysis. The goal of this analysis was to assess the likelihood that the observed separation between the centroids of excitatory and inhibitory neuron clusters could have occurred by chance (**Figure 3B**). We first calculated the average trough–to–peak time and repolarization time for each neuronal cell type, and we took the pairwise distance between their average values as the observed separation between the neuronal cell types in the two-dimensional waveform feature space. On each iteration of the shuffle procedure, we randomly shuffled the labels for excitatory and inhibitory neuron data and drew simulated populations of the same size as our datasets, and we calculated the average separation between the centroids of the simulated populations. Finally, we used the distribution of separations generated across all iterations of the shuffle analysis (N=1000) to calculate the probability that the observed separation for optotagged data could have occurred by chance. We found that the observed separation between optotagged excitatory and inhibitory neurons was statistically significant ($p<0.001$). Thus, this shuffle analysis confirms that the centroids of spike waveform features for excitatory and inhibitory neurons are significantly different.*

To test whether we could predict cortical cell type based on waveform features, we performed a cross-validation analysis based on a linear classifier approach. In this analysis, we assessed the ability of a linear classifier to discriminate between excitatory and inhibitory neurons based on waveform features (using Matlab's fit discriminant analysis classifier fitcdiscr). On each iteration of the cross-validation procedure, we partitioned the optotagged data into training and testing sets (90% train,10% test), and we trained a linear classifier on the train set and used it to predict whether neurons in the test set were excitatory or inhibitory. Finally, we calculated the fraction of correct classifications across all iterations of the cross-validation analysis (N=1000). This analysis produced a distribution of classification accuracies, with an average performance accuracy of 63%. Thus, this cross-validation analysis suggests that one can accurately predict cell type based on extracellular waveform parameters ~60% of the time.

Guided by the reviewer's feedback, we have also added a short sentence to the Abstract as follows:

Excitatory and inhibitory neurons showed moderate differences in their extracellularly recorded spike waveforms and more substantial differences in their visual response properties.

5. If I understood correctly, only one optotagged inhibitory neuron showed shape selectivity. Given that Dlx specificity is not perfect, it seems conceivable that this neuron may have been misclassified. This possibility may be worth briefly mentioning?

The reviewer is indeed correct, and we have followed their suggestion. The sentences now reads:

*In fact, only one inhibitory neuron was classified as selective for contour shape (see **Figure 4F**). Given the known, imperfect targeting selectivity of AAVs containing the mDlx5/6 enhancer, it is conceivable that this shape-selective neuron was not inhibitory.*

6. Figure 1 shows that layer 4 contains the fewest transduced neurons (for both Dlx5/6 and CaMKII), consistent with previous reports. Given this, I was surprised by the apparently homogeneous distribution of optotagged neurons as a function of cortical depth (Fig. S8). In this context, the statement on page 8 ("...consistent with our histological analyses...") does not seem fully justified. Do the authors have an explanation for this discrepancy, beyond the inherent difficulty of assigning precise laminar locations during electrophysiological recordings with single contact points?

We thank the reviewer for raising this point. We had originally intended to emphasize the overall similarity in the cortical depth profiles of excitatory and inhibitory neurons, owing to the similar laminar expression patterns of the the two viral vectors used (i.e. both vectors transduce superficial and deep layers and avoid the intermediate layer). To provide more clarity, ee have now revised this sentence as follows:

*These findings demonstrate that the laminar profiles of excitatory and inhibitory neurons sampled in the study were qualitatively similar, consistent with the comparable laminar transduction patterns of the two AAVs used (see **Figure 1C–D**).*

In agreement with the reviewer's intuition, we had hoped to see a gap corresponding to the intermediate layer in our distributions of relative cortical depth (**Figure S8**). Our explanation for the absence of such a gap is:

(i) Limited accuracy of our estimates of relative cortical depth

Two key factors are relevant here. First, our estimates of brain surface have limited accuracy. We typically lower the electrode before the optical fiber and obtain a first estimate of brain surface. We then lower the optical fiber, which often dimples the dura significantly prior to penetrating it, and thus our initial estimates of brain surface may no longer be accurate following fiber penetration. Second, our estimates of the position of each optotagged neuron relative to gray matter thickness have limited accuracy. To avoid damaging transduced neurons in the region of interest, we rarely lower the electrode to the end of gray matter in each recording session, and thus we do not acquire an estimate of gray matter thickness for each penetration.

(ii) Modest extent of the intermediate layer in V4

Based on our DAPI staining of V4 histological sections in Monkey R (**Figure 1; Supplementary Figure S1**), we estimate the thickness of the intermediate layer to be on the order of 250 µm. Thus, it would be fairly easy to miss such a small gap in the positional distributions of optotagged neurons, especially given the limited accuracy in our estimates of relative cortical depth.

7. Figure 7C and D (Page 10): The final result described here is anecdotal but highly intriguing. It is reminiscent of behavioral findings showing that optogenetic stimulation of FEF neurons primarily evokes saccades (only) when combined with low-amplitude electrical stimulation (J Neurosci, 2013; DOI:10.1523/JNEUROSCI.2675-13.2013). It is also consistent with evidence that FEF microstimulation strongly modulates visual cortical activity only in the presence of a retinal stimulus (Nature, 2003; Science, 2007). This connection might be worth briefly noting.

We thank the reviewer for pointing out this relevant literature. We have now followed the reviewer's suggestion by adding the following sentences to the Discussion section:

Our observation of many unresponsive neurons may be related to other observations made in macaques. For example, optogenetic stimulation of neurons in the frontal eye fields only evokes saccades when combined with low-amplitude electrical stimulation[2]; similarly, electrical stimulation of these neurons strongly modulates visual cortical activity only when combined with a visual stimulus[3-5]. Future studies are needed to determine the nature of the coincident inputs and the permissive conditions necessary for activating unresponsive inhibitory neurons in V4.

Note: We found a relevant paper by Moore & Armstrong (Nature, 2003), but not one published in (Science, 2007). We think the reviewer may have intended to refer to Armstrong & Moore (PNAS, 2007) or to Ekstorm et al, (Science, 2008). We now cite all three papers in the revised manuscript and we would gladly revise the citations further, as needed.

8. Page 11, second paragraph: The claim that contour shape selectivity emerges over time from orientation-selective inputs is not directly supported by the data. While this interpretation is plausible, other mechanisms, some of which are already listed, may be more important. A more cautious phrasing is recommended.

We thank the reviewer for encouraging us to use more cautious phrasing here. We agree with the reviewer's general sentiment that orientation-selective inputs alone may not be sufficient to generate shape selectivity in V4 neurons, although we note that our findings are consistent with prior work showing that orientation selectivity emerges before shape selectivity in V4 neurons (Yau, Pasupathy, Brincat & Connor, 2013). We have followed the reviewer's recommendation, and the revised sentence now reads:

Excitatory neurons may compute contour shape information gradually over time, possibly from orientation-selective inputs as suggested in prior work[6] and other dynamically changing afferent inputs.

9. Page 14: Injection depth: The Methods state that injections were made throughout the depth of gray matter (~5 mm). Given that macaque neocortex is approximately 2 mm thick (or less), does this imply that injections extended beyond the prelunate gyrus (e.g., into the STS or lunate sulcus)?

We thank the reviewer for inviting us to clarify this aspect of our experimental approach. We expand on this in detail below:

(i) Our V4 recording chambers are positioned quite lateral (centered ~25mm from midline), and our recording and injection tracks often traverse a highly curved portion of dorsal area V4. Based on brain reconstructions derived from anatomical MRI scans for each animal, we estimate that V4 gray matter thickness in our regions of interest is on the order of ~2.5–3.0 mm.

(ii) As in our previously published optogenetic work, we always begin AAV injections with the cannula positioned slightly below gray matter so as to ensure that AAV particles diffuse gradually throughout the cortical region of interest during the injection procedure. We also end AAV injections with the cannula positioned slightly above estimated brain surface in case the veridical position of brain surface changes during cannula retraction, thereby ensuring that AAV particles reach the most superficial layers of cortex.

(iii) In ~2–3 recording sessions, we encountered optotagged neurons deep in gray matter that had very large and/or peripheral receptive fields (consistent with MT/MST neurons in the STS). In these very rare cases, we elected not to test these optotagged neurons further, and we moved our electrodes to a different, more superficial location.

(iv) Our experiments rely on the use of blue light illumination, which is known to only travel ~500 μm in cortex. Thus, even if some neurons in the STS were indeed transduced, it is highly unlikely that we would have activated them by recording in dorsal V4.

We have now expanded the Methods section to incorporate relevant information as follows:

In Methods, under "AAV vector injections"

AAV vector injections were made at multiple sites throughout the depth of V4 grey matter, starting from the deepest point and spaced 500 μm apart. We injected along a ~5mm track, starting slightly below V4 gray matter and ending slightly above brain surface. Because the recording chambers were placed very laterally (centered ~25 mm from midline), the injection and recording tracks often traversed a highly curved portion of dorsal area V4, and gray matter thickness was estimated to be ~2.5–3.0 mm from based on brain reconstructions generated from anatomical MRI scans.

In Methods, under "Data inclusion criteria"

*We ensured that all optotagged neurons in our dataset had receptive fields consistent with the known topography of dorsal area V4[7] (**Supplementary Figure S10**). In less than a handful of recording sessions, we encountered optotagged neurons deep in gray matter that had much larger and/or more peripheral receptive fields, consistent with neurons located in the superior temporal sulcus. In these rare cases, we elected not to test these neurons further, so as to ensure correct identification of neurons within V4.*

10. The laser power used is relatively high. Although thermal artifacts seem unlikely, can they be completely excluded? In principle, such effects could differentially influence excitatory and inhibitory neurons.

We thank the reviewer for inviting us to expand on this aspect of our experimental approach. The laser powers used in our study are consistent with our previously published optogenetic studies in which we used blue light illumination to activate ChR2-positive inhibitory neurons in macaque V1 (De, El-Shamayleh & Horwitz. 2020) and ChR2-positive Purkinje cells in macaque cerebellum (El-Shamayleh et al, 2017). We share our rationale for using this range of powers in detail below:

(i) For functional studies, the most relevant quantity is likely "power density" (not absolute power), which takes into account the total laser power used *and* the optical fiber diameter. Our optical fiber diameter is 300 µm, often larger than that used by other labs (~200 µm) so as to ease entry through thick primate dura without the aid of a penetrating guide tube. Thus, the same value of absolute power (e.g. 100 mW) is effectively distributed over a wider sectional area of optical fiber, resulting in a lower power density. Additionally, different opsins have different wavelength and different power sensitivities, making it difficult to make head-to-head comparisons of laser power across different macaque studies and laboratories. Nevertheless, the laser parameters used in our study are closely in line with our prior macaque optogenetic studies based on ChR2/blue light, and we are using an identical laser setup and optical fiber as in this prior work.

(ii) In the sinusoidal and square wave optical stimulation conditions used in our study, the light is only on for half of the stimulation epoch, so the total power delivered is far less than in the commonly used constant-step optical stimulation condition used in other studies.

(iii) In practice, we always chose the minimal laser power needed to elicit clear, time-locked responses for each optotagged neuron, based on manual tests. To mitigate concerns about thermal or light artifacts, we routinely conducted manual tests with red illumination instead of blue, and we did this for both excitatory and inhibitory neurons. Specifically, by toggling between blue and red light (via a custom remote control connected to a laser that can interleave blue and red light in a single optical fiber), we confirmed selective neural activation by blue light.

We have now expanded the Methods section to incorporate the following relevant information:

For each optotagged neuron, we chose the minimal laser power needed to elicit clear, time-locked neural responses based on manual tests. In most sessions, we also manually confirmed selective neuronal responses to blue (450 nm) but not red (638 nm) light illumination, thereby mitigating concerns about thermal or light artifacts.

11. Were non-optotagged neurons recorded in regions where viral injections were performed (i.e., neurons that were simply not transduced), or were these recordings obtained from cortical sites without viral expression?

We previously described non-optotagged neurons as being "sampled within the same cortical sites where we recorded optotagged neurons", meaning that they were recorded within the same cortical sites where the viral vector injections were performed, but in separate recording sessions. We made this experimental choice to ensure that our non-optotagged neurons represented the "generalist" population of V4 neurons nearby our optotagged neurons. To improve the clarity of our text, we have expanded this description in the Methods section as follows:

Non-optotagged neurons were sampled within the same cortical sites where we injected AAV vectors and where we recorded optotagged neurons. Non-optotagged neurons were recorded at similar microdrive x/y coordinates where optotagged neurons were recorded, but in separate experimental sessions (e.g. during routine experiments for basic receptive field mapping and other data collection sessions conducted before and after AAV vector injections were made). This approach ensured that data from non-optotagged neurons could serve a suitable benchmark for functional comparisons between optotagged neurons and the generalist population of V4 nearby.

12. I may have missed this, but what criterion was used to classify a neuron as visually responsive?

The reviewer is correct in that we had mentioned this point only briefly in the Methods section. The classification of neurons into visually responsive and unresponsive groups was largely qualitative and based on the initial receptive field mapping tests conduced for each optotagged neuron. In a few sessions, we tried to record data files documenting the absence of spiking activity in non-responsive neurons, but we ultimately moved away from this strategy after clearly noting the lack of responses in these neurons, even after several

minutes of stimulus presentation. To improve clarity, we have expanded this description in the Methods section as follows:

*We started each data collection session by attempting to drive each optotagged neuron, under passive viewing conditions, using a standard battery of visual stimuli commonly used in V4 studies. The stimuli included drifting sinusoidal gratings, spots of light, oriented bars and two-dimensional geometric shapes; the stimuli could also vary in position, size, color and rotation, under the experimenter's control. Based on this initial and qualitative receptive field mapping procedure, we classified each optotagged neuron as visually responsive or unresponsive. Many optotagged neurons were visually responsive, but some were determined to be unresponsive (**Figure 5A**). In the case of unresponsive neurons, we always confirmed that we could readily activate nearby neurons using the same battery of stimuli, thereby confirming the suitability of our stimuli for driving nearby V4 neurons.*

Reviewer #2

In their manuscript titled 'Optotagging in primate cortex uncovers excitatory and inhibitory neuron specializations in form vision', Chuyi Su and Yasmine El-Shamayleh demonstrate an optogenetic approach in macaque V4 to directly dissociate excitatory and inhibitory neuron contributions to visual form processing. The authors demonstrate the technical feasibility of cell-type specific viral targeting and in vivo optical identification in primate cortex, which represents an important methodological step for circuit-level investigations in non-human primates. Functionally, the data show that excitatory neurons are more visually responsive and exhibit stronger contour shape selectivity than inhibitory neurons, which appear to primarily encode simple orientation information. The temporal analyses further suggest delayed emergence of contour shape selectivity relative to orientation tuning and differences in response dynamics across cell types. These findings clarify how form information may be distributed across cortical cell types and highlight the promise of optogenetic tagging for computational modeling and causal circuit analysis in primates. While the study is technically ambitious and addresses an important question, several aspects of the analyses and interpretations require clarification and more cautious framing. I outline these major concerns below.

We thank Reviewer #2 for the constructive feedback, including several excellent suggestions for targeted data analyses and more cautious framing of our results that have greatly strengthened the revised manuscript. We are also pleased that the reviewer values our demonstration of optotagging as "an important methodological step for circuit-level investigations in non-human primates" and considers the study "technically ambitious and addresses an important question".

Major comments

1) The histological validation (figure 1C–D) was performed in a different animal (Monkey R), whereas neurophysiology was conducted in Monkeys C, B, and D. While the images suggest cell-type specificity, the localization of ChR2 in the soma of V4 excitatory neurons appears relatively sparse compared to inhibitory expression. There is no quantification of ChR2-eYFP expression relative to CaMKII-positive cells, making it unclear what fraction of excitatory neurons were successfully transduced. Although sparse expression may not compromise light-evoked control of optotagged neurons' spiking for identification purposes, it raises concerns about sampling bias. If only a subset of pyramidal neurons expresses ChR2, the optotagged excitatory population may not represent the broader excitatory pool in V4. This is particularly relevant given the central claim that excitatory neurons preferentially encode complex contour shape information. Supplementary Table S2 also suggests relatively low optotagged yield compared to non-tagged neurons across animals. Quantification of transduction efficiency (e.g., ChR2+/CaMKII+ ratios) and clarification of expression consistency across monkeys would substantially strengthen the claims of cell-type specificity and generalizability of the findings (e.g., how effectively this approach could manipulate excitatory populations to probe V4-mediated perception at the circuit level).

We are indebted to the reviewer for raising this point, and we apologize for creating this confusion regarding the robustness of our excitatory neuron targeting based on the histological figure we provided. To clarify, the expression levels of AAV–CaMKII–ChR2–eYFP in Monkey R were in fact quite robust, consistent with several previous studies using the CaMKII promoter in macaques. Indeed, most of the histological sections we processed immunohistochemically showed very strong ChR2–eYFP expression, extending throughout apical and basal dendrites and long range projection axons, all of which are diagnostic morphological features of excitatory neurons but not inhibitory neurons (see additional data provided below). This strong expression in excitatory neuron processes actually made it challenging for us to clearly visualize cell bodies across cortical layers. To overcome this challenge, for our main histological figure, we selected a histological section that was ~3 mm away from the AAV vector injection site, and we used lower antibody concentrations so as to better visualize a collection excitatory cell bodies across cortical layers—choices that resulted in what appeared to the reviewer as weak ChR2 expression in excitatory neurons. We have now added a supplementary figure showing opsin expression closer to the CaMKII injection site (**Supplementary Figure S1**; see further below).

We also thank the reviewer for suggesting CaMKII antibody staining to assess the strength of ChR2 expression in our excitatory neurons. We admit that this particular antibody staining has proven to be quite challenging for us in practice. The corresponding author has previously attempted to use mouse CaMKII antibodies in macaque cortical sections repeatedly, but without success. She has also confirmed via personal communications with an expert colleague (Dr. Gregory Horwitz at the University of Washington) that his group also has not had any success with CaMKII antibodies in macaque cortex.

We also thank the reviewer for inviting us to provide further clarification regarding the yield of optotagged versus non-optotagged neurons. We apologize that our **Supplementary Table S2** has caused some unnecessary confusion. Optotagged and non-optotagged neurons were recorded in separate experimental sessions but at similar microdrive locations. The primary goal of our study was to sample and compare the response properties of a similar number of excitatory and inhibitory neurons (59 E; 60 I). As additional experimental controls, we also sampled a similar number of non-optotagged neurons (83 in CaMKII sites; 82 in mDlx sites). Thus, differences in the total number of optotagged and non-optotagged neurons listed in the table does not reflect a lower yield of excitatory neurons or a difficulty in optotagging these neuron. Rather the table merely presents the size of our different datasets, which we generally tried to match across excitatory and inhibitory neurons, and across non-optotagged neurons at CaMKII and mDlx sites.

We have attempted to address the reviewer's core concerns in three ways:

(i) Additional histological data

We now provide a new histological figure demonstrating more robust ChR2 expression in excitatory neurons near the injection site (**Supplementary Figure S1**). This section shows strong expression in apical and basal dendrites, and in long range projection axons in white matter below V4 cortex—all of which are characteristic of excitatory neurons and not inhibitory neurons. There are several eYFP-positive cell bodies throughout the image, but these are largely obscured by the strong expression in neuronal processes. These data demonstrate the robustness of the AAV vector used for targeting excitatory neurons.

(ii) Improved description of histological methods

We have now expanded the description of our histological methods for AAV–CaMKII–ChR2–eYFP to avoid any confusion. We have also added a sentence in the figure legend for maximal clarity. The new text reads:

<u>In Methods</u>

*In the case of both viral vectors used, we observed robust AAV-mediated ChR2 expression in many neurons, spanning ~2–4 mm of V4 cortex. At sites injected with AAV–CaMKII–ChR2–eYFP, we observed strong ChR2 expression that extended throughout apical and basal dendrites and long range projection axons, all of which are diagnostic morphological features of excitatory neurons but not inhibitory neurons (**Supplementary Figure S1**). This strong expression in excitatory neuron processes hindered our ability to clearly visualize excitatory cell bodies across cortical layers. Thus, for the main histological figure (**Figure 1C**), we selected a histological section that was 3 mm away from the AAV vector injection site, and we used lower antibody concentrations so as to optimally reveal a few excitatory cell bodies across cortical layers.*

<u>In Legend of Figure 1</u>

*This histological section was taken several millimeters away from the injection site and processed with lower antibody concentration to reveal a few excitatory neuron cell bodies, without obstruction by the robust expression in apical and basal dendrites and long-range projection axons (see **Supplementary Figure S1**; also see Methods for details).*

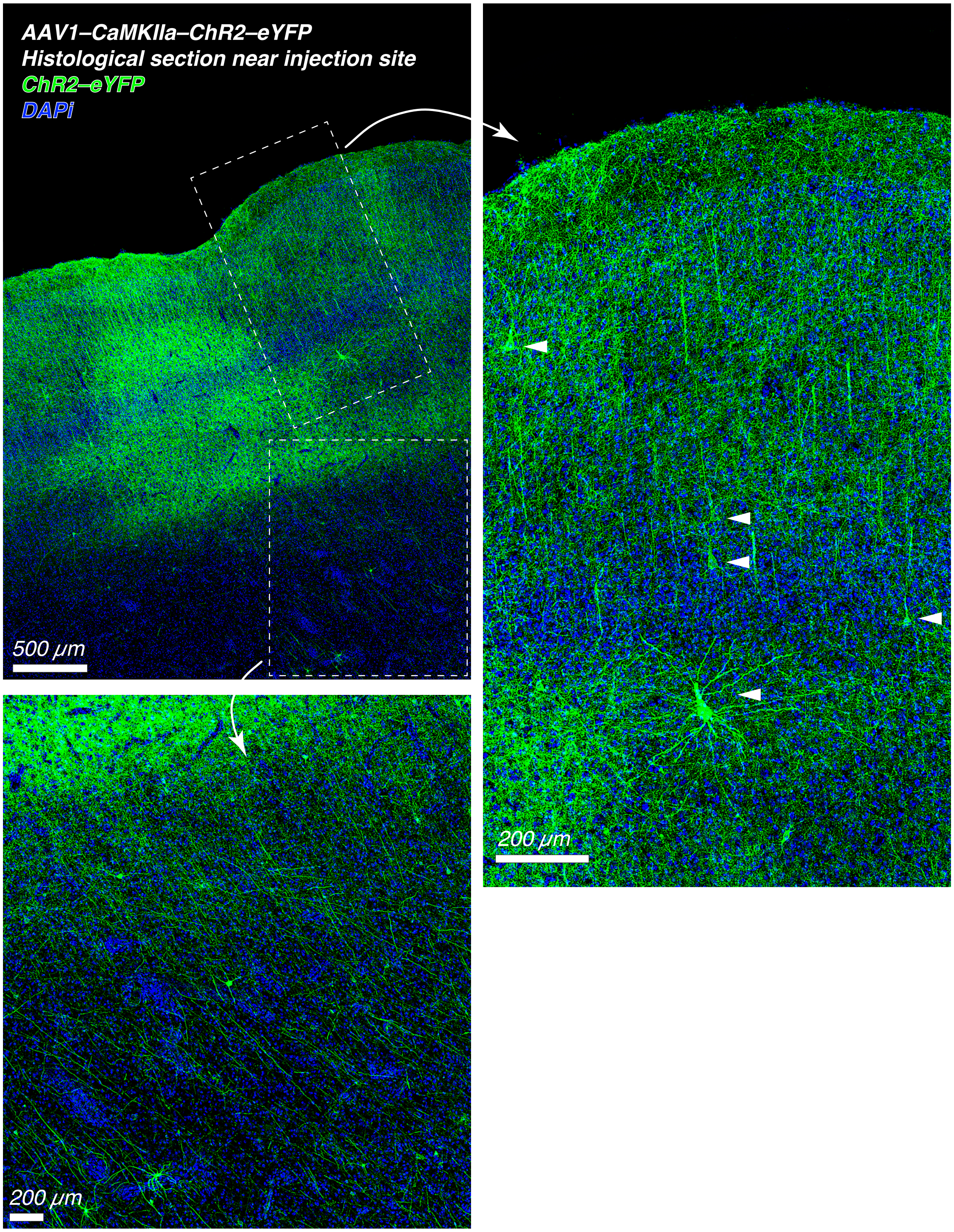

Supplementary Figure S1. ChR2 expression close to the CaMKIIa vector injection site. Top left: Histological analysis of a V4 cortical site in Monkey R injected with AAV1–CaMKIIa–ChR2–eYFP (20X montage; scale bar corresponds to 500 μm). ChR2-positive neurons and processes are in green; nuclei stained by DAPi are in blue. The image shows strong expression across most cortical layers (except layer 4), including in apical and basal dendrites and in axons—all of which are characteristic features of excitatory neurons but not inhibitory neurons. A few pyramidal cell bodies are visible across layers but most are occluded by the strong expression in neuronal processes. Top right: expanded image of the region outlined in the dashed white box within gray matter, showing strong eYFP expression in several pyramidal cells marked by triangles (20X montage; scale bar corresponds to 200 μm). Bottom left: expanded image of the region outlined in a dashed white box within white matter, with increased gain of the eYFP signal to show expression in projection axons in white matter clearly (20X montage; scale bar corresponds to 200 μm).

(iii) Improved description of optotagged vs. non-optotagged neurons

We have now expanded the description of how optotagged and non-optotagged neurons were sampled in our study. We have also added several sentences in the supplementary table legend for maximal clarity. The new text now reads:

In Methods, under "Data inclusion criteria"

*The study is based on data collected from 119 optotagged neurons: 59 excitatory neurons and 60 inhibitory neurons (**Supplementary Table S2**; 59 neurons from Monkey C; 19 from Monkey B and 41 from Monkey D). We also include data collected from 165 neurons recorded without knowledge of their cell type identity, referred to here as non-optotagged (**Supplementary Table S2**; 83 neurons from Monkey C; 20 neurons from Monkey B and 62 neurons from Monkey D). Non-optotagged neurons were sampled within the same cortical sites where we injected AAV vectors and where we recorded optotagged neurons. Non-optotagged neurons were recorded at similar microdrive x/y coordinates where optotagged neurons were recorded, but in separate experimental sessions (e.g. during routine experiments for basic receptive field mapping and other data collection sessions conducted before and after AAV vector injections were made). This approach ensured that data from non-optotagged neurons could serve a suitable benchmark for functional comparisons between optotagged neurons and the generalist population of V4 nearby.*

In the legend of Supplementary Table S2

We recorded optotagged and non-optotagged neurons within the same cortical sites in three macaques, but in separate experimental sessions. We sampled a similar number of excitatory and inhibitory neurons (59 E and 60 I), and a similar number of optotagged neurons across injection sites (83 in AAV–CaMKII sites and 82 in AAV–mDlx sites). Optotagged and non-optotagged neurons were recorded across many electrode penetrations and over a large area of cortical area V4; the listed number of penetrations and cortical area sampled applied to both optotagged and non-optotagged neurons.

2) This laminar bias of ChR2 expression, i.e., little to no expression in the granular layer, might significantly limit interpreting the results. In primate V4, layer 4 receives strong feedforward input from earlier visual areas (e.g., V2) and plays a key role in shaping orientation and contour selectivity before information is distributed to superficial and deep layers. If excitatory and inhibitory neurons in layer 4 are underrepresented or unlabeled, the optotagged population may not fully capture the feedforward circuitry contributing to visual form processing. This limitation could constrain the ability to functionally dissociate cell-type contributions across cortical microcircuits, particularly when interpreting differences in shape selectivity and response dynamics. For example, conclusions about inhibitory neurons signaling primarily simple features may reflect sampling bias away from specific laminar subpopulations rather than a generalized property of V4 inhibitory neurons. Clarifying laminar expression patterns and recording depth distributions relative to layer boundaries would strengthen the cell-type specific interpretation of the findings.

We are grateful to the reviewer for raising this important point. We fully agree that the weak transduction of cortical neurons in the granular layer, observed in both the current study and our prior optogenetic work (e.g. El-Shamayleh & Horwitz, 2019; De, El-Shamayleh & Horwitz, 2020), may limit the insights drawn from optotagging studies. Guided by the reviewer's feedback, we now openly acknowledge this current limitation of AAV-mediated transduction in macaques in an expanded Discussion section, including how it may impact the interpretation of optogenetic circuit dissection studies. This text now appears under a separate sub-header:

In Discussion, under "Limitations of the study"

*The optotagging strategy we developed and applied in the current study is not without limitations. While our use of single recording electrodes enabled clear identification of optotagged neurons with high isolation quality, this approach hindered us from surveying neuronal functional properties at a larger spatial scale. We were unable to compare the properties of excitatory and inhibitory neurons across different cortical layers. We were also unable to probe functional interactions between optotagged neurons and other surrounding neurons, with the exception of one experimental session where we were fortunate to record two well-isolated neurons simultaneously (see **Supplementary Figure S14**). Future studies combining cell type-specific optogenetics with large-scale neural recordings across cortical layers are therefore needed to yield such mechanistic insights. Additionally, the weak AAV-mediated transduction of neurons in the granular layer, as observed in this and other studies[8-10], limits the interpretation of optogenetic circuit dissection studies in the macaque cerebral cortex. Specifically, the inability to transduce and identify optotagged neurons in the granular layer constrains our efforts to discern cell type-specific contributions across cortical microcircuits. Future efforts to develop*

AAVs containing layer-specific gene regulatory elements will greatly facilitate the investigation of laminar cortical computations in macaques.

We also agree with the reviewer about the immense value of layer-specific analyses of the functional properties of excitatory and inhibitory neurons. However, we are concerned that our experimental approach is not ideally suited to provide definitive results. Specifically, two aspects of our approach make it difficult to assign our optotagged neurons relative to layer boundaries:

(i) Single electrode recordings

We used high-impedance single tungsten electrodes for all of our optotagging recordings. While this approach had the key advantage of providing clear identification of optotagged neurons with high isolation quality, it hindered us from estimating the position of optotagged neurons relative to layer boundaries, either based on cortical depth, or based on functional signatures (e.g. current sources and sinks).

(ii) Limited accuracy of our estimates of relative cortical depth

Two key factors are relevant here. First, our estimates of brain surface have limited accuracy. We typically lower the electrode before the optical fiber and obtain a first estimate of brain surface. We then lower the optical fiber, which often dimples the dura significantly prior to penetrating it, and thus our initial estimates of brain surface may no longer be accurate following fiber penetration. Second, our estimates of the position of each optotagged neuron relative to gray matter thickness have limited accuracy. To avoid damaging transduced neurons in the region of interest, we rarely lower the electrode to the end of gray matter in each recording session, and thus we do not acquire an estimate of gray matter thickness for each penetration.

These aspects of our experimental approach greatly limited our ability to reliably quantify the recording depth distributions of optotagged neurons or to assign optotagged neurons to different laminar compartments. Admittedly, our prior attempt of estimating the relative cortical depth of each optotagged neuron based on its distance from brain surface (as shown in **Supplementary Figure S9B**) represented our best effort to get at the reviewer's core point given the constraints of our datasets. And fortunately, this analysis demonstrated the lack of clear differences in relative cortical depth between our optotagged excitatory and inhibitory neurons, suggesting that cortical depth alone cannot explain away the functional differences we observed between the two cortical cell types. For both neuronal cell types, we recorded a roughly equal proportion of neurons in the top and bottom halves of cortex (for E, 47% in top and 53% in bottom; for I, 49% in top and 50% in bottom). And for both neuronal cell types, we did not find significant correlations between orientation selectivity or shape selectivity across relative cortical depth. Of course, more incisive laminar recording experiments may be better positioned to reveal functional differences that were glossed over by our crude estimates of cortical depth.

Given these various factors, we feel it would be best to clearly and openly acknowledge the limitations of our approach (as we have done in the revised text provided earlier). Additionally, we highlight the critical need for future studies investigating the properties of excitatory and inhibitory neurons across cortical layers. Guided by the reviewer's feedback, we have now expanded the Discussion section on future directions as follows:

<u>In Discussion, under "Future directions"</u>

Our study motivates several lines of future investigation, both within and beyond visual cortical area V4. First, it is valuable to compare the responses of V4 excitatory and inhibitory neurons across a broader array of visual stimuli, and across different behavioral states; such studies may clarify the contribution of visual and other inputs to the responses of each neuronal cell type. Second, it is valuable to compare the properties of V4 excitatory and inhibitory neurons across different cortical layers; such studies may clarify how the two neuronal cell types uniquely contribute to visual processing in specific microcircuits. Third, it is valuable to probe functional interactions between V4 excitatory and inhibitory neurons, and between optotagged neurons and surrounding neurons; such studies may clarify principles of cortical processing at the population-level. Fourth, it is valuable to compare the response properties of inhibitory neurons across many visual cortical areas; such studies are necessary for evaluating the specificity and generality of inhibitory neuron function across stages of information processing. Fifth, it is valuable to expand the optotagging approach to study the functional properties of molecularly distinct inhibitory neurons; such studies will advance a more comprehensive understanding of how inhibitory circuits influence cortical function in macaques.

3) While the histological images suggest broad excitatory and inhibitory targeting, the manuscript does not provide quantitative immunohistochemical characterization of which inhibitory subtypes were transduced. The mDlx5/6 enhancer labels GABAergic neurons broadly and does not distinguish among major interneuron classes (e.g., PV+, SST+, VIP+), which are known to differ substantially in connectivity and functional roles.

Without subtype-specific validation, it remains unclear how representative the optotagged inhibitory population is with respect to known molecular classes. This limitation becomes particularly relevant where functional differences between fast-spiking and regular-spiking neurons are discussed (Figure 7). Because waveform-based classification does not map cleanly onto molecular identity in primate cortex, interpretations regarding inhibitory specialization should be framed cautiously.

We thank the reviewer for encouraging us to frame these specific findings more conservatively. We fully agree with the reviewer, and we have now followed their suggestion. The revised sentences now read:

<u>In Results</u>

Following previous studies, we classified optotagged inhibitory neurons into two functional classes, fast-spiking and regular-spiking, based on the width of their extracellular waveforms[11-13]. Waveform-based classification of subtypes of inhibitory neurons is an imperfect approach that may not map precisely onto molecularly distinct cell types. Nevertheless, we leverage this approach here as a useful starting point for surveying potential functional differences between inhibitory neuron subtypes in macaques.

<u>In Discussion</u>

*Using a waveform-based classification approach, we also found that a larger proportion of unresponsive inhibitory neurons were fast-spiking compared to regular-spiking, pointing to potential functional differences between inhibitory neuron subtypes (**Figure 7B**). Given the limited accuracy of this classification approach, future studies are needed to confirm such functional differences using AAVs targeting molecularly distinct subtypes of inhibitory neurons. Nevertheless, our current findings, though limited in extent, suggest that unresponsive inhibitory neurons in V4 may require a coincidence of inputs to become visually responsive (**Figure 7C**).*

4) The central claim that excitatory neurons preferentially encode complex contour shape information. While the categorical difference in shape-selective neurons (37% E vs 6% I) is striking, the median contour shape selectivity indices differ more modestly (0.64 vs 0.50), suggesting that the sharp dissociation depends in part on the stringent ≥0.7 classification threshold. This criterion is adopted from previous V4 studies (Pasupathy & Connor, 2001; El- Shamayleh & Pasupathy, 2016), which were based primarily on extracellular recordings from putative pyramidal neurons. While appropriate for excitatory-dominated datasets, its applicability to inhibitory neurons, whose tuning properties, firing rates, and response variance may differ requires explicit justification. Applying the same threshold across cell types assumes comparable tuning structure, which may not hold for interneurons. It would strengthen the manuscript to demonstrate that the reported cell-type differences remain robust across a range of thresholds and to frame conclusions in terms of distributional shifts rather than categorical exclusivity.

We are grateful to the reviewer for suggesting this targeted supplementary analysis to strengthen our conclusions. We have now followed the reviewer's suggestion, and we describe our findings below:

To ensure that our findings did not depend on the exact criterion threshold chosen for classifying neurons as shape-selective, we varied the criterion threshold. Specifically, we repeated our analyses using a range of criterion thresholds of the shape selectivity index, from 0.55 to 0.95 in steps of 0.1. We did not use criterion thresholds lower than 0.55 because we know based on our previous V4 studies that neuronal data with shape selectivity indices at/below 0.5 are clearly not well-fit by the shape selectivity model, indicating a lack of sensitivity for contour shape information. At each of these various criterion thresholds tested, we evaluated the percentage of shape-selective neurons that were excitatory or inhibitory. We found that regardless of criterion threshold, most of the neurons classified as shape-selective were excitatory, whereas inhibitory neurons were only a minority of shape-selective neurons. We now include this analysis in **Supplementary Figure S8B**, and we refer to it in the revised Results as follows:

*To ensure that our finding of few inhibitory neurons that were shape-selective did not depend on the criterion level used to classify neurons, we tested a range of criterion values of the shape selectivity index (from 0.55 to 0.95 in steps of 0.1; **Supplementary Figure S8B**). At each criterion level tested, we re-evaluated the percentage of shape-selective neurons that were excitatory or inhibitory. Across criterion levels, we found that the large majority of neurons classified as shape-selective were excitatory. These findings demonstrate that strong selectivity for contour shape information was primarily restricted to V4 excitatory neurons.*

5) The time-to-selectivity analysis relies on fixed classification thresholds (≥0.2 for orientation; ≥0.7 for shape) to define the moment at which tuning emerges. While these thresholds are justified for classifying selectivity in

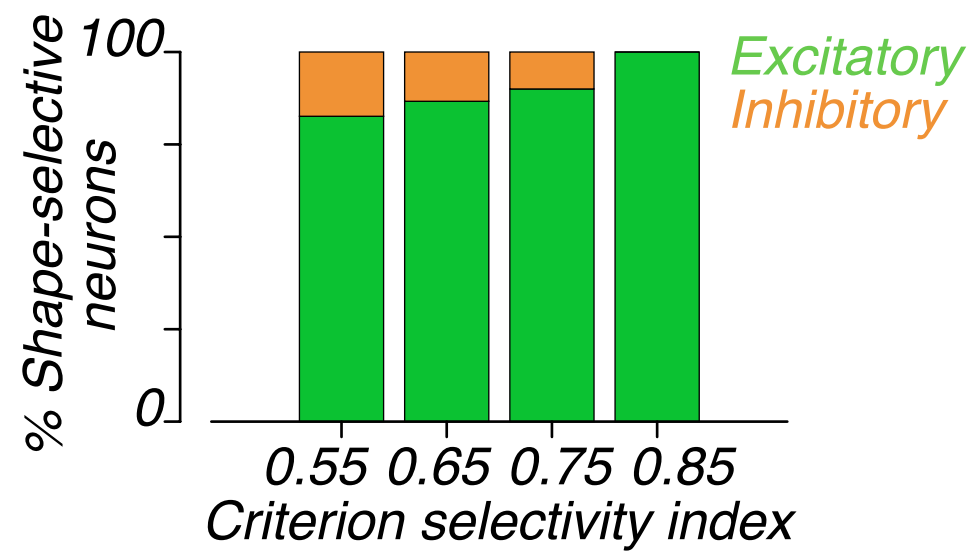

Supplementary Figure S8B. Percentage of neurons classified as shape-selective based on different criterion levels of the shape selectivity index. At all criterion levels, most of the neurons classified as shape-selective were excitatory.

prior V4 studies, using them to estimate latency may introduce bias, particularly when comparing two indices with different dynamic ranges and distributions. Because shape selectivity is defined by a more stringent criterion and a higher- dimensional model, part of the observed delay in contour shape selectivity could reflect threshold crossing dynamics rather than intrinsic computational timing. It would strengthen the conclusions to show the full temporal evolution of both selectivity indices and/or to estimate latency using a more unbiased approach (e.g., normalized growth or statistical deviation from baseline), thereby isolating genuine temporal differences in tuning emergence.

We are again grateful to the reviewer for suggesting this targeted supplementary analysis to strengthen our conclusions. We have now followed the reviewer's suggestion, and we describe our findings below:

We analyzed the responses of each optotagged neurons over progressively longer response epochs, extending through the entire stimulus presentation period (starting from each neuron's visual response latency and extending to 300 ms after that latency, in 10 ms increments). We then fit the responses in each epoch as described earlier, and we quantified neuronal selectivity for bar orientation and contour shape at each time point. As suggested by the reviewer, we now show the full time course of neuronal selectivity for each cell type (**Supplementary Figure S13**). For maximum clarity and interpretability, we only included data from neurons that were selective for bar orientation, contour shape or both.

The time course profiles reveal a clear difference in neuronal selectivity for bar orientation and contour shape, in agreement with our main findings. Neuronal selectivity for bar orientation was highest in the earliest response epochs, consistent with orientation selectivity being largely inherited through feedforward inputs from earlier cortical stages. Note that neuronal selectivity for some cells was exceptionally high in the earliest response epochs *prior* to the visual response latency of most V4 neurons (~70 ms; see vertical arrowhead along abscissa) due to very sparse responses that created spuriously high orientation selectivity indices. Neuronal selectivity for bar orientation fell gradually in later response epochs, presumably due to the influence of additional recurrent inputs, but it typically remained above the criterion threshold for selectivity (horizontal arrowhead and dashed line; ≥0.2). In contrast, neuronal selectivity for contour shape was highest in the later response epochs, reaching and maintaining criterion threshold for selectivity (horizontal arrowhead and dashed line; ≥0.7); this finding is consistent with shape selectivity being gradually computed within V4, possibly due to the influence of additional recurrent inputs. Excitatory and inhibitory neurons had similar time course profiles overall; the time course of neuronal selectivity depended more on stimulus type (simple vs complex form) than on cell type (excitatory vs inhibitory). These expanded analyses strengthen the main conclusions of our study, and they serve to demonstrate the rationale for calculating our time–to–selectivity measures in the way that we did: as the first time point at which selectivity exceeded criterion levels enabling reliable classification over two consecutive response epochs.

We now include these data in a new supplementary figure (**Supplementary Figure S13**). We also refer to these expanded results briefly in the revised manuscript as follows:

<u>In Results</u>

*By visualizing the full time course of visual form selectivity for individual neurons we confirmed that selectivity for bar orientation was typically highest in early response epochs whereas selectivity for contour shape was highest in later response epochs (**Supplementary Figure S13**).*

<u>In Methods</u>

*Data showing the full time course of each neuron's selectivity for bar orientation and contour shape are provided (**Supplementary Figure 13**).*

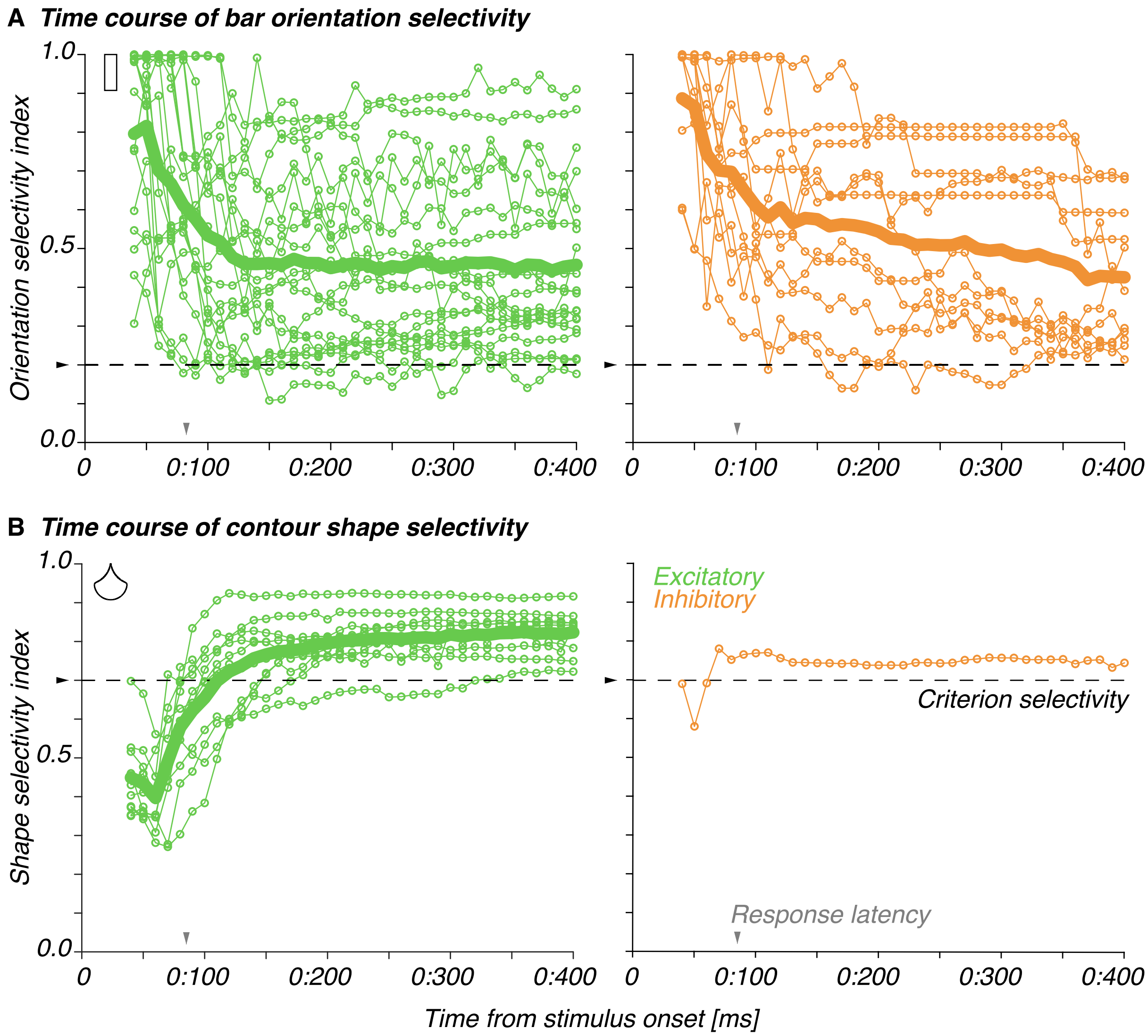

Supplementary Figure S13. Time course of visual form selectivity. We quantified neuronal selectivity for bar orientation and contour shape over progressively longer epochs, extending through the stimulus presentation period (shown along abscissa). **(A)** Time course of bar orientation selectivity for excitatory and inhibitory neurons (green and orange; thin lines are data for individual neurons; thick lines are the average; horizontal arrowhead and dashed line mark the criterion threshold for classification). Selectivity for bar orientation was typically maximal in the earliest response epochs. **(B)** Time course of contour shape selectivity for excitatory and inhibitory neurons (same format as in A). Selectivity for contour shape was typically maximal in later response epochs. Only neurons that reached criterion levels of selectivity for either bar orientation or contour shape were included from this analysis.

6) The authors conclude that visually unresponsive inhibitory neurons require coincident inputs to become active, based on supralinear responses during combined optical and visual stimulation. However, this conclusion appears based on a small number of example neurons and lacks clear population-level quantification. It remains unclear how robust or prevalent this effect is across the inhibitory population. Additionally, subtype classification based solely on waveform width (fast- vs regular-spiking) should be interpreted cautiously in primate cortex, where extracellular waveform does not map cleanly onto molecular identity. The coincidence-gating interpretation should therefore be framed more conservatively.

We thank the reviewer for encouraging us to frame these findings more conservatively. We fully agree with the reviewer, and we have now followed their suggestion. The revised sentences now read:

In Results

Following previous studies, we classified optotagged inhibitory neurons into two functional classes, fast-spiking and regular-spiking, based on the width of their extracellular waveforms[11-13]. Waveform-based classification of

subtypes of inhibitory neurons is an imperfect approach that may not map precisely onto molecularly distinct cell types. Nevertheless, we leverage this approach here as a useful starting point for surveying potential functional differences between inhibitory neuron subtypes in macaques.

While our data using combined optical and visual stimulation are limited in extent, these tantalizing findings suggest that the visual responses of some V4 inhibitory neurons may be gated by a coincidence of inputs.

In Discussion

*An alternative possibility is that unresponsive inhibitory neurons in V4 typically exist in a hyperpolarized state, becoming responsive to visual stimuli only under specific, permissive conditions. We tested this possibility in only a handful of sessions by gently depolarizing unresponsive inhibitory neurons optogenetically, and we asked whether they then became responsive to visual stimuli. In these limited cases, we found that application of low-power optical stimulation coincident with visual stimulation was sufficient to drive stimulus-evoked responses in unresponsive inhibitory neurons (**Figure 7D**). Using a waveform-based classification approach, we also found that a larger proportion of unresponsive inhibitory neurons were fast-spiking compared to regular-spiking, pointing to potential functional differences between inhibitory neuron subtypes (**Figure 7B**). Given the limited accuracy of this classification approach, future studies are needed to confirm such functional differences using AAVs targeting molecularly distinct subtypes of inhibitory neurons. Nevertheless, our current findings, though limited in extent, suggest that unresponsive inhibitory neurons in V4 may require a coincidence of inputs to become visually responsive (**Figure 7C**).*

Minor Comments

1) Although the authors provide supplementary analyses restricted to short-latency (<5 ms) neurons (supplementary figure S10 and S11) and report similar results, the main figures include excitatory neurons with longer activation latencies that may reflect indirect activation. Because inhibitory neurons do not face the same ambiguity, this introduces asymmetry in classification confidence across cell types. Given that the central claims rely on clean cell- type dissociation, it would strengthen the manuscript to either (i) present the <5 ms analyses in the main figures or (ii) more prominently demonstrate that all key statistical comparisons are robust under the stricter latency criterion.

We thank the reviewer for providing these recommendations. Given the paucity of optotagging data in macaques, we have elected to follow the reviewer's second suggestion. Specifically, when making comparisons between excitatory and inhibitory neurons throughout the Results section, we now include all of the statistics for two types of comparisons: **(i)** all optotagged neurons, regardless of their activation latency and **(ii)** only neurons with activation latencies < 5 ms. In this way, readers can directly assess the robustness of our results across the two latency inclusion criteria. Below, we list all of the revised sentences in the Results, which now read as follows:

*We also provide results for only the short-latency optotagged neurons separately (**Supplementary Figure S11–S12**), and we report all statistical comparisons between excitatory and inhibitory neurons for each of the two latency criteria.*

*Across the population, we found that optical stimulation elicited vigorous responses in both neuronal cell types, but the peak responses of excitatory neurons were lower than those of inhibitory neurons, consistent with the known biophysical properties of the two classes of cortical neurons[14] (**Figure 2D;** for all neurons, $p<0.0001$; median was 61 spikes/sec and 121 spikes/sec for E and I; for short-latency neurons, $p<0.01$; median was 84 spikes/sec and 142 spikes/sec for E and I).*

Spontaneous responses derived from trials without optical stimulation were equally low for the two neuronal cell types, consistent with reports of low spontaneous activity in cortical area V4[15] (data not shown; for all neurons, mean was 2 spikes/sec and 3 spikes/sec for E and I; for short-latency neurons, mean was 2 spikes/sec and 3 spikes/sec for E and I).

*The preferred frequency, corresponding to the peak response, was higher for excitatory neurons than inhibitory neurons (**Figure 2E;** for all neurons, $p<0.0001$; median was 17 Hz and 9 Hz for E and I; for short-latency neurons, $p<0.0001$; median was 22 Hz and 9 Hz for E and I). Similarly, the high-frequency cutoff, corresponding to the highest frequency eliciting half of the peak response, was also higher for excitatory neurons than inhibitory neurons (data not shown; for all neurons, $p<0.0001$; median was 99 Hz and 68 Hz for E and I; for short-latency neurons, $p<0.0001$; median was 116 Hz and 68 Hz for E and I).*

Despite considerable overlap, the average waveform of excitatory neurons was broader than that of inhibitory neurons (for all neurons; mean was 0.29 ms and 0.23 ms for E and I; for short-latency neurons, median was 0.32 ms and 0.23 ms for E and I).

*On average, excitatory neurons had broader extracellular waveforms than inhibitory neurons, with longer trough–to–peak times (**Figure 3C**, left; for all neurons, p<0.01; median was 0.27 ms and 0.21 ms for E and I; for short-latency neurons, p<0.001; median was 0.30 ms and 0.22 ms for E and I). Excitatory neurons also had longer repolarization times (**Figure 3C**, right; for all neurons, p<0.001; median was 0.10 ms and 0.07 ms for E and I; for short-latency neurons, p<0.001; median was 0.13 ms and 0.07 ms for E and I).*

*Excitatory neurons had higher peak responses to bar stimuli (**Figure 5B**; for all neurons, p<0.01; median was 26 spikes/sec and 11 spikes/sec for E and I; for short-latency neurons, p<0.05; median was 30 spikes/sec and 9 spikes/sec for E and I). Excitatory neurons also had higher peak responses to shape stimuli (**Figure 5C**; for all neurons, p<0.01; median was 31 spikes/sec and 17 spikes/sec for E and I; for short-latency neurons, p<0.01; median was 28 spikes/sec and 16 spikes/sec for E and I). In contrast, spontaneous responses derived from trials without visual stimulation were equally low for both neuronal cell types (data not shown; for all neurons, median was 1 spike/sec and 6 spikes/sec for E and I; for short-latency neurons, median was 2 spikes/sec and 5 spikes/sec for E and I).*

*We found that an equal proportion of excitatory and inhibitory neurons were selective for simple visual forms (**Figure 5D**; for all neurons, 74% and 69% for E and I; for short-latency neurons, 71% and 77% for E and I). In contrast, a substantially larger proportion of excitatory neurons were selective to complex visual forms (**Figure 5D**; for all neurons, 37% and 6% for E and I; for short-latency neurons, 47% and 8% for E and I).*

*Across the population of optotagged neurons, we found that excitatory and inhibitory neurons were equally selective for bar orientation information (**Figure 5E**; for all neurons, median selectivity index was 0.33 and 0.29 for E and I; for short-latency neurons, median selectivity index was 0.34 and 0.31 for E and I; arrowhead shows criterion for selectivity). In contrast, excitatory neurons were more selective for contour shape information (**Figure 5F**; for all neurons, p<0.05; median selectivity index was 0.64 and 0.49 for E and I; for short-latency neurons, p<0.01; median selectivity index was 0.70 and 0.49 for E and I; arrowhead shows criterion for selectivity).*

*This analysis confirmed that the shape selectivity indices for many optotagged neurons were statistically significant (**Supplementary Figure S8C**; for all neurons, p<0.05; 67% and 58% for E and I; median selectivity index was 0.64 and 0.49 for E and I; for short-latency neurons, p<0.005; 83% and 64% for E and I; median selectivity index was 0.70 and 0.49 for E and I). When we only included neurons with statistically significant indices, we found a larger difference in shape selectivity between excitatory and inhibitory neurons (**Supplementary Figure S8C**; for all neurons, p<0.0001; median was 0.74 and 0.54 for E and I; for short-latency neurons, p<0.0001; median was 0.74 and 0.54 for E and I).*

*This analysis confirmed that excitatory neurons were more selective for contour shape information than inhibitory neurons, and that this difference in shape selectivity held for both train and test data (**Supplementary Figure S8D;** for all neurons, p<0.05; for train data, median selectivity index was 0.66 and 0.52 for E and I; for test data, the median selectivity index was 0.48 and 0.16 for E and I; for short-latency neurons, p<0.01; for train data, median selectivity index was 0.72 and 0.52 for E and I; for test data, the median selectivity index was 0.58 and 0.20 for E and I).*

*This analysis also demonstrated that excitatory neurons were more shape-selective than inhibitory neurons (**Supplementary Figure S8E**; for all neurons, p<0.05; the median data-driven selectivity index was 1.77 and 1.10 for E and I; for short-latency neurons, p>0.05; the median data-driven selectivity index was 1.52 and 1.02 for E and I).*

*In contrast, inhibitory neurons had lower selectivity for contour shape compared to neighboring non-optotagged neurons (**Figure 5G**, right; p<0.05; median selectivity index was 0.49 for all I, 0.49 for short-latency I, and 0.56 for non-optotagged neurons).*

Inhibitory neurons had slightly earlier visual response latencies than excitatory neurons, but the difference was not statistically significant (data not shown; for all neurons, median visual latency was 55 ms and range was 37–79 ms for I and median visual latency was 61 ms and range was 40–136 ms for E; for short-latency neurons, median visual latency was 55 ms and range was 37–79 ms for I and median visual latency was 60 ms and range was 40–87 ms for E).

*Across all visually responsive neurons, bar orientation selectivity developed earlier than contour shape selectivity (**Figure 6C**; for all neurons, p<0.0001; median was 60 ms and 110 ms for bar orientation and*

contour shape; for short-latency neurons, $p<0.0001$; median was 60 ms and 110 ms for bar orientation and contour shape).

*We quantified the sustained response dynamics using a modulation index and examined this measure across the two neuronal cell types. We found that across the population of optotagged neurons, excitatory neurons had higher sustained response indices than inhibitory neurons (**Figure 6E**; for all neurons, $p<0.01$; median index was 0.76 and 0.37 for E and I; for short-latency neurons, $p<0.05$; median index was 0.76 and 0.46 for E and I).*

2) Figure 1D legend: "ChR2-positive neurons are in red; nuclei stained by DAPI are in blue" appears twice.

We thank the reviewer for catching this typo, which we have now corrected.

Reviewer #3

In the current study, the authors use cell type-selective optogenetic tools to identify excitatory and inhibitory neurons in primate V4. Although optotagging in vivo has been used in rodent models for quite some time, the use of this approach has lagged in non-human primate models due to lack of cell type-specific viral tools. Here the authors use a CAMKII promoter and the dlx enhancer to drive expression broadly in excitatory and inhibitory cells, and then further subdivide the inhibitory interneurons based on action potential waveform. They examine the visual selectivity of these classes for the first time. Overall, this is a nice demonstration of the technical approach, and the authors identify some intriguing differences between the two groups. There are three general areas where the manuscript could be improved:

We thank Reviewer #3 for the constructive feedback, including excellent suggestions for new analyses and improved text that have greatly strengthened the revised manuscript. We are also pleased that the reviewer values our "nice demonstration of the technical approach" of optotagging in macaques and the "intriguing differences" between excitatory and inhibitory neurons revealed by our study.

The study would benefit from digging a little more creatively into the dataset. The authors obviously have population recordings of non-tagged cells during stimulation of either excitatory or inhibitory neurons. Does activating interneurons reduce non-tagged cell firing, as expected? Does activating excitatory neurons recruit non-tagged cells as expected? According to the methods, the authors recorded broadband signals, meaning they also have field potential recordings. Do interneurons show selective spike-field coupling to high frequencies? The authors could do more with the dataset they have to test whether the commonly observed relationships between excitatory and inhibitory neurons and local populations/LFPs are the same for higher-order cortex as for primary sensory areas.

We thank the reviewer for encouraging us to dig deeper into our unique optotagging dataset. We have now followed the reviewer's suggestions to the best of our ability, despite limitations imposed by the nature of our neurophysiological recording approach. We address each of the analyses suggested by the reviewer below:

Interactions between optotagged and non-optotagged neurons

The reviewer suggested supplementary analyses to examine the functional interactions between optotagged and non-optotagged neurons. We had originally decided not to pursue such analyses because we were concerned that our experimental approach was not ideally suited to provide definitive results. We used high-impedance single tungsten electrodes for all of our optotagging recordings. We chose this experimental approach because it had the key advantage of providing clear identification of optotagged neurons with high isolation quality, which was critical for supporting the main goals of our study. However, this approach yielded datasets containing minimal multi-unit activity, thus limiting our access to the population neuronal responses required for the analyses suggested. Nevertheless, we made an effort to address the reviewer's point in two ways:

(i) Interactions between neighboring single neurons

In one experimental session (see data below), we were fortunate to record two well-isolated neurons simultaneously: an optotagged inhibitory neuron (orange) and a neighboring non-optotagged neuron (gray). The raster plots show that the optotagged inhibitory neuron was activated by blue light (activation latency was 3.0 ms) whereas the neighboring non-optotagged neuron was suppressed by blue light, consistent with the reviewer's expectation. We now include data from this example session in the revised manuscript (**Supplementary Figure S14**). We also refer to this dataset briefly in the Discussion section (see further below).

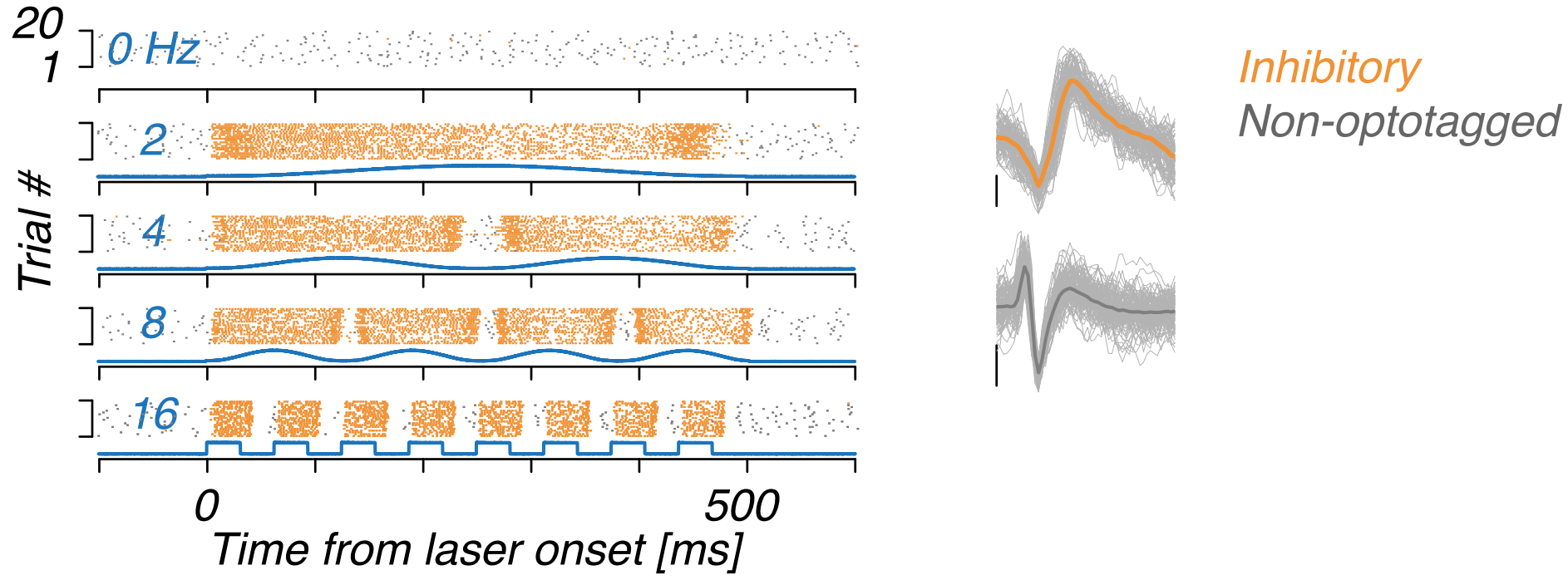

Supplementary Figure S14. Responses of an inhibitory neuron and a non-optotagged neuron recorded simultaneously. Left: Responses of an optotagged inhibitory neuron (orange) showing robust, short latency activation by blue light (activation latency was 3.0 ms). Also included are responses of a simultaneously recorded non-optotagged neuron (gray) showing suppression by blue light. Right: Extracellular waveforms of the inhibitory neuron and non-optotagged neuron. Scale bars under each waveform correspond to 10 μV.

(ii) LFP-based analyses

Guided by the reviewer's feedback, we performed bulk LFP analyses to investigate functional interactions between optotagged and non-optotagged neurons. We describe the results of this analysis below:

We processed the broadband voltage signals recorded during our optical stimulation protocol (e.g. Figure 1A–B) using Fieldtrip, a Matlab-based toolkit for LFP analysis (https://www.fieldtriptoolbox.org/). This toolkit is a standard pipeline that has been widely used in both monkey and mouse studies (e.g., Vinck et al 2015 and Vinck et al, 2013), and leverages analytical approaches to remove the influence of spikes on the LFP signal.

We focused our LFP analysis on the 8 Hz sinusoidal laser condition, which reliably drove time-locked responses from both excitatory and inhibitory neurons. For each neuronal cell type, we examined the average LFP trace triggered by the onset of optical stimulation. We found that optogenetic activation of excitatory neurons produced a clear phased-locked LFP pattern relative to the 8 Hz sinusoidal laser modulation. In contrast, optogenetic activation of inhibitory neurons produced a weaker phase-locked LFP pattern. The robust phase-locked LFP pattern seen for activating excitatory neurons may be a signature of strong excitatory-to-excitatory recurrent connectivity in cortical area V4, consistent with recent findings in mouse V1 (see O'Rawe et al, 2013).

We quantified the strength of frequency-dependent LFP modulation across excitatory and inhibitory neurons. Specifically, we compared the LFP modulation amplitude at both the 8 Hz and 2 Hz sinusoidal laser conditions, taking the ratios of the two modulation amplitudes as a measure of frequency-dependent LFP responses. We found that optogenetic activation of excitatory neurons typically resulted in greater LFP modulation at 8 Hz compared to 2 Hz, consistent with robust time-locking to the laser stimulation pattern. In contrast, activation of inhibitory neurons typically showed similar or reduced LFP modulation at 8 Hz compared to 2 Hz, consistent with weaker time-locking to the laser stimulation pattern. Collectively, these findings suggest a systematic difference in frequency-dependent coupling, with excitatory neurons showing stronger and more frequency-sensitive time-locking than inhibitory neurons.

Given the limitations imposed by our neurophysiological recording approach described above, we feel these analyses may not reach the same standard of rigor that is represented in the rest of the manuscript. We would therefore prefer to hold off on including these findings in the manuscript, and instead openly acknowledge the limitations of our approach and highlight the promise of this class of LFP measurements and analyses as a key direction for future experiments (see further below).

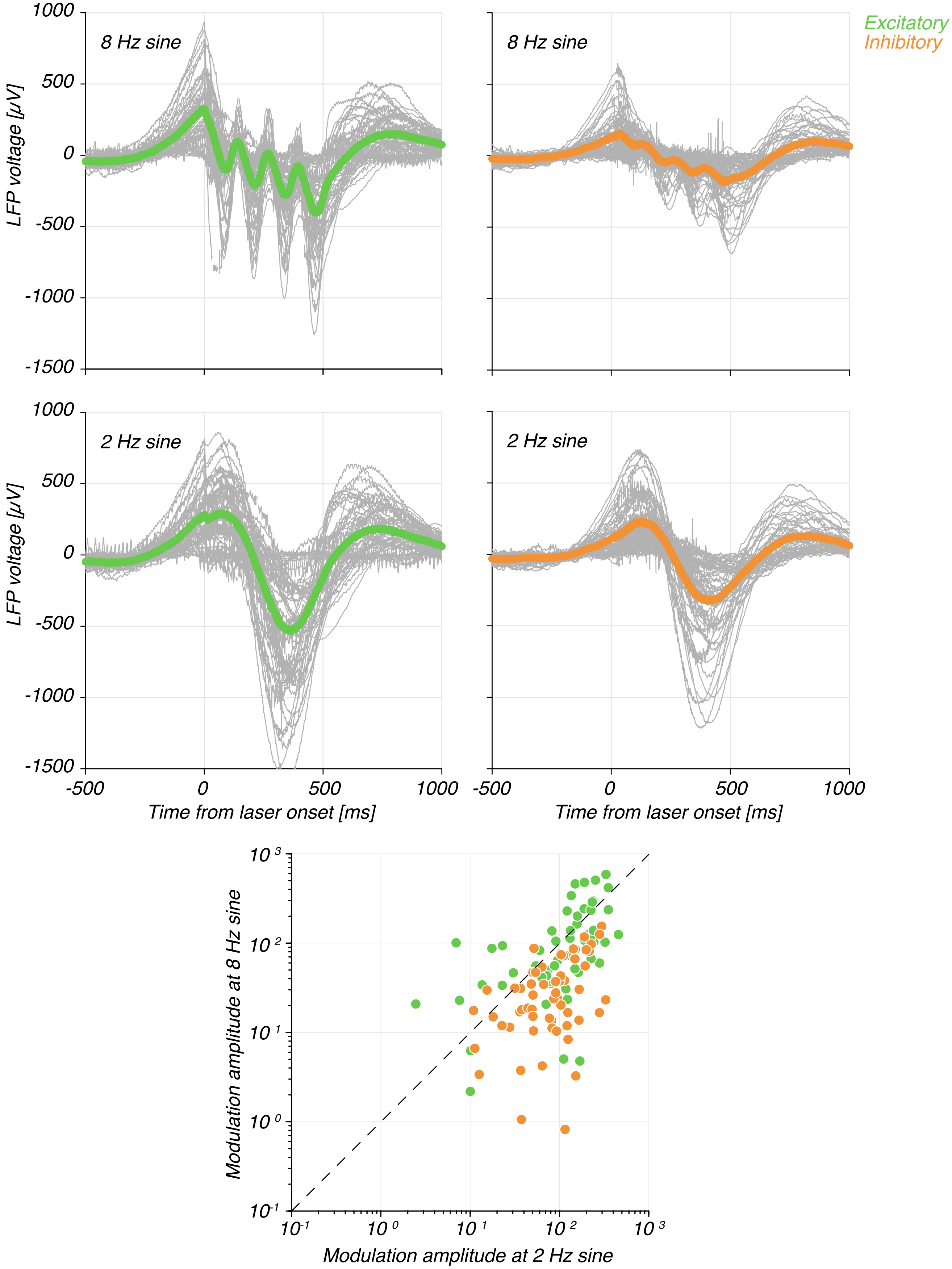

Local field potential responses to optical stimulation. See legend on following page.

Local field potential responses to optical stimulation. Top: LFP responses during activation of excitatory (green) or inhibitory (orange) neurons with an 8 Hz sinusoidal laser pattern. Data from individual sessions are in gray; average data across all sessions are in green/orange. Middle: LFP responses to a 2 Hz sinusoidal laser pattern (same format as in Top). LFP responses during activation of excitatory neurons showed stronger time-locking to 8 Hz optical stimulation compared to during activation of inhibitory neurons. Bottom: Modulation amplitude of LFP for 8 Hz and 2 Hz sinusoidal patterns. Each symbols shows data from individual sessions. The LFP modulation amplitude at 8 Hz was typically larger following activation of excitatory neurons, and weaker following activation of inhibitory neurons.

Spike-field coupling

The reviewer suggested valuable supplementary analyses using our broadband neural data, which we had recorded primarily for extracting and comparing extracellular spike waveforms quantitatively across neuronal cell types. As described earlier, we were concerned that our single electrode recording approach greatly limits the quality and spatial extent of LFP signals recorded and the subsequent interpretation of results. It is likely that neurophysiological recordings conducted with multi-contact probes or multi-channel arrays are more appropriate for supporting LFP-based analyses because signals from multiple sources can be used to reduce two major sources of experimental noise: spike artifacts and light artifacts. Nevertheless, we made an effort to address the reviewer's point in two ways:

(i) LFP-based analyses

Guided by the reviewer's feedback, we performed the spike-field coherence analysis suggested. We describe the results of this analysis below:

We computed spike-field time locking as the "pairwise phase consistency", a standard measure widely used in previous studies conducted in both monkeys and mice (e.g. Vinck et al 2015 and see, Vinck et al, 2013). We favored this measure because it is unbiased by spike rate or count. Pairwise phase consistency (PPC) quantifies how reliably spikes occur at the same phase of an oscillating LFP signal, thereby capturing the time-locking of spikes to LFP frequency. Higher PPC values indicate stronger time-locking, whereas lower PPC values indicate weaker time-locking.

We started by analyzing spikes and LFP signals recorded on a single electrode across three different optical stimulation conditions: (i) blank (0 Hz); (ii) highest laser frequency (500 Hz) and (iii) all laser frequencies (2–500 Hz);. In most of sessions, our well-isolated single neurons exhibited virtually no spiking activity in the absence of visual or optogenetic stimulation, precluding the assessment of gamma-band spike-phase coherence in the blank condition (0 Hz). Given that most spikes were confined to periods of optically driven activity, any estimate of spike-phase coupling would necessarily reflect the combined influence of gamma-band response dynamics and optically evoked responses, making it difficult to isolate intrinsic phase-locking relationships from input-driven entrainment.With this major caveat in mind, we proceeded to compute PPC across optical stimulation conditions (at highest laser frequency of 500 Hz and at all laser frequencies 2–500 Hz). We were concerned that data from the all laser frequencies (2–500 Hz) would be limited in their reliability due to potential contamination from artifacts related to optical stimulation, so we first focused our analyzing trials using the highest laser frequency (500 Hz). The 500 Hz laser stimulation condition had the benefit of driving most optotagged neurons at a reasonable level, without enforcing strong temporal modulation due to optical stimulation.

We found that in most individual sessions, PPC estimates were highly variable across LFP frequency and across recordings, and did not reveal a consistent coupling structure at the single-neuron level. We also computed the average PPC across each neuronal cell type. When considering the average trend across neurons, we again found no consistent coupling at gamma frequencies for either neuronal cell type. This was true, even in the case of analyzing all laser frequency conditions (2–500 Hz), which should in principle reveal the strongest coupling at gamma frequencies. Note that the PPC function we used in FieldTrip includes a bias correction that removes statistical dependencies between spike–LFP phases arising from within-trial spike-train history effects, such as bursting or refractoriness, and may therefore contribute to the negative values PPC values observed.

Again, given the limitations imposed by our neurophysiological recording approach described above, we feel these analyses may not reach the same standard of rigor that is represented in the rest of the manuscript. We would therefore prefer to hold off on including these findings in the manuscript, and instead openly acknowledge the limitations of our approach and highlight the promise of this class of LFP measurements and analyses as a key direction for future experiments (see further below).

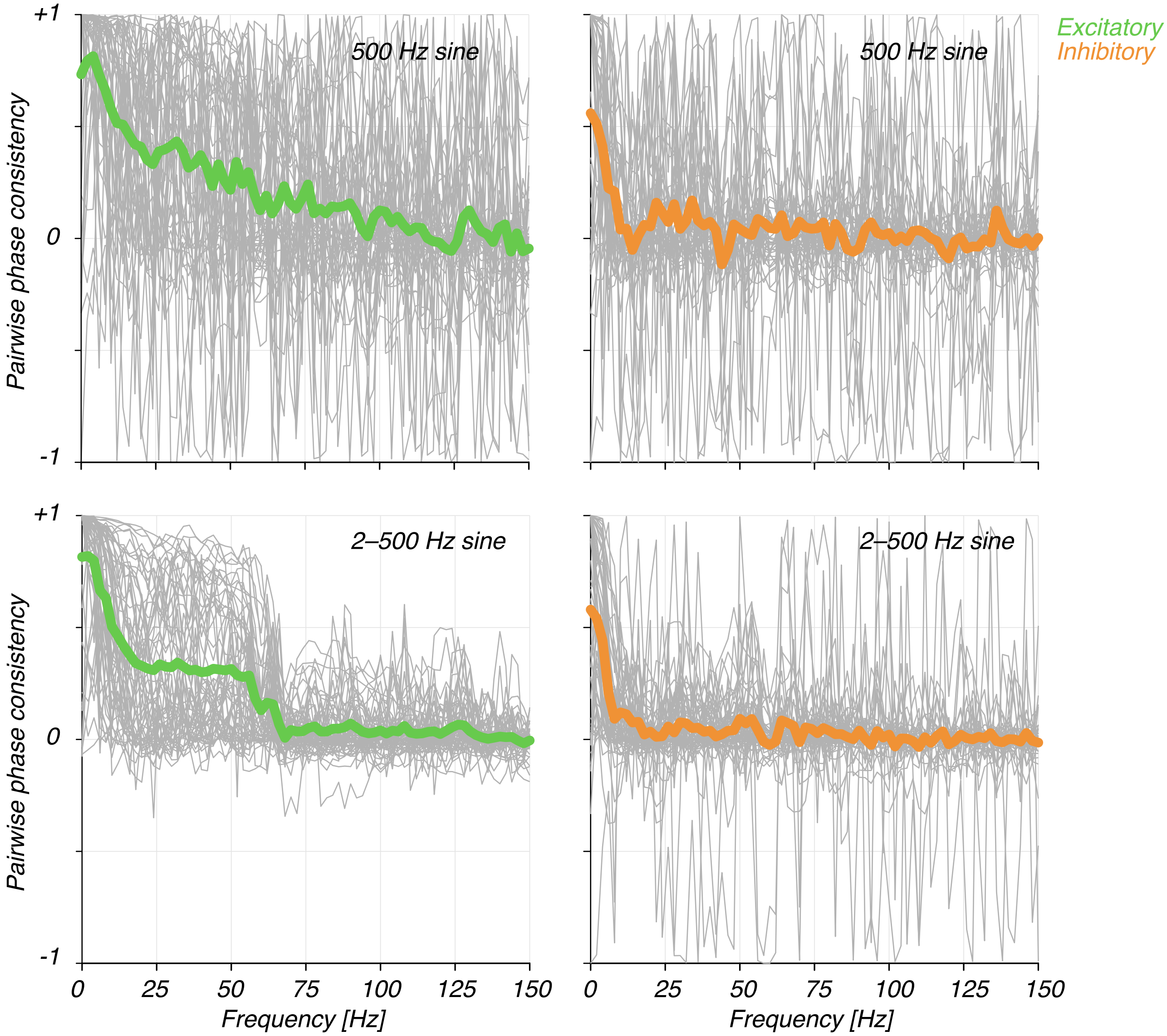

Spike–field coupling during optical stimulation. Top: Pairwise phase consistency during optogenetic activation of excitatory (green) or inhibitory (orange) neurons using a 500 Hz sinusoidal laser pattern. Data from individual sessions are in gray; average data across all sessions are in green/orange. Bottom: Pairwise phase consistency during activation of excitatory or inhibitory neurons using all frequencies tested. (2–500 Hz same format as in Top). We do not observe consistent spike–field coherence near the gamma band (~60–80 Hz). Even when using all optical stimulation frequencies (bottom), we did not find significant gamma band coupling, even though some of the laser conditions overlap gamma frequencies, and thus we expect that spiking activity should show stronger modulation.

(ii) Acknowledging limitations of our approach and highlighting the need for future studies

We now openly acknowledge the limitation of our experimental approach for addressing functional interactions between cortical cell types. We also highlight the value of future studies investigating functional interactions between optotagged and non-optotaged neurons using a combination of cell type-specific optogenetics and population recordings. Specifically we have expanded the Discussion section with new sub-headers as follows:

<u>In Discussion, under "Limitations of the study"</u>

The optotagging strategy we developed and applied in the current study is not without limitations. While our use of single recording electrodes enabled clear identification of optotagged neurons with high isolation quality, this approach hindered us from surveying neuronal functional properties at a larger spatial scale. We were unable to compare the properties of excitatory and inhibitory neurons across different cortical layers. We were also unable to probe functional interactions between optotagged neurons and other surrounding neurons, with

*the exception of one experimental session where we were fortunate to record two well-isolated neurons simultaneously (see **Supplementary Figure S14**). Future studies combining cell type-specific optogenetics with large-scale neural recordings across cortical layers are therefore needed to yield such mechanistic insights. Additionally, the weak AAV-mediated transduction of neurons in the granular layer, as observed in this and other studies[8-10], limits the interpretation of optogenetic circuit dissection studies in the macaque cerebral cortex. Specifically, the inability to transduce and identify optotagged neurons in the granular layer constrains our efforts to discern cell type-specific contributions across cortical microcircuits. Future efforts to develop AAVs containing layer-specific gene regulatory elements will greatly facilitate the investigation of laminar cortical computations in macaques.*

In Discussion, under "Future directions"

Our study motivates several lines of future investigation, both within and beyond visual cortical area V4. First, it is valuable to compare the responses of V4 excitatory and inhibitory neurons across a broader array of visual stimuli, and across different behavioral states; such studies may clarify the contribution of visual and other inputs to the responses of each neuronal cell type. Second, it is valuable to compare the properties of V4 excitatory and inhibitory neurons across different cortical layers; such studies may clarify how the two neuronal cell types uniquely contribute to visual processing in specific microcircuits. Third, it is valuable to probe functional interactions between V4 excitatory and inhibitory neurons, and between optotagged neurons and surrounding neurons; such studies may clarify principles of cortical processing at the population-level. Fourth, it is valuable to compare the response properties of inhibitory neurons across many visual cortical areas; such studies are necessary for evaluating the specificity and generality of inhibitory neuron function across stages of information processing. Fifth, it is valuable to expand the optotagging approach to study the functional properties of molecularly distinct inhibitory neurons; such studies will advance a more comprehensive understanding of how inhibitory circuits influence cortical function in macaques.

The authors' treatment of the existing literature on inhibitory interneurons is disappointingly thin and should be expanded to provide a richer context for the current findings. At this point, most of what we know about the sensory response properties of GABAergic interneurons comes from the extensive rodent literature, and the authors should put their findings in the context of that knowledge. The authors' entire argument about their findings rests on their claim that interneurons in rodents are tuned, making the lack of selectivity in the primate surprising. In fact, most studies, including those in cat V1, make a strong case for a lack of selectivity in interneurons across cell types. In cat V1, FS cells are significantly less selective than RS cells across all layers. In mouse, PV and SST interneurons in V1 and S1 are less tuned, and in some cases entirely untuned, for visual stimulus features such as orientation. PV cells are more tuned than SST and VIP cells for spatial frequency, but less tuned than pyramidal neurons (see DiPoppa et al., 2018, Millman et al., 2020, deVries et al 2019, etc). The single exception may be in the domain of visual contrast, where most existing data suggests equivalent contrast sensitivity for excitatory and inhibitory cells (but see Millman et al., for VIP contrast responses). Although no data exist on interneurons tuning for higher-order features in rodents, the overall bulk of the existing data suggest that interneurons are not as selective as excitatory neurons. The authors' current findings thus generally fit well with previous literature.

We thank the reviewer for encouraging us to link our findings to those made in other animal models. We have now have followed the reviewer's suggestion in an expanded Discussion section, and we include the relevant citations generously shared by the reviewer . The revised text now reads as follows:

Much of what we know about the role of cortical inhibitory neurons in visual information processing comes from studies conducted in other species, thereby motivating comparisons between their findings and ours in macaques. Inhibitory neurons in cortical area V1 of mice, cats and rabbits are reported to be selective for many basic visual properties, albeit more broadly selective than excitatory neurons (e.g. for orientation, spatial frequency, size and contrast)[16-29]. Inhibitory neurons in V1 of these other species are also reported to be strongly responsive to visual stimuli[14,28,30,31]. Thus, our finding that inhibitory neurons in cortical area V4 of macaques have weaker contour shape selectivity than excitatory neurons generally agrees with observations made in other species. In contrast, our finding that many inhibitory neurons are not strongly responsive to visual stimuli seems to deviate from observations made in other species. The weaker stimulus-evoked responses of inhibitory neurons need not imply that inhibition in V4 is weak relative to excitation, as this balance depends also on the unknown synaptic strengths and the correlations among inputs to individual neurons. Additionally, as cortical areas appear to operate in a "loosely balanced" regime[32], there is some flexibility in the requirements for excitatory/inhibitory balance. The paucity of visually responsive inhibitory neurons in our study suggests that inhibitory neurons in higher areas of the primate cerebral cortex may

contribute to visual information processing in fundamentally different ways than do excitatory neurons.

One aspect where the authors' results are quite surprising is the relatively modest percentage of interneurons that show sensory responses. The authors develop this portion of the study using optogenetic depolarization to enhance the responses of these cells. It is not accurate to call these cells 'silent', since they could not be identified unless they were periodically active, and the authors should use a different label. Nevertheless, these results are very intriguing, given the consensus model of cortical circuit function where input to a circuit always evokes yoked excitation and inhibition, with excitation and inhibition always being fairly well balanced. In contrast, the current manuscript makes a case for considerably less inhibition than excitation being evoked in response to a driving stimulus in V4. The authors should flesh out how the low rate of sensory-evoked inhibitory firing does or does not violate the canonical feedforward E-I model.

We thank the reviewer for making these recommendations for further improvement. We have followed the reviewer's two suggestions as follows:

Silent neurons

We no longer label these neurons as "silent" in the revised manuscript. Instead, we refer to these neurons more agnostically as "unresponsive".

E-I models

We are grateful for the opportunity to expand on how our findings may be interpreted in the context of the canonical E-I model. While our single neuron measurements are not ideally suited to infer the nature of the balance between excitation and inhibition in V4, we emphasize that balance in cortical circuits can come about in different ways. For example, E/I balance can be achieved by excitation and inhibition having equal firing rates or equal synaptic strengths. Thus, the relevant quantity of interest is likely the total inhibitory current injected into neurons. As the reviewer suggests, the standard model of E/I balance assumes a "tight balance", where the excitation and inhibition that cancel are much larger than the residual input that remains after cancelation (e.g. van Vreeswijk and Sompolinsky, 1996, 1998). However, there is now substantial experimental and theoretical evidence that the cerebral cortex likely operates in a "loose balance" regime, where the canceling inputs are comparable in size to the remaining residual inputs (as reviewed in detail by Ahmadian and Miller, 2021). It is therefore likely that cortical area V4 operates in such a "loose balance" regime. Future experiments using intracellular recordings (e.g. Priebe and Ferster, 2008) and causal perturbations of inhibitory neuron activity while recording from other neurons (e.g. Sanzeni et al, 2020) are needed to investigate the detailed nature of E/I balance in V4. Given that our data cannot support clear conclusions about E/I balance, we are concerned that including all of the above details in the manuscript may confuse readers unnecessarily. Instead, we have elected to add the following sentences to gently address the point on E/I balance in the revised Discussion section:

The weaker stimulus-evoked responses of inhibitory neurons need not imply that inhibition in V4 is weak relative to excitation, as this balance depends also on the unknown synaptic strengths and the correlations among inputs to individual neurons. Additionally, as cortical areas appear to operate in a "loosely balanced" regime[32], there is some flexibility in the requirements for excitatory/inhibitory balance.

References

1 El-Shamayleh, Y., Kojima, Y., Soetedjo, R. & Horwitz, G. D. Selective Optogenetic Control of Purkinje Cells in Monkey Cerebellum. *Neuron* 95, 51–62 (2017).

2 Ohayon, S., Grimaldi, P., Schweers, N. & Tsao, D. Y. Saccade modulation by optical and electrical stimulation in the macaque frontal eye field. *J Neurosci* 33, 16684–16697 (2013).

3 Moore, T. & Armstong, K. M. Selective gating of visual signals by microstimulation of frontal cortex. *Nature* 421, 370–373 (2003).

4 Armstong, K. M. & Moore, T. Rapid enhancement of visual cortical response discriminability by microstimulation of the frontal eye field. *PNAS* 104, 9499–9504 (2007).

5 Ekstorm, L. B., Roelfsema, P. R., Arsenault, J. T., Bonmassar, G. & Vanduffel, W. Bottom-up dependent gating of frontal signals in early visual cortex. *Science* 321, 414–417 (2008).

6 Yau, J. M., Pasupathy, A., Brincat, S. L. & Connor, C. E. Curvature processing dynamics in macaque area V4. *Cereb Cortex* 23, 198–209 (2013).

7 Gattass, R., Sousa, A. P. & Gross, C. G. Visuotopic organization and extent of V3 and V4 of the macaque. *J Neurosci* 8, 1831–1845 (1988).

8 El-Shamayleh, Y., Ni, A. M. & Horwitz, G. D. Genetic strategies for targeting primate neural circuits. *J Neurophysiol* 116, 122–134 (2016).

9 El-Shamayleh, Y. & Horwitz, G. D. Primate optogenetics: Progress and prognosis. *PNAS* 116, 26195–26203 (2019).

10 De, A., El-Shamayleh, Y. & Horwitz, G. D. Fast and reversible neural inactivation in macaque cortex by optogenetic stimulation of GABAergic neurons. *Elife* 9 (2020).

11 Nowak, L. G., Azouz, R., Sanchez-Vives, M. V., Gray, C. M. & McCormick, D. A. Electrophysiological classes of cat primary visual cortical neurons in vivo as revealed by quantitative analyses. *J Neurophysiol* 89, 1541–1566 (2003).

12 Cardin, J. A., Palmer, L. A. & Contreras, D. Stimulus Feature Selectivity in Excitatory and Inhibitory Neurons in Primary Visual Cortex. *J Neurosci* 27, 10333–10344 (2007).

13 Trainito, C., von Nicolai, C., Miller, E. K. & Siegel, M. Extracellular Spike Waveform Dissociates Four Functionally Distinct Cell Classes in Primate Cortex. *Curr Biol* 29, 2973–2982 (2019).

14 Niell, C. M. & Stryker, M. P. Highly selective receptive fields in mouse visual cortex. *J Neurosci* 28 (2008).

15 Reynolds, J. H., Pasternak, T. & Desimone, R. Attention increases sensitivity of V4 neurons. *Neuron* 26, 703–714 (2000).

16 Hirsch, J. A. *et al.* Functionally distinct inhibitory neurons at the first stage of visual cortical processing. *Nat Neurosci* 6, 1300–1308 (2003).

17 Sohya, K., Kameyama, K., Yanagawa, Y., Obata, K. & Tsumoto, T. GABAergic neurons are less selective to stimulus orientation than excitatory neurons in layer II/III of visual cortex, as revealed by in vivo functional Ca2+ imaging in transgenic mice. *J Neurosci* 27, 2145–2149 (2007).

18 Cardin, J. A., Palmer, L. A. & Contreras, D. Cellular mechanisms underlying stimulus-dependent gain modulation in primary visual cortex neurons in vivo. *Neuron* 59, 150–160 (2008).

19 Kerlin, A. M., Andermann, M. L., Berezovskii, V. K. & Reid, R. C. Broadly tuned response properties of diverse inhibitory neuron subtypes in mouse visual cortex. *Neuron* 67, 858–871 (2010).

20 Atallah, B. V., Bruns, W., Carandini, M. & Scanziani, M. Parvalbumin-expressing interneurons linearly transform cortical responses to visual stimuli. *Neuron* 73, 159–170 (2011).

21 Adesnik, H., Bruns, W., Taniguchi, H., Huang, Z. J. & Scanziani, M. A neural circuit for spatial summation in visual cortex. *Nature* 490, 226–231 (2012).

22 Lee, S. H. *et al.* Activation of specific interneurons improves V1 feature selectivity and visual perception. *Nature* 488, 379–383 (2012).

23 Nienborg, H. *et al.* Contrast dependence and differential contributions from somatostatin- and parvalbumin-expressing neurons to spatial integration in mouse V1. *J Neurosci.* 33, 11145–11154 (2013).

24 Callaway, E. M. in *Micro-, Meso- and Macro-Connectomics of the Brain* (eds H. Kennedy, D. C. van Essen, & Y. Christen) (Springer, 2016).

25 Dipoppa, M. *et al.* Vision and Locomotion Shape the Interactions between Neuron Types in Mouse Visual Cortex. *Neuron* 98, 602–615 (2018).

26 Keller, A. J. *et al.* A Disinhibitory Circuit for Contextual Modulation in Primary Visual Cortex. *Neuron* 108, 1181–1193 (2020).

27 Millman, D. J. *et al.* VIP interneurons in mouse primary visual cortex selectively enhance responses to weak but specific stimuli. *Elife* 9 (2020).
28 de Vries, S. E. J. *et al.* A large-scale standardized physiological survey reveals functional organization of the mouse visual cortex. *Nat Neurosci* 23, 138–151 (2020).
29 Furguson, K. A. *et al.* VIP interneurons regulate cortical size tuning and visual perception. *Cell Rep.* 42, 113088 (2023).
30 Zhuang, J. *et al.* Layer 4 in primary visual cortex of the awake rabbit: contrasting properties of simple cells and putative feedforward inhibitory interneurons. *J. Neurosci* 33, 11372–11389 (2013).
31 Su, C., Mendes-Platt, R. F., Alonso, J. M., Swadlow, H. A. & Bereshpolova, Y. Fast-Spike Interneurons in Visual Cortical Layer 5: Heterogeneous Response Properties Are Related to Thalamocortical Connectivity. *J Neurosci* 45, 1–11 (2025).
32 Ahmadian, Y. & Miller, K. D. What is the dynamical regime of cerebral cortex? *Neuron* 109, 3373–3391 (2021).

RESPONSE TO REVIEWERS

NCOMMS-26-000342-A

Optotagging in primate cortex uncovers excitatory and inhibitory neuron specializations in form vision

Chuyi Su & Yasmine El-Shamayleh

We thank the reviewers for their thoughtful feedback and suggestions for further improvement. Below, please find our brief responses (in blue) to each of the reviewers' latest comments (in black).

Reviewer #1

The authors have done an excellent job with their revised manuscript. I particularly appreciate their careful approach to acknowledging limitations rather than overclaiming when results on certain aspects remain partly inconclusive. This intellectual honesty strengthens rather than weakens the work. We should not forget that Rome is not built on one day... The study itself is exceedingly valuable, establishing a foundation for future research in this area of opto-tagging in NHP. I highly recommend this manuscript for publication in Nature Communications and commend the authors!

We thank Reviewer #1 for their positive feedback and encouragement. Additionally, we agree that while the study has both virtues and limitations, it serves as a fruitful starting point for leveraging optotagging approaches in monkeys to advance a deeper mechanistic understanding of primate brain function.

Reviewer #2

In their revised manuscript, the authors have addressed the major concerns raised in my original comments through additional analyses, new supplementary figures, and more cautious interpretation of several key findings. While some methodological limitations remain inherent to the dataset (e.g., quantification of transduction efficiency, laminar coverage, and inhibitory subtype specificity), these limitations are now appropriately acknowledged and discussed. I have no major remaining concerns and support publication after minor revisions.

We thank Reviewer #2 for their valuable feedback, which continues to strengthen the manuscript. We have now addressed all of the reviewer's minor comments, as elaborated below.

Minor comments

1. The additional analyses substantially strengthen the conclusions regarding temporal dynamics. Although a normalized or statistical-onset latency measure would provide a more criterion-independent estimate of tuning emergence, I do not consider this essential for publication.

We have followed the reviewer's suggestion and re-analyzed the data these data in a new way. Specifically, we now define time–to–selectivity as the first time point when the selectivity index reached 90% of its final value for two consecutive time bins. This analysis confirmed that the time–to–selectivity for bar orientation occurred fairly early in V4 responses whereas time–to–selectivity for contour shape emerged much later in time ($p < 0.0001$; median was 70 ms for bar orientation and 120 ms for contour shape). Additionally, we repeated this new analysis using 80% of the final selectivity value as a criterion and obtained qualitatively similar results.

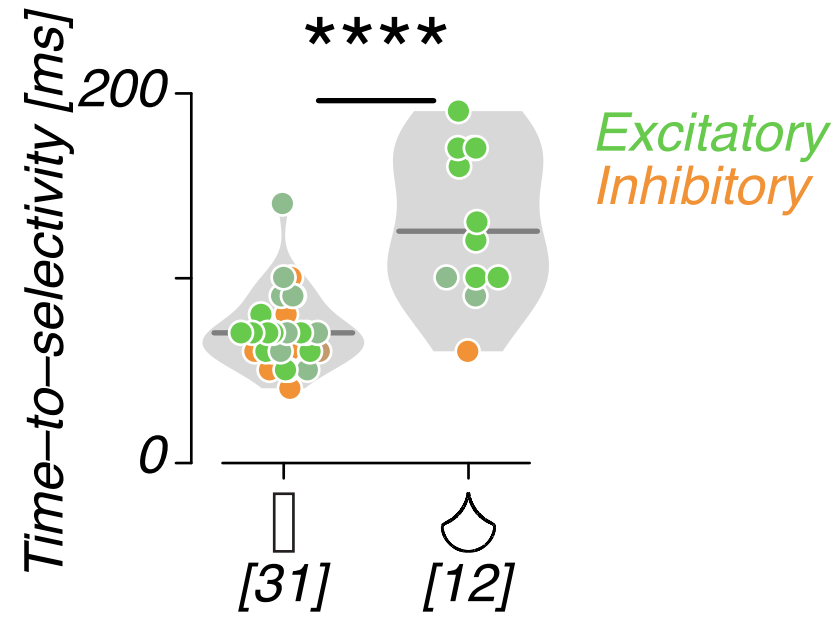

Supplementary Figure S13. Time course of visual form selectivity. (C) Distribution of time–to–selectivity measures for neurons selective for bar orientation and contour shape (data are color-coded by neuronal cell type and activation latency). In this case, time–to–selectivity was defined as the first time point when the selectivity index reached 90% of its final value for two consecutive time bins.

We have now incorporated this supporting analysis into **Supplementaru Figure S13**. We also mention the result of this new analysis in the revised Methods and Results section as follows.

In Methods

For additional verification of our findings, we also used a different measure of the temporal dynamics of visual form selectivity. In this case, time–to–selectivity was defined as the first time point at which the selectivity index reached 90% (or 80%) of its final value for two consecutive time bins; this analysis yielded qualitatively similar results (Supplementary Figure 13C).

In Results

*To ensure that these findings did not depend on how we quantified time–to–selectivity, we repeated the analyses using a different measure (see **Methods**) and obtained qualitatively similar results (**Supplementary Figure S13C**; for all neurons, p<0.0001; median was 70 ms and 120 ms for bar orientation and contour shape).*

2. Typo, page 5: 'The functional similarities across animals demonstrates...': demonstrate.

3. Typo, page 9: 'we reasoned that a sensible proxy would be to examined...': examine

4. Typo, page 10: 'These findings suggest that information about bar orientation may already present...': may already present

We thank the reviewer for catching these three typos, which we have now corrected.

5. Page 10: This section is redundant, 'Similarly, we also recorded optotagged neurons that were visually responsive and unresponsive neurons across cortical depth...'

We thank the reviewer for this helpful suggestion.We have now combined this sentence with the previous one to reduce redundancy as follows:

*Specifically, we found neurons that were selective for simple and complex forms, as well as neurons that were visually responses and unresponsive at various depths (**Supplementary Figure S9C**).*

Reviewer #3

The authors have largely addressed my concerns, I think this paper is a nice addition to the modern primate cortical literature.

If I may make a suggestion for the future, I strongly encourage the authors to think more broadly about capturing and analyzing both units and fields in their datasets, particularly when it is possible to identify cell classes using optotagging. It would be enormously valuable for the field to have more extensive primate cortical electrophysiological datasets with both multiple single units and field potentials.

We thank Reviewer #3 for their constructive feedback. We wholeheartedly agree that obtaining paired optotagging and LFP recordings is a key direction for future investigation. We wish to point out, however, that

such a dataset would require additional technical innovations in primates. To-date, it remains challenging to combine optotagging approaches with larger scale neural recordings (e.g. using laminar probes or Neuropixels) in macaques. Some of the challenges arise from difficulties in inserting optical fibers and laminar electrodes simultaneously through the thick dura mater of macaques, as well as delivering sufficient light that can reach multiple recording contacts. Several of our colleagues have been working towards this goal and we hope to join these efforts soon. For the benefit of our readers, we now further emphasize the reviewer's point in the revised Discussion section as follows:

*We were also unable to probe functional interactions between optotagged neurons and other surrounding neurons, with the exception of one experimental session where we were fortunate to record two well-isolated neurons simultaneously (see **Supplementary Figure S14**). Future studies combining cell type-specific optogenetics with large-scale neural recordings across cortical layers are therefore needed to yield such mechanistic insights. In particular, it would be useful to simultaneously record single optotagged neurons and local field potentials, as such datasets would enable valuable cross-species comparisons of cellular and circuit mechanisms between monkeys and mice.*
